# Drivers of stunting reduction in the Kyrgyz Republic: A country case study

**DOI:** 10.1093/ajcn/nqaa120

**Published:** 2020-07-16

**Authors:** Jannah M Wigle, Nadia Akseer, Roman Mogilevskii, Samanpreet Brar, Kaitlin Conway, Zalina Enikeeva, Mariia Iamshchikova, Muhammad Islam, Dilbara Kirbasheva, Aviva I Rappaport, Hana Tasic, Tyler Vaivada, Zulfiqar A Bhutta

**Affiliations:** Centre for Global Child Health, Hospital for Sick Children, Toronto, Canada; Dalla Lana School of Public Health, University of Toronto, Toronto, Canada; Centre for Global Child Health, Hospital for Sick Children, Toronto, Canada; Dalla Lana School of Public Health, University of Toronto, Toronto, Canada; Institute of Public Policy and Administration, University of Central Asia, Bishkek, Kyrgyz Republic; Centre for Global Child Health, Hospital for Sick Children, Toronto, Canada; Centre for Global Child Health, Hospital for Sick Children, Toronto, Canada; Institute of Public Policy and Administration, University of Central Asia, Bishkek, Kyrgyz Republic; Institute of Public Policy and Administration, University of Central Asia, Bishkek, Kyrgyz Republic; Centre for Global Child Health, Hospital for Sick Children, Toronto, Canada; Institute of Public Policy and Administration, University of Central Asia, Bishkek, Kyrgyz Republic; Centre for Global Child Health, Hospital for Sick Children, Toronto, Canada; Centre for Global Child Health, Hospital for Sick Children, Toronto, Canada; Centre for Global Child Health, Hospital for Sick Children, Toronto, Canada; Centre for Global Child Health, Hospital for Sick Children, Toronto, Canada; Dalla Lana School of Public Health, University of Toronto, Toronto, Canada; Center of Excellence in Women and Child Health, the Aga Khan University, Karachi, Pakistan

**Keywords:** stunting, linear growth, children, nutrition, exemplar, Kyrgyz Republic, Central Asia, mixed methods

## Abstract

**Background:**

Chronic malnutrition among infants and children continues to represent a global public health concern. The Kyrgyz Republic has achieved rapid declines in stunting over the last 20 y, despite modest increases in gross domestic product per capita.

**Objective:**

This study aimed to conduct a systematic, in-depth assessment of national, community, household, and individual drivers of nutrition change and stunting reduction, as well as nutrition-specific and -sensitive policies and programs, in the Kyrgyz Republic.

**Methods:**

This mixed methods study employed 4 inquiry methods, including: *1*) a systematic scoping literature review; *2*) retrospective quantitative data analyses, including linear regression multivariable hierarchical modeling, difference-in-difference analysis, and Oaxaca–Blinder decomposition; *3*) qualitative data collection and analysis; and *4*) analysis of key nutrition-specific and -sensitive policies and programs.

**Results:**

Stunting prevalence has decreased in the Kyrgyz Republic, however, subnational variations and inequities persist. Child growth Victora curves show improvements in height-for-age z-scores (HAZ) for children in the Kyrgyz Republic between 1997 and 2014, indicating increased intrauterine growth and population health improvements. The decomposition analysis explained 88.9% (0.637 SD increase) of the predicted change in HAZ for children under 3 y (1997–2012). Key factors included poverty (61%), maternal nutrition (14%), paternal education (6%), fertility (6%), maternal age (3%), and wealth accumulation (2%). Qualitative analysis revealed poverty reduction, increased migration and remittances, food security, and maternal nutrition as key drivers of stunting decline. Systematic scoping literature review findings supported quantitative and qualitative results, and indicated that land reforms and improved food security represented important factors. Key nutrition-specific and -sensitive policies and programs implemented involved breastfeeding promotion, social protection schemes, and land and health sector reforms.

**Conclusions:**

Improvements in stunting were achieved amidst political and economic changes. Multilevel enablers, including poverty reduction, improved food security, and introduction of land and health reforms have contributed to improvements in health, nutrition, and stunting among children in the Kyrgyz Republic.

## Introduction

**FIGURE 1:**
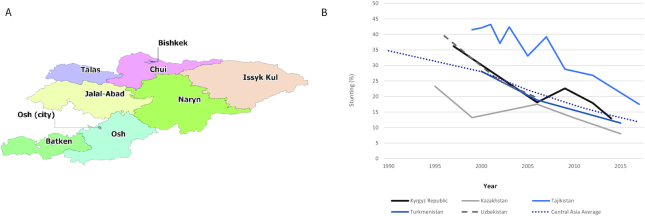
A) Map of the Kyrgyz Republic. B) The prevalence of children aged under 5 y stunting in Central Asian countries, 1990–2017. Source: (1)

Global efforts to address chronic malnutrition among infants and children have contributed to drastic improvements in child stunting, as the prevalence of stunting among children has decreased by 42% worldwide, between 1990 and 2017, from 39.3% to 22.2% (1). The Kyrgyz Republic is a landlocked country in Central Asia, with an estimated population of 6.39 million (2), with the majority of the population living in rural areas (**Figure 1**A). It is a largely mountainous country and experiences drastic climatic patterns due to varying altitude and temperatures (3, 4).

The Kyrgyz Republic has experienced rapid declines in stunting over the last 20 y, as prevalence rates among children aged under 5 y decreased by nearly one-third from 36.2% in 1997, to 18.1% in 2005, and subsequently 12.9% in 2014. This rate of decline has been greater in comparison to most other Central Asian countries (Turkmenistan, Uzbekistan, Kazakhstan, and Tajikistan), as well as the regional average (Figure 1B) (1).

Since achieving independence in December 1991 after the collapse of the Soviet Union, the Kyrgyz Republic has faced substantial political and economic changes, including democratization and rapid economic decline, followed by gradual economic gains. Reductions in stunting were achieved despite periods of political instability and changes in national governments, including the 2005 Tulip Revolution (5–9) and the Second Kyrgyz Revolution in 2010 (10, 11). Poverty has declined rapidly in the Kyrgyz Republic since the late 1990s, as the poverty gap at $3.20/d decreased from 32.2% of the population in 2000 to 3.5% in 2017 [2011 purchasing power parity (PPP)] (12). After initial declines in gross domestic product (GDP) per capita following the Soviet Union collapse, steady increases are observed with rates doubling from $1644 to $3557 per capita PPP between 2000 to 2016 (12). Literacy is nearly universal among both males and females. However, some improvements in gender equality were achieved as measured by the gender development index over the 1990–2016 period. Open defecation is uncommon in the Kyrgyz Republic, however, modest improvements in access to improved sources of drinking water were achieved over this same period (**Supplementary Appendix 1**). Improvements in indicators related to gender have been achieved, as rates of child marriage decreased from 21.2% in 1997 to 11.6% in 2014. Although the overall fertility rate has increased from 2.4 in 2000 to 3.2 children per woman in 2015, the adolescent fertility rate improved dramatically from 68.8 in 1992 to 38.8 per 1000 women aged 15–19 y in 2016 (12).

Despite observed and notable improvements in health and nutrition in the Kyrgyz Republic, no systematic assessment of stunting reduction determinants has been conducted to date. Few studies have specifically explored the association between drivers and height-for-age z-score (HAZ) or weight-for-age z-score (WAZ) and no studies employed a mixed methods approach (see **Panel 1, Supplementary Appendix 2**) (3, 6–9, 11, 13–54). Research on the impact of land reforms showed a significant association between land privatization and both HAZ and WAZ, however, this study was based on outdated cross-sectional survey data from several Living Standards and Measurement Surveys conducted during 1993–1998 (27).

Panel 1 **Systematic Literature Review of Stunting Determinants**

The systematic literature review identified many basic causes that may have contributed to stunting decline in Kyrgyz Republic. This began with the collapse of the Soviet Union and transition to democracy (1–4). Overall, there were improvements in other basic causes including GDP growth (5–9), increased spending on social services (10–14), the development of land reform laws (10, 15), higher agricultural outputs (15, 16), seasonal labour migration (17), and improvements in primary health care through programs, such as the Manas initiatives (6, 18). Periods of economic and agricultural growth coincided with stunting reductions at a population level (19). The levels of poverty declined from 32.2% in the late 1990s to 3.5% in 2017 (2011 PPP) (14). Similarly, Kyrgyz Republic economy (GDP/capita) improved rapidly between 2000 and 2006 (21). These improvements may be due to national cash transfer programs, poverty reduction frameworks (22) and land privatization. Land privatization was associated with 13% of the variation in child HAZ outcome in one study (23). Increased GDP at the national level resulted in increased total health expenditure from 2000–2014, from 4.7% to 6.5% of GDP, leading to decreased out-of-pocket health expenditure and increased health service utilization (6, 14, 24).

Important underlying causes of stunting in the literature included access to safe water (25, 26), maternal education (27), and food security and availability. National food availability of grains, livestock and poultry, milk and eggs increased over the period of stunting reduction (16). Improvements in dietary intake were observed between 1999 and 2015 where there was an improvement in mean energy supply (kcal) going from 107% to 120% (28). The percentage of the population suffering from energy inadequacy declined from 16% in 1992 to 6% in 2014. This may have contributed to the declining prevalence of undernutrition from 16.3% in 1999–2001 to 6.4% in 2014–2016 (28).

The immediate causes of stunting in Kyrgyz Republic in literature are childhood infection (5, 27, 30–32), maternal age and height (33), dietary intake (34, 35), and breastfeeding (36–38). Therefore, implementation of nutrition specific programs at a national level may have contributed to decreased rates of stunting. Health reforms, such as the *Healthy Nation State Plan* (39–41) *, Healthy Children at the Level of Primary Health Care* [12], [13]*, Manas* (6, 14, 42, 43)*, Manas Taalimi* (3, 6, 11, 26, 42, 43), *Den Sooluk* (12, 43–45), and the *Integrated Management of Childhood Illness* (IMCI) program (46) all included breastfeeding promotion and infection reduction strategies (40, 42, 45–47). In addition, the national vitamin A supplementation program may have indirectly led to a decline in stunting by decreasing the prevalence of infection (27, 48–50).

To address this gap in the literature, this study aimed to conduct a systematic, in-depth mixed methods assessment to identify potential national, community, household, and individual drivers of stunting reduction, as well as relevant nutrition-specific and -sensitive policies and programs, in the Kyrgyz Republic from 1997 to 2014. Efforts were focused on exploring and understanding important nutritional transitional periods (1997 to 2005/2006, 2005/2006 to 2014). This mixed methods study aimed to: *1*) quantitatively examine key determinants of stunting and to breakdown long-term stunting change into relative contributions from key factors; *2*) explore national and community perspectives and experiences related to the nutrition evolution; and *3*) generate a systematic overview and analysis of pivotal nutrition-specific and -sensitive policies and programs.

## Methods

### Study design

**FIGURE 2:**
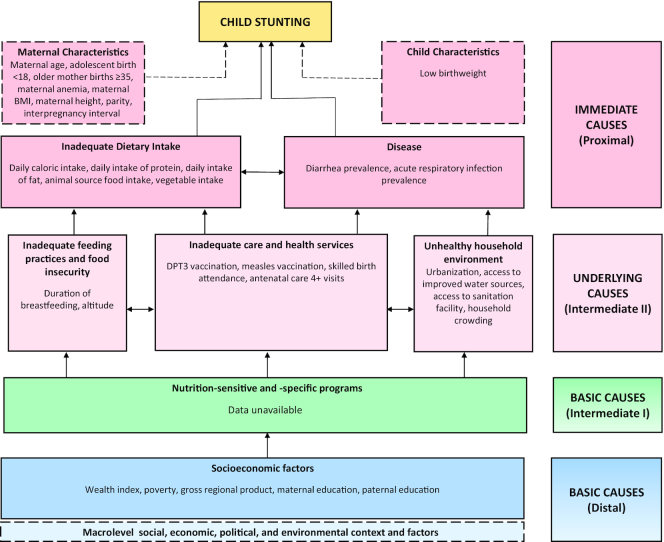
Conceptual framework showing distal, intermediate, and proximal determinants of stunting in the Kyrgyz Republic. Note: framework reflects only indicators that were measurable and available for quantitative analysis. DPT3, diphtheria-tetanus-pertussis.

This mixed methods study was informed by 4 types of inquiry including: *1*) a systematic scoping review of peer-reviewed and gray literature; *2*) retrospective quantitative data analyses; *3*) prospective qualitative data collection and analysis; and *4*) analysis of key nutrition-specific and -sensitive policies and programs. A conceptual framework was adapted based on the nutrition frameworks developed by Black (55) and UNICEF (56) and informed both the quantitative and qualitative analyses (**Figure 2**). The framework used across all case studies in this project is summarized in the methods article within this supplement (57).

Ethics approval for the study, inclusive of primary data collection, was obtained through the University of Central Asia's research ethics process. Ethics approval for the broader stunting case study was also obtained through the Research Ethics Board at the Hospital for Sick Children (SickKids), in Toronto, Canada.

### Systematic scoping literature review

A systematic scoping literature review of published peer-reviewed and gray literature in the Kyrgyz Republic was conducted between July 2017 and July 2018 to identify and synthesize evidence on potential contextual factors and key policies and programs that may have contributed to improvements in child nutrition or stunting reduction. Search terms employed include “stunting” or “linear growth” or “linear growth stunting” or “HAZ” or “height” or “height-for-age” or “LAZ” or “length” or “length-for-age” or “undernutrition” or “malnutrition” or “nutr*” or “health” and “child*” or “infan*” and “Kyrgyz Republic” or “Kyrgyzstan” or “Kirghiz S.S.R.” or “Kirghiz Soviet Socialist Republic” or “Kirghizia” or “Kyrgyz*.” A search for indexed published peer-reviewed literature was conducted using >15 online databases and these were double-screened against inclusion criteria (see Supplementary Appendix 2 for databases). A manual search of reference lists and organizational websites was also utilized by multiple reviewers to identify relevant gray literature sources. The design and implementation of our systematic scoping review was informed and guided by the Preferred Reporting Items for Systematic Reviews and Meta-Analyses (PRISMA) checklist (58). An initial search of databases found 592 records; after removing duplicates and screening according to titles and abstracts, a total of 127 records were included in our analysis (Supplementary Appendix 2 and **Supplementary Systematic Scoping Literature Review Data**).

### Quantitative methods

#### Data sources

The main quantitative datasets used were the Kyrgyz Republic's Multiple Indicator Cluster Surveys (MICS) and Demographic and Health Surveys (DHS) (1997–2014). These multistage complex surveys provided nationally and regionally representative estimates for key health indicators. Sample sizes for children aged under 5 y by age group and survey round are presented in **Table 1**.

**TABLE 1:** Sample size by survey year based on valid child anthropometric data

	Year of survey			
Age group	DHS 1997	MICS 2005/2006	DHS 2012	MICS 2014
<6 mo	149	264	383	402
6–23 mo	457	760	1216	1201
24+ mo	222*	1088	1188	1275
<36 mo	828	1445	2096	2135
<5 y		2112	2787	2878

*24–35 mo.

Note: based on index (youngest) child data.

DHS, Demographic and Health Survey; MICS, Multiple Indicator Cluster Survey.

#### Outcomes and covariables

The main outcomes for this study were child HAZ and stunting prevalence (HAZ < −2SD), estimated from using WHO child growth standards (59). Covariables were selected in line with Figure 2 as available in MICS/DHS surveys (individual/household variables) and the Life in Kyrgyzstan Surveys and National Statistical Committee data respositories (60, 61), which include a range of national and regional data for the country. Ecological (oblast-level) estimates aligning with our analysis years were incorporated into the study datasets. Factors corresponding to “basic causes,” “underlying causes,” and “immediate causes” were analyzed as distal, intermediate, and proximal predictors of child stunting reduction (Figure 2).

#### Statistical analysis

Population shifts in child growth faltering were analyzed using HAZ kernel density plots for all survey years. We used smoothed local polynomial regressions to estimate child HAZ compared with age curves (“Victora curves”) to understand the growth faltering process from birth to age 5 y (62). Statistical slopes and inflection points of Victora curves were estimated using piece-wise linear splines. We analyzed stunting prevalence inequalities by wealth quintile (Q1–Q5), maternal education, area of residence (urban, rural), and child gender using standardized and well-established methods (63). Principal components analysis using household asset data were used to generate wealth scores, which were subsequently organized into quintiles. To assess change in wealth inequality over time accounting for the entire asset index distribution, we also estimated the Slope Index of Inequality (SII) and Concentration Index (CIX) which respectively reflected absolute and relative socioeconomic inequalities (64, 65). The relative change in stunting prevalence of each oblast in the Kyrgyz Republic was estimated using compound annual growth rates (CAGR) (66).

Linear regression-based multivariable hierarchical modeling of distal, intermediate, and proximal level covariables (56) was undertaken to identify key determinants of child HAZ change from 1997 to 2012 (using DHS surveys for comparability). A difference-in-difference (DID) framework, with time*covariable interaction terms, was used to study whether a change in covariables resulted in HAZ change (67). Oaxaca–Blinder decomposition was used to estimate the relative contribution of covariables to mean child HAZ change between 1997 and 2012. Detailed methodology is included in **Supplementary Appendix 3** and the methods article in this supplement (57). All analyses were conducted with Stata 14.0 and accounted for survey design and weighting.

### Policy and program review

The synthesis of key nutrition-specific and -sensitive policies and programs in the Kyrgyz Republic aimed to identify and describe potential policy events (e.g., policies, strategies, laws, and legislation) and evaluate potential contributions to the improvements in nutrition and stunting among children aged under 5 y. The policy analysis provided an overview of key policies/programs, and explored the delivery platform, stakeholders engaged, initiation/scale-up, key program components, monitoring and evaluation of the initiative, source of funding, and success factors and barriers. A timeline from 1994 to 2016 of key nutrition-specific and -sensitive policies and programs in the Kyrgyz Republic was developed in an iterative approach through a review of literature identified during the systematic search. The draft timeline was shared with key informants for validation and to identify gaps in nutrition-related policy efforts. Additional review of literature and manual targeted searches for key documents identified by key informants was conducted, and a revised version of the timeline was proposed. This iterative process continued until consensus was reached among key informants regarding pivotal policy contributions towards stunting decline.

### Qualitative methods

Data collection was conducted between September and December 2017 and multiple research methods were used including in-depth interviews with key informants at national and community levels and focus group discussions (FGDs) with mothers in communities. Using qualitative methods this study aimed to: identify nutrition-specific and -sensitive key events that may have contributed to reduction in stunting in the Kyrgyz Republic; understand success factors and challenges of nutrition-specific and -sensitive policy events; and explore community-level experiences on the nutrition and stunting transition in the Kyrgyz Republic.

Participants were identified and selected using purposive sampling strategies (68), including snowball sampling (69). Stakeholders were purposively selected due to their involvement in the design, implementation, monitoring, or evaluation of nutrition-specific or -sensitive policies and programs at the national level, or based in Talas and Batken oblasts. This approach also ensured diverse national and community perspectives were represented. Selection of focus group communities aimed to achieve coverage of perspectives from diverse geographic regions, including 1 region from the North and 1 region from the South of the Kyrgyz Republic, both of which have achieved substantial reduction of stunting over the last 2 decades. Data collection was conducted until saturation of emerging concepts and themes was achieved.

At the national level (*n* = 20), in-depth interviews were conducted with policymakers, representatives from multi- and bilateral donors and individuals from nongovernmental and civil society organizations. Community key informants (*n* = 16) were also engaged through in-depth interviews in Talas and Batken regions to generate local perspectives on the nutrition transition and included childcare workers and health workers [e.g., social worker, head of kindergarten, physicians, nurses, village health committee (VHC) member]. Five FGDs were conducted in Talas and Batken regions with 69 women who gave birth to children during 1992–1997 and 2012–2017, to compare trends over time relating to key contextual factors and local nutrition transition.

Interviews were audio recorded, transcribed, and translated into English. The conceptual framework presented in Figure 2 informed both the quantitative and qualitative components of the study. Frameworks by Black (55) and UNICEF (56) informed the development of the data collection tools and analysis of qualitative data. Thematic analysis was conducted and explored key themes related to stunting determinants as outlined in our conceptual framework, including socioeconomic status (e.g., education, poverty reduction), migration, health system, water, sanitation and hygiene, as well as dietary intake and behaviors. Reflexivity also represented an important analytic tool in this mixed methods study, as the team reflected on the production of credible and robust results by critically evaluating the research process (e.g., “how, where, when, and by whom data were collected”) and ensuring methodological transparency (70). The Consolidated Criteria for Reporting Qualitative Research (COREQ) checklist was used as an overall guide to reporting of qualitative study results (71). Full qualitative data collection and analysis methods are detailed in **Supplementary Appendix 4**.

## Results

### Descriptive analyses

#### HAZ kernel density plots and Victora curves

**FIGURE 3:**
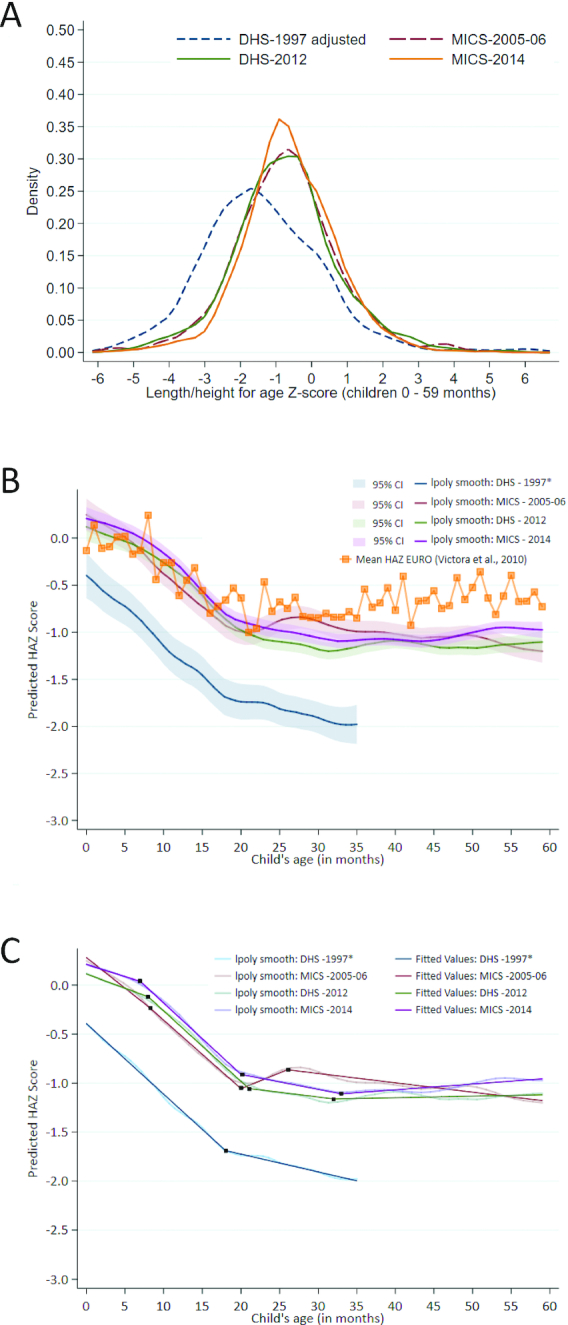
A) Kernel density plot for height-for-age z-score distribution in children aged <5 y, 1997–2014. B) Victora curve using data from the 1997, 2005–06, 2012, and 2014 surveys among children aged <5 y, including WHO Regional Office for Europe (EURO) mean. C) Victora curve using data from the 1997, 2005–2006, 2012, and 2014 surveys among children <5 y with linear splines. lpoly smooth, Kernel-weighted local polynomial smoothing; *child anthropometry data was collected for children <3 years only in DHS 1997. DHS, Demographic and Health Survey; EURO, European Union Region; HAZ, height-for-age z-score; MICS, Multiple Indicator Cluster Survey.

The mean HAZ for children aged under 5 y improved from –1.42 to 0.75 (0.67 SD) between 1997 and 2014 and stunting prevalence followed a similar pattern, decreasing almost 20% points. The kernel density plot for HAZ distribution among children aged under 5 y demonstrates a parallel rightward shift in HAZ between 1997 and 2005/2006, and a narrowing or kurtosis from 2005/2006 and 2012–2014 (**Figure 3**A). A distribution with a higher peak in 2005/2006 suggests improvements and nutritional gains across the entire population, as a greater proportion of children center around a common mean, closer to the international reference population. Limited changes in HAZ distribution were observed between 2005/2006 and 2012, however, narrowing of the curve continued into 2014 with more children closer to the mean HAZ. These plots suggest national improvements in HAZ and reduced population inequalities.

The Victora curves in Figure 3B show that growth trajectory for children in the Kyrgyz Republic improved substantially between 1997 and 2014. The intercept at age 0 mo improved between 1997 and 2005/2006 and was sustained into 2012, suggesting that intrauterine growth and size at birth improved. The 2014 Victora curve is above previous years and has a flatter curve between 0 and 6 mo compared with 2012, indicating potential improvements in breastfeeding among children. Overall, interpretation of this analysis indicates that stunting decline may be attributed to improved maternal nutrition, greater HAZ at birth, and nutrition through breastfeeding for children under 6 mo. Figure 3B shows the Victora curve using data from the 1997, 2005–2006, 2012, and 2014 surveys among children aged <5 y, including the WHO Regional Office for Europe (EURO) mean. The splines in Figure 3C suggest specific inflection points of change in the slope of HAZ from 1997 to 2014; detailed coefficient estimates are in **Supplementary Appendix 5**. The greatest decline in predicted HAZ in 1997 was observed between the ages of 0 and 18 mo at a rate of 0.072 SD per month (95% CI:  −0.073, −0.071). However, in 2014 the decline was delayed, decreasing at a more moderate 0.025 SD per month between birth and 7 mo. The growth faltering period in 2014 began at 7 mo and continued until the age of 20 mo, declining at a similar rate to 1997, at −0.073 SD per month. The absolute value of predicted HAZ remained ≥0.5 SD higher in 2014 than in 1997 for children aged under 36 mo.

### Equity analysis

**FIGURE 4:**
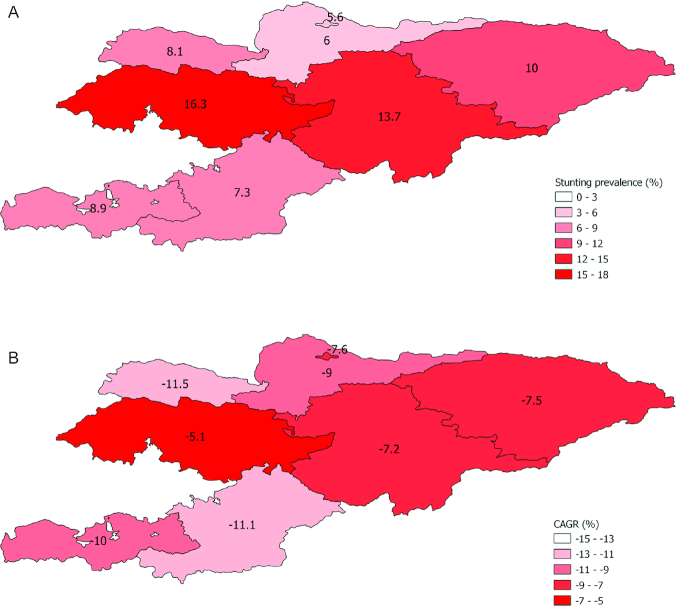
A) Subnational stunting estimates for children aged under 5 y in the Kyrgyz Republic in 2014. B) Compound annual growth rate by oblast, 1997–2014. Child anthropometry data was collected for children <3 years only in the DHS 1997; the following formula was used to estimate % stunting: % stunted under 5 years = -0.0114274 + (1.104429 ^∗^ % stunted under three years) (64).

Stunting prevalence decreased in the Kyrgyz Republic from 36% to 13% between 1997 and 2014, however, reduction was not even subnationally (**Figure 4**A–B, Supplementary Appendix 5). In 1997, Bishkek City had the lowest stunting prevalence (20%), with the highest in Talas oblast (58%). Stunting decreased in 6 out of 7 oblasts by 2014, with Talas and Osh oblasts achieving the greatest declines of 50% (−11.5 CAGR) and 40% (−11.1), respectively. Oblasts with the lowest CAGR included Jalal-Abad (−5.1) and Naryn (−7.2) (Figure 4B).

**FIGURE 5:**
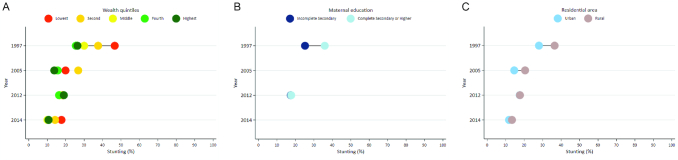
A) Stunting prevalence by wealth quintile, 1997–2014. B) Stunting prevalence by maternal education, 1997–2014. C) Stunting prevalence by residential area, 1997–2014.

Stunting prevalence decreased across all wealth quintiles and the gap between the richest and poorest populations narrowed substantially from 1997 to 2014 (**Figure 5**A). The greatest reductions in wealth inequalities were achieved from 1997 to 2005. Trends in SII and the CIX support these findings (Supplementary Appendix 5, Figure 5A–B).

Maternal education is high in the Kyrgyz Republic, with 89% of women having completed secondary education or higher. As a result, stunting prevalence did not vary substantially by level of education, particularly in recent years (Figure 5B). Reductions in stunting occurred in both rural and urban areas, with rural areas achieving the greatest declines, as the difference in stunting prevalence decreased from 8.5% to 1.6% between 1997 and 2014 (Figure 5C). Stunting prevalence inequalities by gender were minimal (Supplementary Appendix 5).

### Multivariable analyses

**FIGURE 6:**
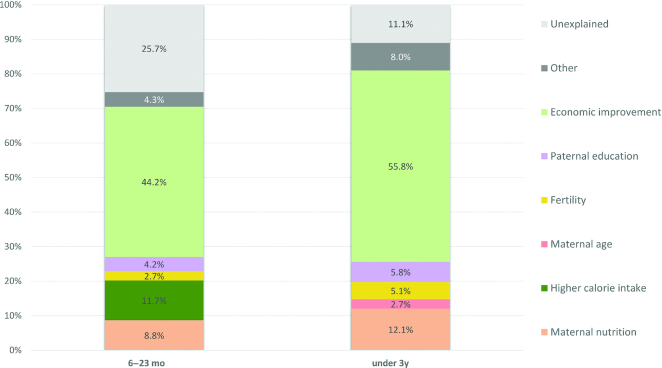
Decomposing predicted changes in height-for-age z-score (i.e., percent contribution of determinant domains) from 1997 to 2012. Note: the under 6-mo age category results are not presented due to small sample sizes and the under 5-y age category due to the 1997 MICS survey not including height-for-age z-score data on children over the age of 35 mo. Indicators included: maternal nutrition (BMI and height), paternal education, higher calorie (daily intake of calories), economic improvement (wealth index and percent of families below national poverty line), maternal age, and fertility (interpregnancy interval and parity). Other category includes child age, gender, and region. CAGR, compound annual growth rate.

The Oaxaca–Blinder decomposition results report that factors explained 88.9% or a 0.637 SD increase of the predicted change in HAZ for children aged under 3 y during 1997–2012 in the Kyrgyz Republic (**Figure 6**); supportive analyses are provided in Supplementary Appendix 5. Poverty contributed the greatest to the amount of explained HAZ (61%), followed by maternal nutrition (14%), paternal education (6%), fertility (6%), maternal age (3%), and wealth accumulation (2%). For the 6–23-mo age group, the decomposition analysis predicted a 0.39 SD improvement in HAZ and in terms of relative contribution of factors represented 71% of the predicted change for 1997–2012. Key drivers of changes in HAZ among this age group included poverty reduction (54%), daily intake of calories (15%), maternal education (12%), paternal education (6%), wealth index (4%), and decreased fertility (3%) (Figure 6). The decomposition analysis suggests that both wealth accumulation and increased maternal education represent key determinants of increased HAZ.

Results from the DID analysis indicate that improvements in poverty (below national lines), diphtheria-tetanus-pertussis vaccination, urbanization, daily caloric intake, daily intake of fats, low birthweight, and maternal age at birth were associated with change in child HAZ from 1997 to 2014. Although wealth index and improved access to sanitation facilities had statistically significant association with HAZ, their interaction terms were not significantly associated with HAZ (**Supplementary Appendix 5**).

#### Policy and program review

**FIGURE 7:**
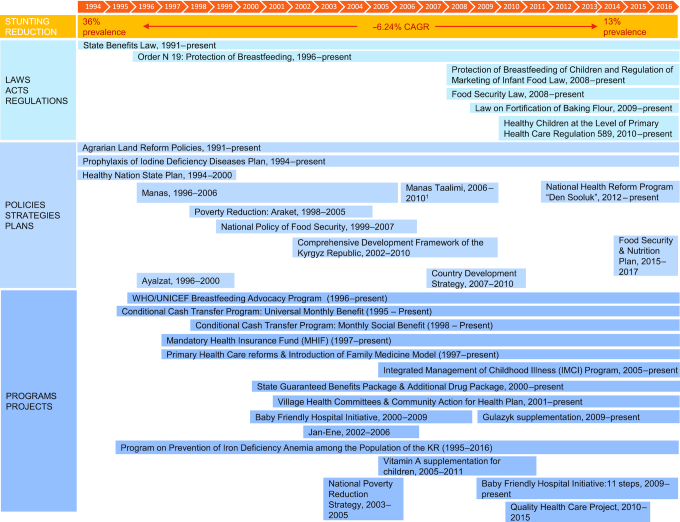
Overview of laws, policies, and programs in the Kyrgyz Republic from 1990–2016. ^1^Policy ended in 2010 but was implemented until Den Sooluk.

Several nutrition-specific and -sensitive policies and programs were implemented between 1994 and 2016 and may have contributed to the reduction of stunting among children (**Figure 7**, **Supplementary Appendix 6**). Influential nutrition-specific efforts involved the adoption of breastfeeding laws (1996–present and 2008–present) and programs (e.g., the Baby-Friendly Hospital Initiative, 2000–present). Substantial nutrition-sensitive initiatives focused on poverty reduction, national development, and land and health system reforms. Key poverty reduction efforts included the introduction of the State Benefits Law (1991–present), conditional cash transfer programs (Universal Monthly Benefit, 1995–present and Monthly Social Benefit, 1998–present), national poverty reduction plan Araket (1998–2005), Comprehensive Development Framework (2002–2010), and National Poverty Reduction Strategy (2003–2005). In addition, the implementation of several agrarian land reforms (1991–present) transformed land distribution and ownership particularly in rural areas in the Kyrgyz Republic after the collapse of the Soviet Union. The adoption of the first health policy, the Healthy Nation State Plan 1994–1999, led to a series of comprehensive health system reforms, including Manas (1996–2006), Manas Taalimi (2006–2010), and Den Sooluk (2012–present). The introduction of the Mandatory Health Insurance Fund (1997–present), primary health care reforms and family medicine model (1997–present), as well as the State Guarantee Benefits Package and Additional Drug Package (2000–present) also represented health system strengthening efforts. Implementation of VHC (2001–present), the Integrated Management of Childhood Illness (IMCI) program (2005–present), and vitamin A supplementation (2005–2011) represented community-based programs and efforts to address health and nutrition. These aimed to increase access to local, primary health services, and address financial barriers to care. Details on each initiative are summarized in Supplementary Appendix 6.

#### Qualitative inquiry results

Across stakeholders, there was a general consensus that socioeconomic improvements and poverty reduction represented critical drivers of improved nutrition and stunting reduction, as a result of increased labor migration, remittances, and household wealth. Improvements in food security and diet diversification were also considered important contributions by stakeholders across levels. National stakeholders emphasized that the adoption of laws and policies on breastfeeding (e.g., Order 19 and the Baby-Friendly Hospital Initiative) improved attitudes and uptake of breastfeeding. Regional stakeholders highlighted several nutrition-related initiatives that they felt were related to nutritional gains, including school feeding and micronutrient supplementation (e.g., Gulazyk micronutrient powder and vitamin A) programs. Both groups of mothers in Batken and Talas oblasts indicated that substantial poverty reduction and increased household wealth represented important driving forces of stunting reduction. In addition, health promotion and the provision of breastfeeding knowledge and support by local VHCs helped to improve nutrition at the community level. Qualitative results are summarized below and in **Supplementary Appendix 7**.

#### National expert stakeholders

In-depth interviews were conducted with national key informants (*n* = 20) and emphasized that key drivers of stunting determinants included diverse distal, underlying, and immediate factors, as well as nutrition-related policy efforts. National key informants highlighted that over the last 20 y, substantial improvements in socioeconomic indicators (e.g., increased wealth accumulation and decreased poverty rates), quality of living, and food security among the population were a result of increased migration and remittances, as well as support from international donors and the Kyrgyz government. Participant #1, a national policymaker, described this improvement: “*[The] situation with chronic malnutrition seems to [have] improved, as the standard of living of the population has risen*.”

Key nutrition-specific and -sensitive initiatives identified by national experts that may have contributed to stunting decline included implementation of breastfeeding policies and programs, micronutrient supplementation (e.g., Gulazyk micronutrient powder and vitamin A program), salt iodization, mandatory flour fortification, IMCI, and social protection programs for low-income families. Underlying factors of improved linear growth related to improved feeding practices and increased food security among communities. Increased diet diversification, caloric intake, and knowledge of appropriate nutrition and food preparation contributed to improved nutrition among women, families, and communities. These findings are supported by Participant #19, a nutrition expert: *“The 1990s was a period which were characterized by strong decline. There were cases when there was nothing to eat. Food was scarce, expensive. Now life has become much better, many products are available, people are more informed.”*

#### Regional stakeholders

Community-level key informants (*n* = 16) were also interviewed in Batken and Talas oblasts. Similarly, community-level stakeholders felt that socioeconomic improvements were associated with declines in stunting in their region, largely due to migration and remittances. Improved access to safe sources of drinking water, particularly in the Southern Kyrgyz Republic, was associated with improvements in community health. Community key informants were responsible for routinely providing nutrition information, guidance, and outreach to families and communities. Despite quantitative evidence indicating slight increases in fertility rates over time, community respondents felt that decreases and the fewer children per woman had contributed to the improved care and nutrition among children and families. An activist from Batken oblast described the importance of decreased fertility: *“Now young people take care of their children very well because they [have fewer] children. Now people are more prosperous than before.”*

#### Mothers in communities

Five FGDs were conducted in villages in Batken and Talas oblasts with 69 women at the community level that had given birth to a child in 1992–1997 and 2012–2017, in order to compare drivers of stunting by location, as well as during important nutritional transition periods. Mothers that had children during 1992–1997 felt that poverty was widespread during the 1990s and that subsequent improvements in quality of life have increased the availability, security, and quality of food. Improved nutrition was associated with increased remittances and opportunities to engage in subsistence farming. Women in this cohort were previously unable to follow health providers’ recommendations on nutrition due to poverty and affordability of fruits and vegetables. Women with children born in 2012–2017 felt that increased household wealth and capacity to purchase assets represented an important driving factor at the distal level. Improved feeding practices, food security, and access to health services were identified as important underlying causes of improvements in stunting by mothers in communities. An FGD participant in the 1992-1997 group highlighted the impact of improved household wealth: *“We have money because of migration, which [money] give us huge assistance.”*

## Discussion

Three decades of democratic, economic, and health system reforms in the Kyrgyz Republic have contributed to improvements in population health and nutrition. The greatest gains in growth were achieved during 1997 to 2005/2006, with limited achievements between 2005/2006 to 2014. Key success factors that created an enabling environment for the reduction of stunting in the Kyrgyz Republic include: *1*) improved economic conditions, reduction of poverty and increased inflows of remittances from international migrants; *2*) introduction of agrarian land reforms; *3*) implementation of a series of health sector reforms that emphasized and increased access to universal primary health care; and *4*) improved food security and nutrition status particularly among women and children.

### Strengths and limitations

This study was the first mixed-method systematic and comprehensive analysis of major determinants of stunting reduction in the Kyrgyz Republic. An evidence- and context-based conceptual framework guided the research and expanded the range of stunting determinants considered and analyzed. Harmonization of all 4 DHS and MICS surveys allowed for analyses across 4 time points and incorporated area-level or ecological variables from robust and reliable data sources. The qualitative component captured diverse, multilevel perspectives, and demonstrated that tangible local changes in socioeconomic status, infrastructure, and knowledge and nutrition behaviors have enabled the stunting decline in the Kyrgyz Republic. Several limitations should be considered for both qualitative and quantitative research approaches. Among national stakeholders, qualitative exploration of policies and programs focused on nutrition-specific policies (e.g., breastfeeding, micronutrient supplementation, etc.), with less emphasis on potential nutrition-sensitive initiatives. Inferences from the ecological variables are susceptible to ecological fallacy, as estimates based on regional-level data may not translate to individual-level effects. Smaller sample sizes of child anthropometry data in the 1997 DHS survey (*n* < 900) and the lack of data available for children aged over 36 mo may have limited significant factors in multivariable models. The decomposition analysis was statistically powerful, however, limitations related to this approach discussed by other published studies apply (72–74).

### Existing evidence

Improvements in stunting were achieved despite several periods of political instability, as well as significant democratic and government changes. The transition to democracy and independence after the collapse of the Soviet Union led to a rapid economic and GDP decline due to decreased production of commodities (e.g., grain and livestock), and was compounded by the Russian financial crises (9, 75). The economy stabilized in 1996 and rapid economic gains were achieved after 2000, with GDP values surpassing precollapse performance by 2009. The implementation and continuity of national development, poverty reduction, and broader health sector reforms and programs represent potential contributions to improvements in population health (76), through sustained political and financial commitments to the provision of social services.

Emigration from the Kyrgyz Republic for employment increased during the early 2000s, as individuals travelled to neighboring countries, largely Russia and Kazakhstan. The majority of migrants sent remittances back to the Kyrgyz Republic, a practice that has contributed to poverty reduction and household investments in the health of children and women (7). Qualitative findings from community key informants and mothers in communities reinforced the importance of remittances to poverty reduction and their improved capacity to purchase food and household assets.

Poverty reduction efforts transformed the costly and poorly targeted social safety net of “privileges” used by the Soviet Union (77). Implementation of the State Benefits Law in 1991 later led to the introduction of 2 cash transfer programs: *1*) the Universal Monthly Benefit (1995), a guaranteed minimum income per household and *2*) the Monthly Social Benefit (1998), a cash income-replacement program targeting disadvantaged and vulnerable populations (e.g., children with disabilities, mothers of large families, and the elderly). These programs represented innovative efforts to provide targeted social protection, however, the coverage, monitoring, and support provided to individuals were limited (77). Other efforts included the 1998 National Poverty Reduction Plan “Araket” (43) and the 2002 Comprehensive Development Program, which prioritized poverty reduction, and introduced multisectoral development efforts (26). The equity analysis demonstrates overall improvements in socioeconomic conditions (e.g., poverty, education, wealth, area of residence), and gaps were reduced across all dimensions, with limited differences after 2005/2006. Population-wide improvements are also evident in the HAZ kernel density plots.

The introduction of a series of agrarian land reforms during the 1990s supported improvements in national and individual wealth. Land reforms after the Soviet Union collapse transferred the use of arable land from primarily State entities to citizens and in 1998 constitutional amendments allowed for private land ownership (27). During the Soviet era, the Kyrgyz Republic's regional crop specializations in livestock and crops for grazing and animal feed (e.g., barley and corn) and the transition to subsistence crops (e.g., wheat and cereals) in the 1990s and early 2000s helped to address issues of high food insecurity (20,78). By 2008, nearly 75% of arable land was transferred to the public and increased agricultural output and land productivity was achieved (20). In addition to representing an important source of both individual and national income (44), the implementation of land reforms improved food security, nutrition, and reduced poverty through increased subsistence farming, and personal and household assets. These notable periods of economic improvement overlap with significant declines in childhood stunting between 1997 and 2005/2006. Literature on the impact of the land reforms also supports findings from the policy and qualitative analysis, indicating associations between increased children's weight and health, as well as significant associations between the number of months children were alive during the land reforms and both HAZ and WAZ (27).

Multiple health sector reforms in the Kyrgyz Republic after the collapse of the Soviet Union increased access to primary health services and reduced financial barriers to care. Soviet investments in the Kyrgyz Republic in education and health focused on universal and egalitarian coverage of services and represented a foundation and opportunity for health system reforms (76). The Semashko model of health was established in the 1920s in former Soviet nations and improved health, access to education, access to water and sanitation, and coverage of health services including childhood immunizations (79). Free and universally accessible health services were delivered through a centralized and hierarchical system, delivered primarily by specialized health providers and centers. However, this approach to health care was fragmented, expensive, and inequitably distributed (80). A supportive policy environment and the introduction of comprehensive, multifaceted and multilevel health sector reforms and policy efforts improved the cost-effectiveness of the health system, and the accessibility, affordability, and quality of health services (80). Top-down reforms transformed health financing and increased the financial protection, efficiency, and transparency of the health system (18, 22), whereas bottom-up approaches improved the local coverage and provision of health services, particularly for rural and hard-to-reach populations in the Kyrgyz Republic (22, 54). Evidence on improvements in maternal, newborn, and child health and universal health coverage and primary care (18, 76, 81) highlight the enabling role of the health sector reforms to improve the equity, quality, and efficiency of health services in the Kyrgyz Republic. Breastfeeding promotion represented an important area of nutrition-specific policies and programs (42, 44–47). However, despite efforts by both government and donors, exclusive breastfeeding rates remain low (29, 34) and represent an area of future effort and intervention.

The Victora curve analysis indicates that notable improvements in stunting were achieved among children under the age of 6 mo, and that key drivers of this change may include improved maternal nutrition, increased intrauterine growth, increased weight at birth, and improved breastfeeding. Similarly, the linear multivariable regression model findings indicate that poverty reduction and increased maternal caloric intake have substantially improved HAZ among children. Food insecurity improved during the 1990s, but may have declined during periods of political unrest and was related to slowed declines in stunting after 2005/2006 (36, 82). Literature on food security and availability in the Kyrgyz Republic also supports these findings, as it contributes to chronic malnutrition, anemia, and other micronutrient deficiencies among children (34). Further, regional rates of food insecurity are aligned with oblasts facing the highest burden of stunting (9).

### Remaining challenges

Despite national improvements in stunting, underweight, and wasting for children aged under 3 y and under 5 y, regional disparities persist and suggest that targeted interventions may be necessary to reduce stunting and wasting in the Kyrgyz Republic. Maternal nutrition indicators (e.g., maternal height, anemia, and underweight prevalence rates) did not improve substantially over the study period and may represent an area of future research and policy efforts in order to ensure continued improvements in nutrition.

## Conclusion

National government and donor efforts to implement nutrition-specific and -sensitive efforts after the collapse of the Soviet Union have contributed to substantial improvements in stunting among children in the Kyrgyz Republic. Improvements in linear growth were achieved amidst significant political changes, as well as periods of civil unrest and economic decline. Economic growth and increased labor migration represented key enablers for poverty reduction and improved food security. The introduction of land and health sector reforms also represents notable developments in the nutrition transition. The Kyrgyz Republic represents a stunting case study exemplar with multilevel enablers and factors that have contributed to dramatic improvements in health, nutrition, and stunting of children over time. Understanding and exploring progress and prospects for improvement are critical to ensuring the sustainability of improvements and dissemination of lessons learned to other countries in Central Asia and globally.

## Supplementary Material

nqaa120_Supplemental_Material

## Notes

This study was funded by a grant to the Centre for Global Child Health from Gates Ventures. The funder had no role in the design, implementation, analysis, or interpretation of the data.

Supplementary Appendices 1–7 and Supplemental Systematic Scoping Literature Review Data are available from the “Supplementary data” link in the online posting of the article and from the same link in the online table of contents at https://academic.oup.com/ajcn/.

SB to TV authors organized alphabetically.

Data described in the manuscript, code book, and analytic code will be made available upon request pending application and approval.

Published in a supplement to the American Journal of Clinical Nutrition. The Guest Editor for this supplement was Mark Manary, and has no disclosures. Publication costs for this supplement were defrayed in part by the payment of page charges by Gates Ventures. The opinions expressed in this publication are those of the authors and are not attributable to the sponsors or the publisher, Editor, or Editorial Board of the American Journal of Clinical Nutrition. The Supplement Coordinator for the supplement publication was Nadia Akseer, Gates Ventures/Hospital for Sick Children, Toronto, Canada. Supplement Coordinator disclosure: no conflicts to disclose. The Stunting Exemplars research Principal Investigator was Zulfiqar A Bhutta, Hospital for Sick Children, Toronto, Canada. Principal Investigator disclosure: no conflicts to disclose.

Abbreviations used: CAGR, compound annual growth rate; CIX, Concentration Index; DHS, Demographic and Health Survey; DID, difference-in-difference; FGD, focus group discussion; GDP, gross domestic product; HAZ, height-for-age z-score; IMCI, integrated management of childhood illness; MICS, Multiple Indicator Cluster Survey; PPP, purchasing power parity; SII, Slope Index of Inequality; VHC, village health committee; WAZ, weight-for-age z-score.
