# Supplementary material for: Drivers of stunting reduction in the Kyrgyz Republic: A country case study
Source: Am J Clin Nutr. 2020 Jul 16;112(Suppl 2):830S–843S. doi: 10.1093/ajcn/nqaa120 (PMC7487426; doi:10.1093/ajcn/nqaa120)
Supplement: nqaa120_Supplemental_Material [file nqaa120_supplemental_material.zip › Kyrg Stunting Case Study Online Appendices May182020.docx]

**Drivers of stunting reduction in Kyrgyz Republic: A country case study**

**Authors:** Jannah M Wigle, Dr. Nadia Akseer, Dr. Roman Mogilevskii, Samanpreet Brar, Kaitlin Conway, Zalina Enikeeva, Mariia Iamshchikova, Muhammad Islam, Dilbara Kirbasheva, Aviva I Rappaport, Hana Tasic, Tyler Vaivada, Dr. Zulfiqar A. Bhutta

**Online Supplementary Materials**

**List of Appendices**

[Supplementary Appendix 1 Descriptive Analysis of Contextual Factors 3](#_Toc34742691)

[Supplementary Figure 1A Trends in GDP per capita, poverty and urbanization trends, 1990- 2017 3](#_Toc34742692)

[Supplementary Figure 1B Trends in contextual indicators, 1995-2017 3](#_Toc34742693)

[Supplementary Appendix 2 Systematic Scoping Literature Review 4](#_Toc34742694)

[Supplementary Figure 2 Literature review flow diagram 5](#_Toc34742695)

[Supplementary Appendix 3 Multivariable Analyses Methods 11](#_Toc34742696)

[Supplementary Appendix 4 Qualitative Data Collection and Analyses Methods 14](#_Toc34742697)

[Supplementary Table 1 Inclusion Criteria 15](#_Toc34742698)

[Supplementary Appendix 5 Quantitative Results 18](#_Toc34742699)

[Supplementary Figure 3A Spline analysis of inflection points of change in the slope of HAZ, 1997 18](#_Toc34742700)

[Supplementary Figure 3B Spline analysis of inflection points of change in the slope of HAZ, 2014 18](#_Toc34742701)

[Supplementary Figure 4A 1997 stunting prevalence by region 19](#_Toc34742702)

[Supplementary Figure 4B 2005 stunting prevalence by region 19](#_Toc34742703)

[Supplementary Figure 4C 2012 stunting prevalence by region 19](#_Toc34742704)

[Supplementary Figure 5A Change in absolute SII by year in Kyrgyz Republic 20](#_Toc34742705)

[Supplementary Figure 5B Change in absolute CIX by year in Kyrgyz Republic 20](#_Toc34742706)

[Supplementary Figure 6 Stunting prevalence by gender, 1997 – 2014 21](#_Toc34742707)

[Supplementary Table 2 Descriptive trends in stunting determinants in 1997 – 2012 in children <36 months 21](#_Toc34742708)

[Supplementary Table 3 Decomposition analysis for children among <36 months from 1997-2012 26](#_Toc34742709)

[Supplementary Figure 7 Decomposing predicted changes in HAZ among children <36 months (i.e. relative ranking of product coefficients for determinant domains) from 1997-2012 26](#_Toc34742710)

[Supplementary Table 4 Decomposition analysis for children among 6-23 months from 1997-2012 27](#_Toc34742711)

[Supplementary Figure 8 Decomposing predicted changes in HAZ among children 6-23 months (i.e. relative ranking of product coefficients for determinant domains) from 1997-2012 27](#_Toc34742712)

[Supplementary Table 5 Difference-in-differences multivariable regression for children under 36 months years from 1997 – 2012 28](#_Toc34742713)

[Supplementary Table 6 Difference-in-differences multivariable regression for children aged 6-23 months from 1997 – 2012 32](#_Toc34742714)

[Supplementary Table 7 Difference-in-differences multivariable regression for children aged 2-5 years old from MICS surveys 2005/6 – 2014 38](#_Toc34742715)

[Supplementary Appendix 6 Programs and Policies 43](#_Toc34742716)

[Supplementary Table 8 Detailed timeline of nutrition-specific and -sensitive laws, policies and programs in Kyrgyz Republic (1990-2017) 45](#_Toc34742717)

[Supplementary Appendix 7 Qualitative Results 60](#_Toc34742718)

[Supplementary Table 9: Summary of in-depth interview national respondents 61](#_Toc34742719)

[Supplementary Table 10 Summary of in-depth interviews with community stakeholders 87](#_Toc34742720)

[Supplementary Table 11 Description of food children consume in kindergartens in oblasts 92](#_Toc34742721)

[Supplementary Table 12 Demographic characteristics of FGD participants 95](#_Toc34742722)

[Supplementary Table 13 Comparison of menu for children born in 1990s and 2010s 101](#_Toc34742723)

[Appendix References 103](#_Toc34742724)

# Supplementary Appendix 1 Descriptive Analysis of Contextual Factors

## Supplementary Figure 1A Trends in GDP per capita, poverty and urbanization trends, 1990- 2017


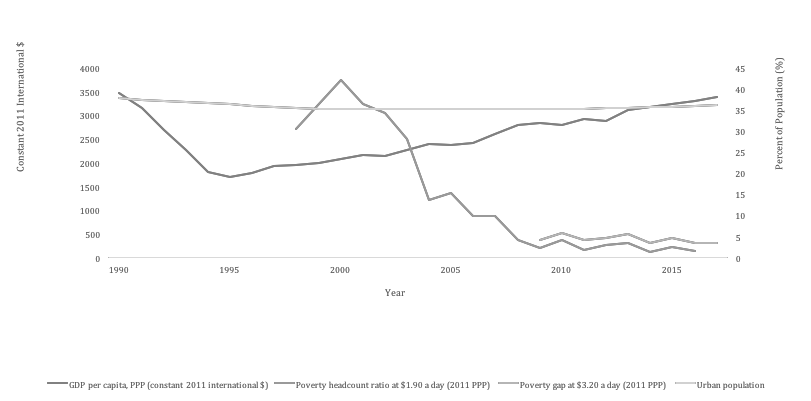


*Source:* (1)

## Supplementary Figure 1B Trends in contextual indicators, 1995-2017


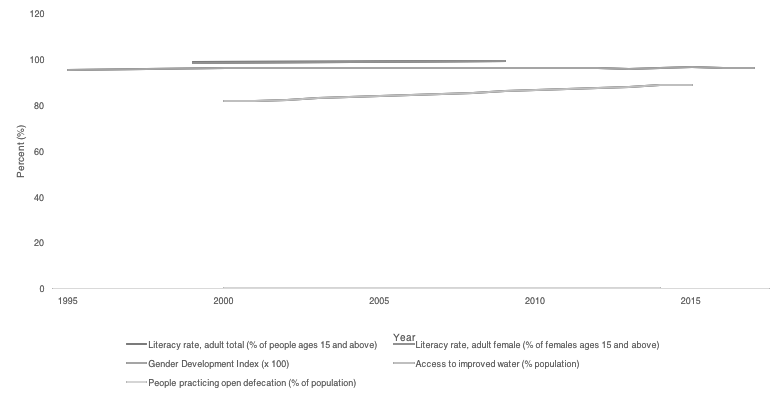


*Source:* (2)

# Supplementary Appendix 2 Systematic Scoping Literature Review

Search terms employed include “stunting” or “linear growth” or “linear growth stunting” or “HAZ” or “height or “height-for-age” or “LAZ” or “length” or “length-for-age” or “undernutrition” or “malnutrition” or “nutr*” or “health” and “child*” or “infan*” and “Kyrgyz Republic” or “Kyrgyzstan” or “Kirghiz S.S.R.” or “Kirghiz Soviet Socialist Republic” or “Kirghizia” or “Kyrgyz*”. A search for indexed literature was conducted using 15 online databases including: MEDLINE, Embase, AMED, CAB Abstracts, CINAHL, Cochrane CENTRAL, Campbell Collaboration, EPPI Centre Trials Register (TRoPHI), 3ie, JOLIS, African Journals Online, WHOLIS, LILACS, Scopus, and Web of Science. Grey literature search was conducted using Google, a hand search of reference lists of relevant reviews, and direct searching organizational websites, including: national, regional and headquarter websites for UNICEF, WHO,  UNDP, WFP, FAO, World Bank Group Open Knowledge Repository, Asian Development Bank, Global Alliance for Improved Nutrition, International Food Policy Research Institute, Government of Kyrgyz Republic including the Ministry of Health, Ministry of Agriculture and Land Reclamation and the National Statistic Committee.

The exported set of records were de-duplicated and screened for relevance. Records were included if they met all of the following inclusion criteria:

1. included an under-5 population in Kyrgyzstan;
2. published between 1990-2017;
3. examined one or more of the determinants of chronic undernutrition (e.g. determinants, risk factors, policies, programs, interventions, or initiatives); and
4. examined effects on child growth or a reduction in stunting

Published peer-reviewed literature were searched in more than 15 online databases and double-screened against inclusion criteria and manual search of organization websites by multiple reviewers was also conducted to identify relevant grey literature sources. An initial search of databases found 592 records, after removing duplicates and screening according to titles and abstracts, a total of 127 records were included in our analysis, including health policy reform documents (n=63), quantitative analysis of health outcomes (n=2) and nutrition (n=5) and grey literature documents (n=57) (**Supplementary Figure 2).**

## Supplementary Figure 2 Literature review flow diagram

**
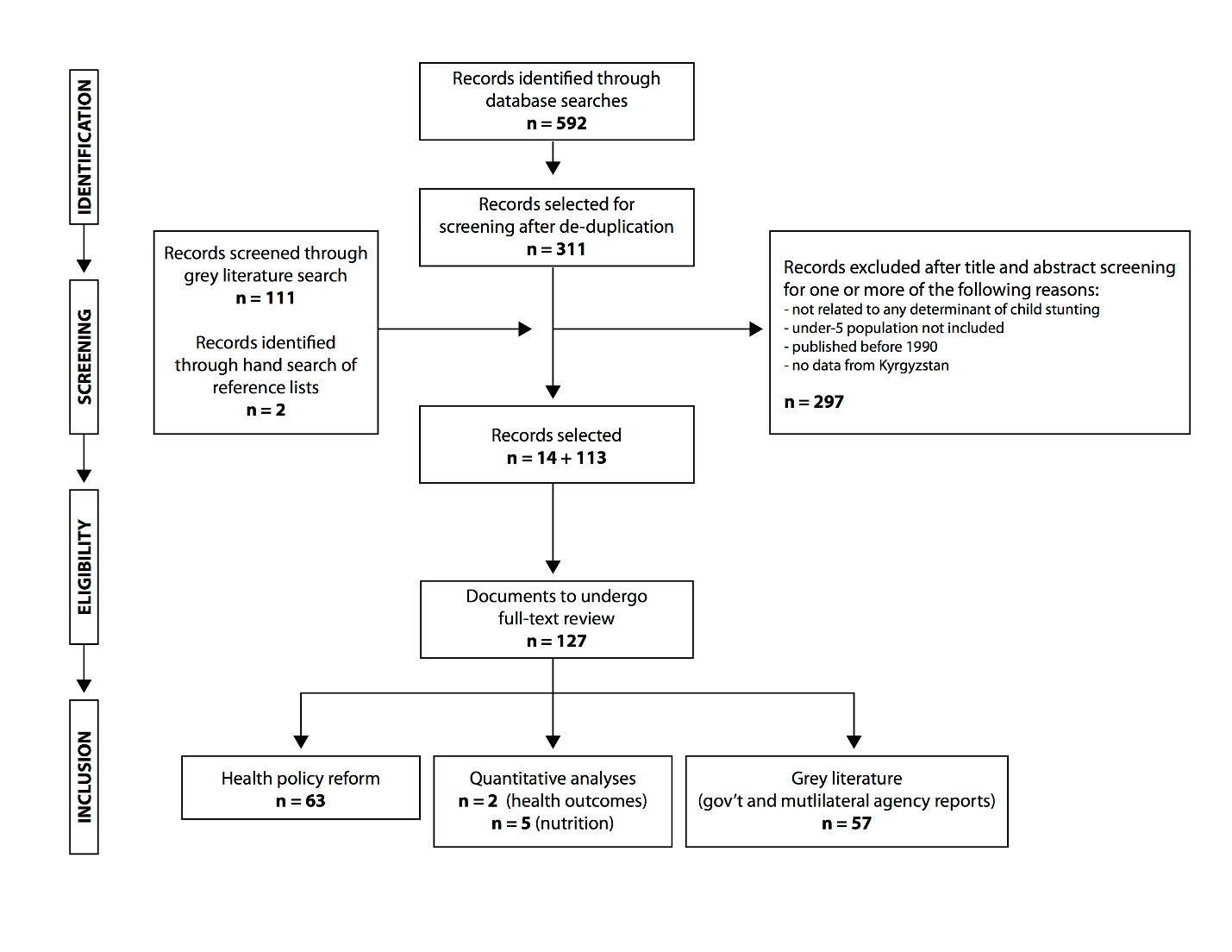
**

**Executive Summary**

The systematic scoping literature review for Kyrgyzstan identified many of the basic causes that resulted in the decline in stunting. This began with the collapse of the Soviet Union and transition to democracy (3–6). Overall, there were improvements in other basic causes including GDP growth (7–10), increased spending on social services (11–15), the development of land reform laws (11,16), higher agricultural outputs (16,17), seasonal labour migration (18), and improvements in primary health care through programs, such as the Manas initiatives (19,20). Periods of economic and agricultural growth coincided with stunting reductions at a population level (21). The levels of poverty declined from 32.2% in the late 1990s to 3.5% in 2017 (2011 PPP) (15). Similarly, Kyrgyzstan’s economy (GDP/capita) improved rapidly between 2000 and 2006 (22). These improvements may be due to national cash transfer programs, poverty reduction frameworks (23) and land privatization. Land privatization was associated with 13% of the variation in HAZ (24). Increased GDP at the national level resulted in increased total health expenditure from 2000-2014, from 4.7% to 6.5% of GDP, leading to decreased out-of-pocket health expenditure and increased health service utilization (15,20,25).

Important underlying causes of stunting include access to safe water (26,27), maternal education (28), and food security and availability. National food availability of grains, livestock and poultry, milk and eggs increased over the period of stunting reduction (17). Improvements in dietary intake were observed between 1999 and 2015 where there was an improvement in mean energy supply (kcal) going from 107% to 120% (29). The percentage of the population suffering from energy inadequacy declined from 16% in 1992 to 6% in 2014. This may have contributed to the declining prevalence of undernutrition from 16.3% in 1999-2001 to 6.4% in 2014-2016 (29).

The immediate causes of stunting in Kyrgyzstan are childhood infection (7,28,30,31), maternal age and height (32), dietary intake (33,34), and breastfeeding (35–37). Therefore, implementation of nutrition specific programs at a national level may have contributed to decreased rates of stunting. Health reforms, such as the *Healthy Nation State Plan* (38–40)*, Healthy Children at the Level of Primary Health Care*  (41,42)*, Manas* (15,20,43,44)*, Manas Taalimi* (5,12,20,43–45),  *Den Sooluk* (13,44,46,47), and the *Integrated Management of Childhood Illness* (IMCI) program (48) all included breastfeeding promotion and infection reduction strategies (39,41,43,47,48). In addition, the national vitamin A supplementation program may have indirectly led to a decline in stunting by decreasing the prevalence of infection (28,49–51).

**Full Literature Review**

Factors that have contributed to a reduction in stunting in Kyrgyz Republic are presented in relation to an adapted version of UNICEF’s conceptual framework for malnutrition (52). In Kyrgyz Republic, barriers such as political unrest, food insecurity, and environmental shocks have contributed to chronic malnutrition among children. Many of the national programs are aimed to reduce the prevalence of micronutrient deficiencies, improve breastfeeding rates, and reduce food insecurity which are unlikely to reduce stunting on their own. However, there were key events and policies such as the agrarian land reform, health system reform, migration and remittances and the cash transfer program for the poor that target the underlying and basic causes of malnutrition. Together, these programs coupled with the underlying contextual factors created an environment that led to the reduction of stunting in Kyrgyz Republic. For example, poverty rates decreased from 1999 to 2008 from 64% to 32%, respectively due to land distribution, lower tariffs leading to cheaper food imports, social security measures, and migration (53). The aim of this literature review is to summarize data from programs, policies, and intervention studies that relate to the causes of chronic malnutrition in Kyrgyz Republic.

**Basic causes**

Kyrgyz Republic gained independence in 1991 following the collapse of the Soviet Union (3–6). At the time of the collapse, the parliamentary system and government structure were already in place in Kyrgyz Republic. However, Kyrgyz Republic needed to learn how to function independently without financial support. The few years following the collapse resulted in a rapid decline in the production of commodities such as grain, livestock, and GDP in Kyrgyz Republic. This is partly due to the Russian financial crisis (5,53). In 1996 the decline in GDP became stable, more rapid increases in GDP were seen following 2000, and by 2009 GDP was at a value higher than it was in 1990 (7–10,20). Increasing GDP from 2000 onwards in Kyrgyz Republic led to increased spending on social service financing such as health, social welfare and insurance (11). For example, the percent of GDP spent on education, health, and social welfare increased between 2008-2012 (11). Spending increased from 2.4% to 3.7% of total GDP on health and from 2.5% to 5.7% on social welfare and insurance (11). The increases in GDP led to marked changes in development as new policies and programs were put into place during these years (9).

An important contribution to improved economic growth was the agrarian land reforms. In Kyrgyz Republic 85% of the agricultural land is composed of pastures for grazing animals while 15% of the land is arable for crop growth (16). Following the collapse of the Soviet Union, was the initiation of the laws of the land reform. Until this time, agricultural land in Kyrgyz Republic was owned by large enterprises. These 500 enterprises controlled 98% of the arable land in Kyrgyz Republic (16). During the mid-1990s, the Government of Kyrgyz Republic rapidly distributed the agricultural land to individuals. The pasture land remained state property, however the land reform legislation aimed to make 75% of the arable land private (16). In 1998, a constitutional amendment was made which allowed for private land ownership which all land use certificates transformed into land ownership certificates (24). In total, 940,000 hectares of land was distributed as land shares to approximately 2 million landowners which accounted for 2/3 of the population (16). By 2008, the individual sector controlled 75% of the arable land in Kyrgyz Republic (16).

This was an important and successful measure for the reduction of poverty. In part because the result was farmers had access to land which gave them control over their own food security, and also due to improved assets (24). Children exposed to land privatization for longer durations were significantly taller and heavier, than children who were unexposed (24). Data from the Life in Kyrgyz Republic Survey showed that the number of months a child was alive during the land reform was significantly associated was HAZ and WAZ p<0.01 (24). Furthermore, being exposed to a land reform explains 13% of the variation in HAZ and 8% of the variation in WAZ (24).

In addition, this led to a higher agricultural output at the national level as individual farms have greater output than agricultural enterprises (16). Data suggests that individual farms consistently have higher levels of land productivity (16). This was evident in Kyrgyz Republic. For example, prior to the collapse of the Soviet Union the individual farms contributed to 45% of the gross agricultural output and the enterprises produced 55% of the output. By 2008, individual farms produced 98% of the agricultural output and enterprises produced just 2% of the gross agricultural output (16).

Similarly, in 2002, agriculture accounted for 36% of GDP and almost 50% of employment in Kyrgyz Republic (54). These years of economic and agricultural growth coincide with stunting reductions at a population level (21). The progress in GDP growth began to slow in 2008-2009 due to various political and environmental shocks, which resultantly may have caused stagnant rates of linear growth and stunting reduction (55).

An important contribution to an improved economy in Kyrgyz Republic was labor migration and remittances. In the early 2000s, seasonal labour migration began where citizens would leave to neighbouring countries to seek employment and better living conditions (18). A majority of the migrants are male and 80% moved to Russia for employment, the second most common place for migration was Kazakhstan (18). Data suggests that 80% of migrants send remittances back to Kyrgyz Republic (18). Data from Tajikistan and Kyrgyz Republic show that poverty was significantly reduced by remittances (18).

In terms of political shocks, the first event occurred in March 2005, when the president was ousted for corruption during riots, this event is known as the tulip revolution (5,9,18,46,55). The second event, took place in 2010, where the succeeding president was ousted by protestors, for political corruption (45,56). This event, led to an outbreak of violence and civil unrest between the Kyrgyz and Uzbeks in Osh, Jalalabad, and Batken; all southern oblasts (31). The violence led to displacement and mortality (45,55). In addition, this political unrest led to increasing issues of food insecurity (30,57). This event was beneficial in the long term because it led to the establishment of a parliamentary system in Kyrgyz Republic (11,58).

In terms of environmental shocks, Kyrgyz Republic is susceptible to unpredictable changes as it is seismically active and mountainous which can lead to avalanches, landslides, and mudslides. In addition, Kyrgyz Republic is susceptible to droughts and floods, which causes food insecurity, an underlying cause of malnutrition which will be discussed in the forthcoming section (45). In addition, extreme weather is becoming more common. Droughts are increasing in frequency globally, and in Kyrgyz Republic (59,60). A drought in 2007, led to lower crop production, food shortages, and resultantly a higher cost of food (5,30,55). Drought and heavy snowfall have led to a reduction in potatoes and vegetables since 2006 (61). Contrary, the extreme frost in 2008 (5,59,62), resulted in increased flooding (5,45,61,62) and led to volatile food prices, and increased food insecurity (63). These conditions can lead to chronic malnutrition among children, which may explain the stagnant stunting trends between 2005 and 2012 (64,65).

**Underlying causes**

Important underlying causes of stunting include access to safe water, maternal education, and food security. In Kyrgyz Republic, in 2001, 17% of the population didn’t have access to safe drinking water (66). In 2010, UNICEF supported widespread access to safe water, following the violence in Kyrgyz Republic (67). This reduced the inaccessibility to safe drinking water to 12% in 2006 (45,59,62) but this increased slightly to 15% in 2014 (45,59,62). Regional disparities exist for the accessibility to safe water sources (46). For example, access to safe water is more problematic in rural areas compared to urban ones; 20% of the population living in these rural areas lack safe water access (62). However, hygiene practices after defecation were utilized widely in Jalalabad, Uzgen, and Jumgal at 98%, 95%, and 79%, respectively, while fewer people reported hand washing after other critical points (28).

Another important determinant for chronic malnutrition in children is maternal education. Women in Kyrgyz Republic are well educated, almost 100% of women in a baseline survey in Uzbek, Jalalabad, and Jumgal were educated, and only a small portion had complete secondary school (28). Research in these Kyrgyz Republic oblasts depicted that higher household assets coupled with higher maternal education resulted in better care practices (28). Along these lines, Mothers with poor education had worse care practices (68). Another study showed that antenatal visits are 17% higher in educated women compared to other less educated women (7). However, despite this, nearly all mothers in Kyrgyz Republic received at least 1 antenatal care visit during their last pregnancy (7).

Food availability is not a consistently an issue in Kyrgyz Republic as data shows that there are enough calories available per capita (34). For example, data from 2014/16 estimates that there are 2800 kcal/person/day available (34). Research shows that food insecurity in Kyrgyz Republic is season dependent, with improved consumption during the summer months, and poor consumption in winter and early spring (5,30,31). This is not surprising, as described above, environmental and climatic shocks are a basic cause of malnutrition in Kyrgyz Republic. Food insecurity was higher during political unrest. Food security and fell by 44% in 1990 – 1993 and then continued to drop by another 5% in 1995 (69). However, from 2008-2010, about 20% of households in Kyrgyz Republic were severely food insecure, and 13% were moderately food insecure (5). This may explain the slower decline in stunting rates following 2010 (33,65).

Stunting is highest in regions where food insecurity is higher (55,62). Food insecurity, is more prevalent in rural areas and was highest in Talas Oblast (31). Price differences between oblasts and urban/rural areas are substantial, for instance in Bishkek the cost of a comprehensive food basket is 1.17 times higher than the average national cost of the same food basket, while in rural Naryn and Talas the ratio falls to 0.91. At the same time it is necessary to mention that food basket in Osh and Batken oblasts is by 8% higher than the average national cost of the same food basket, when in 1998 this indicator was lower by 8%.-9% (70).

**Immediate causes**

A low height for age can indicate a history of illness and infection (71). In Kyrgyz Republic, childhood infection is a known cause of malnutrition (31). Vitamin A deficiency, which was prevalent in ~32% of infants can lead to increased susceptibility to respiratory infections and measles (27,52). Consumption of vitamin A rich food is low in Kyrgyz Republic, particularly in Jumgal where less than 9% of children consumed these foods daily. In other regions such as Uzgen and Jalalabad 33% of infants consumed these foods daily (28). However, to improve intake, there was a largescale vitamin A supplementation program between 2005 – 2011 that achieved 97% coverage (28,49,50).

A primary cause of infection in Kyrgyz Republic is diarrhea; evidence shows that large deworming campaigns are not widespread, in 2014, in Jalalabad, Jumgal, and Uzgen only 15-20% of children had received de worming. Similarly only 5-12% of women received deworming during antenatal care visits (28). This is problematic as data suggests that there is an extremely high prevalence of soil transmitted helminth infection (27,45). For example, 75% of school aged children, and 50% of the population are affected (27,45). Since 2007, the rate of intestinal infection decreased (59). The reduction in diarrhea may be attributed to the health sector reform programme (Manas). Manas focused on improvement of care at the primary health level for six diseases including acute respiratory infections (ARI) and diarrheal diseases (20). This is important because ARI are the second leading cause of death in children under 5 in Kyrgyz Republic (62).

The rate of exclusive breastfeeding has increased from 32% to 41% from 2006 – 2014 (72). The rate of exclusive BF in Kyrgyz Republic is surprisingly low (28,31). Similarly, within the first month of birth 11.3% of infants were given liquids other than breastmilk, and only 50% of infants were breastfed within the first hour of birth (72). As such, in 1996, UNICEF and the WHO began an advocacy campaign supporting breastfeeding which led to the government’s adoption of early initiation of breastfeeding, and exclusive breastfeeding until 4 – 6 months of age.

Adequate maternal nutrition is important for ensuring adequate fetal growth and development (28). In Kyrgyz Republic, maternal energy intake seems to be adequate with approximately 55% of women of reproductive weight have a normal BMI (18.5 - <25) and just 3% of WRA are underweight (BMI<18.5) (33). Low birthweight is a result of poor nutrition and environment in utero (27). In Kyrgyz Republic, 5% of newborns had low birth weight (<2500 g) (5,18,27,73). This data varies by geographic region, Osh reported the highest prevalence of LBW babies at 10% (27). This data is in line with maternal BMI data. Low birth weight rates ranged from 4-10%, they are higher among uneducated mothers, mothers living in rural areas, and poorer households (5).

Between 1992 and 2014 the percentage of the population suffering from energy inadequacy declined from 16% to 6%, respectively (34). These data align with the stunting decline from 1995 – 2014 (64,65,74). Food insecurity in Kyrgyz Republic varies with season, and is worse in the winter and early spring once the summer harvest has deteriorated and food prices have increased (31). There were more food insecure households in 2007 (48%) than there were in 2006 (38%) (62). Similarly, the 2008 global food crisis was apparent in Kyrgyz Republic, as they experienced an extreme winter with severe hailstorms followed by power outages and a locust infestation. The total agricultural production declined by 1.2% despite having more cultivated land available, the result was 20% of households having inadequate energy and protein intake (27). In both years the poorest wealth quintiles had lower caloric intake than the higher wealth quintiles (62). Emergency Food Security Assessments in Kyrgyz Republic have shown that poorer households consume high quantities of nutrient poor starch foods (31). In 2011, 12% of households in rural areas had a poor or borderline diet compared to 9% of households in urban areas (31).

Food insecurity is a contributing factor to chronic malnutrition, anemia, and other micronutrient deficiencies among children in Kyrgyz Republic (31). A recent survey showed poor dietary diversity among children aged 6-23 months, only 45% of children in southern Oblasts ate foods from at least four food groups in the past 24 hours (28). Contrary, a follow-up survey showed that 87% of infants consumed a diet with the minimum diet diversity, and 75% had a diet with the minimum recommended meal frequency (71). Other data from Kyrgyz Republic shows 16% of children of this age met the minimum acceptable diet (8,73). However, only 23% of infants 6-23 months for age met the minimum acceptable diet (an indicator based on the minimum frequency of feeding and minimum dietary diversity) for their age and breastfeeding status (28). There are programs in place, such as Gulazyk which aims to educate women about the importance of adequate nutrition during pregnancy, exclusive breastfeeding and improved complementary feeding (75). However, these programs likely did not have any impact on stunting reduction.

# Supplementary Appendix 3 Multivariable Analyses Methods

We undertook two sets of hierarchical multivariable analyses that are discussed in detail below. Using complementary approaches, each of these analyses attempts to answer the same research question i.e. what are the main predictors of change in child linear growth in Kyrgyz Republic during critical time periods? The linear regression based on panel datasets uses a difference-in-difference analysis framework where time*covariable interactions are used to assess factors impacting HAZ decline. This allows the analysis of multiple years of survey data and adjusts for baseline levels of covariables and varying hypothesized growth trajectories through the interaction term. The Oaxaca-Blinder decomposition is based on the same set of individual/household level data (with ecological variables). However, by design, the decomposition only uses two survey time points in a given analysis and thus “ignores” in-between survey rounds and any intermittent fluctuations in the predictors. As has been suggested in previous decomposition analyses, we operationalize child HAZ as the linear growth outcome due to its greater statistical efficiency relative to the dichotomous child stunting variable. Each of the two multivariable regression-based analysis methods pose their own strengths and limitations – however, as sensitivity analyses, study inferences should be anchored in both and congruent findings between the methods strengthen the key messages.

**Linear Multivariable Regression (Difference-in-Difference Analysis)**

We undertook linear multivariable regression analyses, and included all covariables and adjustment factors as fixed effects. We added interaction terms between each potential determinant and time (i.e. time*covariable interaction terms), which signify whether a change in a proposed predictor of HAZ leads to a change in HAZ over the studied time period. The four cross-sectional surveys used in this analysis were assembled into panel datasets, and difference-in-difference (DID) analyses were used. Univariate statistics were estimated using means/standard deviations and frequencies/proportions as appropriate. We used the interaction estimators in unadjusted and adjusted regression methods to estimate the DID effect. The general model specification included an interaction term between time and the various indicators. The multivariable regression models were adjusted for child age, sex and region. Effect estimates were reported with 95% confidence intervals. All statistical analyses was performed using Stata version 14.0. The complex sampling design of DHS surveys was taken into account by using the STATA's svyset function. Standard errors were estimated using the Taylor series linearization method, which incorporates sampling weight, primary sampling unit, and stratum appropriate to the DHS sample design. 

To examine the association between HAZ and various indicators, we conducted a series of step-wise linear regression models. A hierarchical modelling approach using distal, intermediate and proximal level variables was executed as suggested by Victora 1997 (76) to generate the final multivariable models. Variables within each level were selected from our general conceptual framework as defined in Figure 2 in the main paper. Step 1 was a series of bivariate regressions to determine crude associations between indicators in our conceptual framework and HAZ outcome. Step 2 was to use all candidate variables for multivariable model building (i.e. with p-value ≤0.20) irrespective of their direction to move forward for multivariable modeling. Selected variables are entered into backward stepwise elimination modeling within their respective levels and those with p-values <0.15 are retained. At each step, the crude and adjusted associations between the indicator and HAZ was analyzed for statistical significance. Multicollinearity among adjustment variables was evaluated using variance inflation factors (VIF) where VIF>3 were considered suspect for collinearity.

**Oaxaca-Blinder Decomposition**

We also undertook the commonly used Oaxaca-Blinder decomposition methods (77,78) to assess determinants of nutritional change over time in Kyrgyz Republic. These methods based on individual-level data have high statistical power and have been widely used to assess nutrition determinants in low and middle income settings (78–81).

We analyzed individual-level data from two rounds of Kyrgyz Republic’s DHSs: 1997 and 2012. Our analysis focused on the index mother-child pair from each DHS round. Defined as the youngest child of the youngest mother in each household, selection of an index pair simplifies the model and interpretation, and is common practice in advanced analysis of DHS datasets. The total number of index pairs available from each survey were n=828, n=2,096 for DHS 1997 and 2012, respectively. A flow chart outlining sample size breakdown during the index pair selection process is presented in the main paper. Given that the dietary needs/practices and growth trajectories of children in the first 1000 days of life vary notably from children beyond 2 years of age, it has been suggested that these two cohorts be analyzed separately to unmask true effects of environmental conditions and other factors on undernutrition. We conducted analyses for the entire under-36 month child population, and the 6-23 month population, as a result of data availability (82).

We used the continuous formulation of HAZ (as opposed to categorical stunting) as the dependent outcome to strengthen statistical power of the analyses. Linear least square regression models -accounting for survey design and weights - were used to assess associations between $\boldsymbol{y}_{\boldsymbol{i,t}}$, our outcome variable measured for a child *i* at time *t*, a vector of time-varying determinants (**X**), time-invariant child age and sex control variables (**C**), and a survey round time variable (**T**) to capture any trend effects. Collectively, with the standard error term, the model is expressed in Equation 1.

$\boldsymbol{Y}_{\boldsymbol{i,t}}=\boldsymbol{\beta}\boldsymbol{X}_{i,k}+\boldsymbol{C}_{i}+\boldsymbol{T} + \varepsilon_{i,t}$ [Equation 1]

Relevant individual and household level determinants in the DHS dataset were supplemented with oblast-level variables from the ecological dataset. When similar variables were available in both the ecological and DHS datasets, the latter were selected for analysis. The conceptual framework and corresponding list of covariables, their data sources, and definitions used in decomposition assessment are included in Appendix 5 and 6. Applying the conceptual framework, we used a similar hierarchical modelling approach (as described for DID analysis) whereby we examined the distal, intermediate, and proximal level determinants of HAZ.

Equation 1 was applied to derive β coefficients for determinants (DHS 1997 – DHS 2012). To explain the relative contribution of each covariable over time to HAZ change, we used the Oaxaca- Blinder decomposition under the assumption that the β coefficients are the same across the two populations and the error term has the mean zero. Using the estimated parameters from Equation 1 and the (weighted) means of explanatory variables in the two time points, we applied Equation 2 (e.g. for years 1997 to 2012) to obtain the predicted change in HAZ due to the change in each determinant (83).

$$\Delta\bar{Y}_{i,t}=\beta\left( \bar{X}_{2012}-\bar{X}_{1997} \right)$$

The product coefficients for individual determinants were subsequently ranked to identify the relative contribution of each factor to HAZ change. Like determinants were also grouped into broader domains for interpretation. We examined variance inflation factors (VIF) to assess multicollinearity between variables whereby a VIF > 3 was considered suspect of high inter-variable correlation. For model building, a p-value <0.20 was considered statistically important and variables with p< 0.15 were retained in the final hierarchical multivariable models. All analyses were carried out in Stata version 14.0.

# Supplementary Appendix 4 Qualitative Data Collection and Analyses Methods

The qualitative component of the case study aimed to understand the determinants of stunting reduction among children in Kyrgyzstan through exploring the perspectives of key national stakeholders in the development and implementation of relevant policies and programs, and through understanding the experiences of child care workers and mothers in the community. Specific qualitative research objectives included:

- To identify the nutrition-specific and -sensitive key events (policies/strategies/laws/legislations and programs) in Kyrgyzstan that may have contributed to a reduction in child stunting;
- To understand the main success factors and challenges of relevant nutrition-specific and –sensitive facilitators key events (policies/strategies/laws/legislations and programs) in Kyrgyzstan
- To identify important contextual factors that have functioned as enablers or drivers of national-level stunting change in Kyrgyzstan;
- To document community-level perspectives and experiences on the stunting transition in Kyrgyzstan by consulting mothers of young children and child care workers

The conceptual framework by Black et al., informed the development of an adapted framework (Figure 2 in main paper), the design of the in-depth interview and FGD guides, as well as analysis and interpretation of the qualitative data. Data was analyzed using key themes including: basic causes, underlying causes, and immediate causes of reduction in stunting and malnutrition.

**Research Design**

We undertook three independent research activities to inform study objectives. At the first stage, national stakeholders were interviewed to provide insight and expertise on objectives 1, 2 and 3. This top-down approach aimed to solicit macro-level perspectives and experiences in health and nutrition in Kyrgyzstan. To understand how individuals in the community received and implemented major nutrition-specific and –sensitive policy/program events and their experiences in the nutritional transition as a whole, we consulted childcare workers in the community (e.g. at schools, health facilities, etc.) and the mothers of these children. These latter two research activities largely informed objectives 3 and 4, but also shed light on objective 2.

*Sampling and Recruitment Strategy*

Participants were identified and selected using purposive sampling strategies (84), including snowballing sampling (85). Stakeholders were purposively selected due to their involvement in the design, implementation, monitoring or evaluation of nutrition-specific or –sensitive policies and programs (Table 5). Key informants were asked to identify and refer the research team to other individuals with knowledge and expertise in the area of nutrition, policy, and stunting reduction. Sampling strategies helped to ensure a range of diverse perspectives at national and community levels.

Focus communities were selected to ensure coverage of perspectives from different geographic regions, by selecting one region from the North and one region from the South of the Kyrgyz Republic. In the North, Talas oblast was selected due to the substantial burden and high prevalence of stunting experienced during the 2000s. Since this time, several pilot nutrition programs have been implemented in Talas oblast and conducting semi-structured interviews with childcare workers and focus group discussions (FGDs) with mothers in communities aimed to identify key successes and challenges towards implementation of these initiatives. The Batken oblast was identified as the focus community in the Southern region, as these communities have faced problems with irrigation and limited access to clean, which may have influenced progress towards the reduction of stunting in this region. Both of these regions have had substantial reduction in stunting prevalence over the study period. Individuals were contacted and recruited by phone.

## Supplementary Table 1 Inclusion Criteria

| **Type of Stakeholder** | **Inclusion Criteria** |
| --- | --- |
| National Stakeholder | - Unpaid/paid decision-makers in national government structures. Examples include: Ministry of Health, or Ministry of Agriculture. - Unpaid/paid representative for an international donor (bilateral/multilateral), donor-funded program or local or international NGO/CSO. For example: World Food Programme, UNICEF, MercyCorps, or USAID project “Spring”. |
| Community Child Health Workers | - Unpaid/paid community representatives that interact with children, up to 5 years of age, including employees of rural health posts, members of local women’s councils, members of Village Health Committees, health care workers, pediatricians, kindergarten workers, informal community leaders and social workers. |
| Mothers in Communities | - Women that gave birth to a child between 1992 to 1997; - Women that gave birth to a child between 2012 to 2017; and - Currently lives in Talas or Batken oblast |

**Research Methods**

Primary qualitative data collection methods involved conducting in-depth interviews (IDI) with key informants at national and community levels, and FGDs with mothers in communities. Data collection was conducted from September to December 2017. Separate FGD and IDI guides for national and community stakeholders, and mothers were developed (Appendix 7-10).

*In-depth Interviews*

In-depth interviews were conducted with national stakeholders in Bishkek and included nutrition experts, representatives from multi- and bilateral international donor organizations, representatives of related ministries (Ministry of Health, Ministry of Health, Ministry of Social Protection, Ministry of Agriculture), and state agencies (National Center for Maternity and Childhood Protection). Respondents were asked to describe key nutrition-specific and –sensitive events (policies/strategies/laws/programs) in Kyrgyzstan including interventions, contexts, key stakeholders, as well as successful factors and barriers to implementation. In-depth interviews with national stakeholders also aimed to understand key trends in child undernutrition in Kyrgyzstan over time and to discuss contextual factors that represent enablers or drivers of change in child malnutrition indicators, including socioeconomic and lifestyle determinants.

In-depth interviews were also conducted at community level with young childcare workers in two oblasts (Talas and Batken) to outline community-level perspectives and experiences on transitions in nutrition and stunting in Kyrgyzstan. These interviews explored how contextual factors including changes in the social and economic situation, access to key resources (e.g., water/sanitation, health services), and changes in dietary intake were experienced at the community level. Further, key recommendations for pregnancy, breastfeeding and child nutrition were identified, and stakeholders evaluated changes in local water/sanitation/hygiene practices. In addition, key nutrition-specific and –sensitive events that community representatives felt are responsible for driving stunting reductions were identified and explored potential facilitators and challenges for implementation from a community, grassroots perspective.

*Focus Group Discussions*

FGDs were conducted in two regions (Talas and Batken) with mothers who gave birth to children during 1992-1997 and 2012-2017 periods. Women in both groups shared their experiences of and perspectives on: i) pregnancy and breastfeeding (e.g., breastfeeding promotion by health professions, length of breastfeeding, complementary feeding practices); ii) child nutrition (e.g., children’s diets, provision of vitamins/supplement); iii) hygiene (e.g., access to clean drinking water, handwashing, and experiences of infectious diseases); and iv) general trends and practices for their children’s nutrition (e.g., local improvements, nutrition practices/principles followed, policies/programs). Responses for each group were analyzed separately, and compared to identify trends over time for key contextual factors, and community perspectives on the stunting transition in their communities.

**Qualitative Data Analysis**

Data generated during semi-structured interviews and FGDs were analyzed using the UNICEF Nutrition Framework (76), Lancet Nutrition framework (51), and the adapted framework for the country case studies (Figures 5 & 6). These conceptual frameworks guided the qualitative analysis and interpretation of key determinants and contextual factors, as well as facilitators and barriers to nutrition-specific and –sensitive events. The qualitative analysis explored distal/basic causes (e.g., GDP, education, political context), nutrition-sensitive and -specific programs, underlying causes (e.g., inadequate feeding practices, and food insecurity, inadequate care and health services and unhealthy environment), and proximal/immediate causes (e.g., maternal characteristics, inadequate dietary intake, disease, and child characteristics). Responses from national and community level stakeholders were analyzed separately. Thematic analysis was conducted to explore key themes that emerged based on stunting determinants including socioeconomic status (e.g., living conditions), migration, hygiene and sanitation, and nutrition and eating behaviours.

All interviews were audio recorded with permission from research participants. Interviews in Bishkek with national stakeholders were conducted in Russian, while FGDs with mothers and community stakeholder interviews were conducted primarily in Kyrgyz. Interviews were translated into English, transcribed for analysis and anonymized through assigning interviews a unique numerical code. During the interviews, additional notes were taken by interviewers. All in-depth expert interviews were conducted by the Institute of Public Policy and Administration (IPPA) team, at the University of Central Asia in Kyrgyzstan. Each interview lasted approximately one hour, ranging from 20 minutes to over 2 hours.

Reflexivity also represented an important analytic tool in this mixed methods study, as the team aimed to produce credible and robust research through considering the research process (e.g., “how, where, when and by whom data were collected”) and ensuring methodological transparency (86). The team was composed of researchers from institutions in both the Kyrgyz Republic and Canada, with the generation and analysis of qualitative data led largely by the in-country research team (RM, ZE, MI and DK). The first author (JW), a Canadian PhD candidate with extensive qualitative research training and experience, oversaw and provided feedback and support for the qualitative and policy analysis components of the study. All authors contributed to the writing, provided feedback on the narrative and story of stunting decline in Kyrgyzstan and critically reviewed the manuscript.

The Consolidated Criteria for Reporting Qualitative Research (COREQ) checklist (87) represented an overall guide to reporting of qualitative study results. In describing our qualitative methods and dissemination of results we outlined: i) our research team and reflexivity (domain 1); ii) study design, including strategies for sampling/participant selection, research setting and data collection (domain 2); and iii) our approach to data analysis and presentation of findings, including the coding process, thematic analysis, selection/presentation of quotes, clarity of major/minor themes (domain 3).

*Ethics Approval*

The interview guide and purpose of the research were shared with research participants in advance, and participants were assured that responses would be anonymous. Participants provided oral consent to participate in the research study. This approach is in line with the University of Central Asia research ethics process.

# Supplementary Appendix 5 Quantitative Results

## Supplementary Figure 3A Spline analysis of inflection points of change in the slope of HAZ, 1997


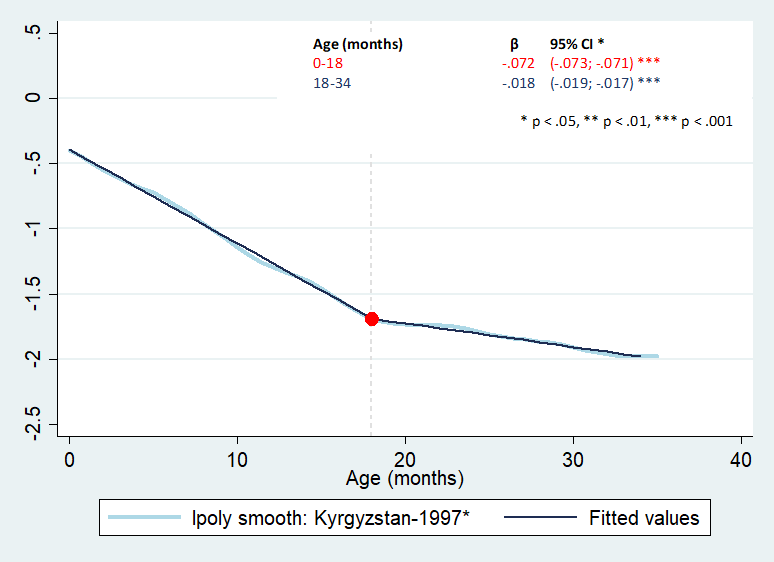


## Supplementary Figure 3B Spline analysis of inflection points of change in the slope of HAZ, 2014


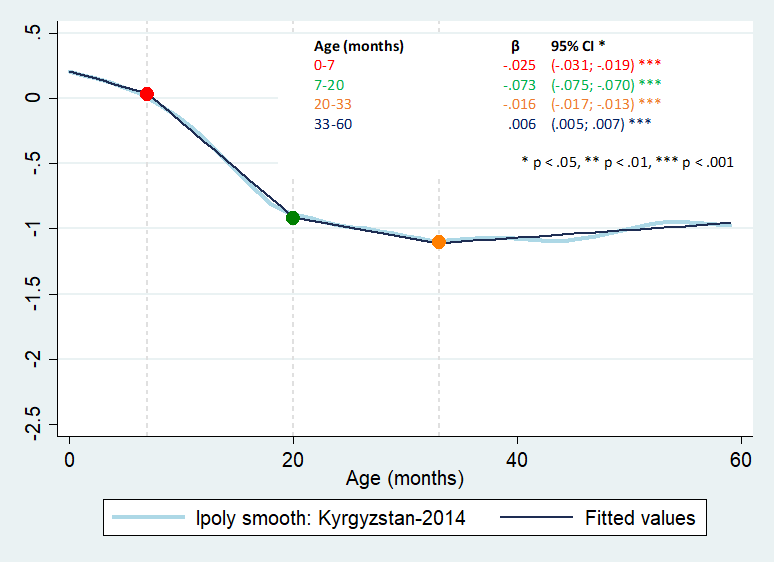


## Supplementary Figure 4A 1997 stunting prevalence by region


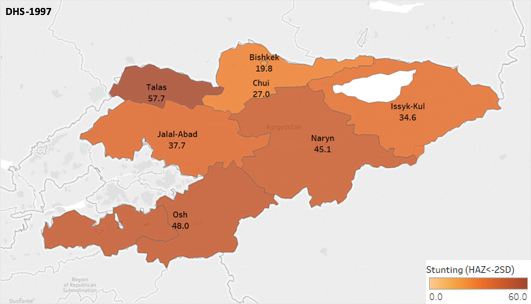


## Supplementary Figure 4B 2005 stunting prevalence by region


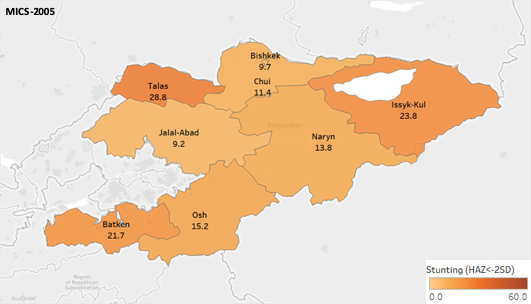


## Supplementary Figure 4C 2012 stunting prevalence by region


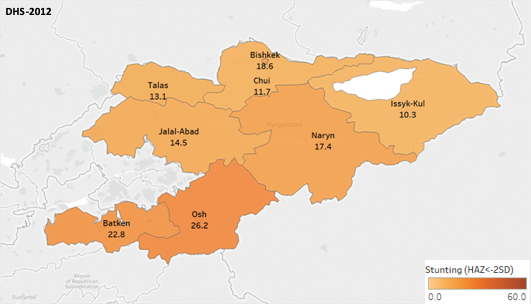


## Supplementary Figure 5A Change in absolute SII by year in Kyrgyz Republic

## Supplementary Figure 5B Change in absolute CIX by year in Kyrgyz Republic

## Supplementary Figure 6 Stunting prevalence by gender, 1997 – 2014


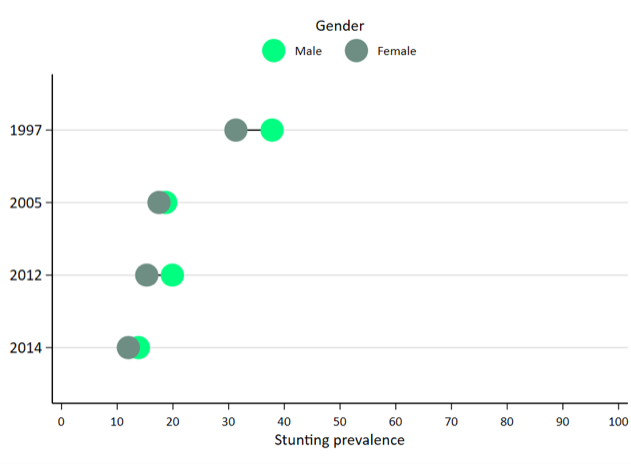


## Supplementary Table 2 Descriptive trends in stunting determinants in 1997 – 2012 in children <36 months

| **Domain/Indicator** |  | |  |  |
| --- | --- | --- | --- | --- |
|  | **DHS - 1997** | **DHS - 2012** | **Full time period (2012 - 1997)** | |
|  | **(n = 828)** | **(n =2096)** | **Change** | **p-value** |
| **Outcome** | | | | |
| Height for age z-score | -1.26 | -0.55 | 0.72 | <0.001 |
|  |  |  |  |  |
|  |  |  |  |  |
| Stunting | 31.44 | 16.25 | -15.19 | <0.001 |
| % of children below -2 SD |  |  |  |  |
|  |  |  |  |  |
| **Child Demographic** | | | | |
|  | | | | |
| Child sex | 51.55 | 51.78 | 0.24 | 0.929 |
| (% male, index child) |  |  |  |  |
|  |  |  |  |  |
| Child age | 16.20 | 15.43 | -0.76 | 0.129 |
| (months, index child) |  |  |  |  |
|  |  |  |  |  |
| **Distal level** | | | | |
| **Basic causes & Income poverty** | | |  |  |
| Wealth Index | 3.69 | 4.77 | 1.08 | <0.001 |
| (Score 0 – 10, household) |  |  |  |  |
|  |  |  |  |  |
| Wealth Index (six components using PCA) | 3.14 | 3.70 | 0.56 | 0.009 |
| (Score 0 – 10, household) |  |  |  |  |
|  |  |  |  |  |
| Wealth Index (nine components using PCA) | 4.05 | 4.68 | 0.63 | 0.006 |
| (Score 0 – 10, household) |  |  |  |  |
|  |  |  |  |  |
| Poverty | 70.16 | 30.05 | -40.11 | <0.001 |
| (% families below national lines, region) |  |  |  |  |
|  |  |  |  |  |
| Log GRP, per capita | 8.47 | 10.39 | 1.91 | <0.001 |
| (thousand soms/person, region) |  |  |  |  |
|  |  |  |  |  |
| Maternal education | 12.81 | 43.44 | 30.63 | <0.001 |
| (% higher education and above) |  |  |  |  |
|  |  |  |  |  |
| Paternal education level | 14.08 | 36.13 | 22.05 | <0.001 |
| (% higher education and above) |  |  |  |  |
|  |  |  |  |  |
| **Intermediate level** | | | | |
| **Inadequate feed practices and food** | | | | |
| Duration of breast feeding | 11.24 | 10.11 | -1.14 | 0.001 |
| (months, index child) |  |  |  |  |
|  |  |  |  |  |
| **Food insecurity** | | | | |
| Altitude | 1132.30 | 1146.09 | 13.79 | 0.758 |
| (m, household) |  |  |  |  |
|  |  |  |  |  |
| **Inadequate care and health services** | | | | |
| DPT vaccine [3 doses] | 89.50 | 94.53 | 5.03 | <0.001 |
| (%, index child) |  |  |  |  |
|  |  |  |  |  |
| Measles vaccine | 56.87 | 68.25 | 11.38 | <0.001 |
| (%, index child) |  |  |  |  |
|  |  |  |  |  |
| **Unhealthy household environment** | | | | |
| Urban locality | 23.84 | 29.66 | 5.83 | 0.062 |
| (%, households) |  |  |  |  |
|  |  |  |  |  |
| Access to improved sanitation facility | 99.80 | 94.36 | -5.44 | <0.001 |
| (%, households) |  |  |  |  |
|  |  |  |  |  |
| Access to improved drinking water source | 75.90 | 85.51 | 9.61 | 0.057 |
| (%, households) |  |  |  |  |
|  |  |  |  |  |
| Number of household members | 6.50 | 5.91 | -0.60 | <0.001 |
| (Total, household) |  |  |  |  |
|  |  |  |  |  |
| **Proximal level** | | | | |
| **Disease** | | | | |
| ARI infection in last 2 weeks | 16.01 | 5.60 | -10.41 | <0.001 |
| (%, index child) |  |  |  |  |
|  |  |  |  |  |
| Diarrhea infection in last 2 weeks | 18.39 | 6.50 | -11.89 | <0.001 |
| (%, index child) |  |  |  |  |
|  |  |  |  |  |
| **Inadequate dietary intake** | | | | |
| Intake of calories | 2042.21 | 2172.87 | 130.66 | <0.001 |
| kcal/person/day |  |  |  |  |
|  |  |  |  |  |
| Intake of fats | 44.46 | 62.60 | 18.14 | <0.001 |
| g/person/day |  |  |  |  |
|  |  |  |  |  |
| Intake of proteins | 51.09 | 61.28 | 10.19 | <0.001 |
| g/person/day |  |  |  |  |
|  |  |  |  |  |
| **Child characteristics** | | | | |
| Low birthweight | 5.06 | 5.64 | 0.58 | 0.641 |
| (% index child) |  |  |  |  |
|  |  |  |  |  |
| **Maternal characteristics** | | | | |
| Maternal age | 27.35 | 28.12 | 0.77 | 0.025 |
| (years, mothers) |  |  |  |  |
|  |  |  |  |  |
| Adolescent birth (<18 years of age) | 1.24 | 0.72 | -0.52 | 0.225 |
| (%, mothers for index child birth) |  |  |  |  |
|  |  |  |  |  |
| Older mother birth (≥35 years) | 8.76 | 12.65 | 3.89 | 0.007 |
| (%, mothers for index child birth) |  |  |  |  |
|  |  |  |  |  |
| Anemia during pregnancy | 42.53 | 38.57 | -3.96 | 0.113 |
| (%, mothers) |  |  |  |  |
|  |  |  |  |  |
| Body mass index  (kg/m^2,^ mothers) | 22.61 | 23.69 | 1.08 | <0.001 |
|  |  |  |  |  |
|  |  |  |  |  |
| Height | 157.78 | 159.19 | 1.41 | <0.001 |
| (cm, mothers) |  |  |  |  |
|  |  |  |  |  |
| Parity | 2.82 | 2.48 | -0.35 | 0.001 |
| (Total children, mother) |  |  |  |  |
|  |  |  |  |  |
| Interpregnancy interval (months) | 41.40 | 48.11 | 6.72 | <0.001 |
| (index mother) |  |  |  |  |
|  |  |  |  |  |

## Supplementary Table 3 Decomposition analysis for children among <36 months from 1997-2012

| **Factors** | **Estimated coefficient** | **Mean difference (2012 - 1997)** | **Predicted change in HAZ** | **Share of predicted change in (%)** |
| --- | --- | --- | --- | --- |
| HAZ | - | 0.716 | 0.637 | 88.9% |
| Wealth accumulation | 0.018 | 0.632 | 0.012 | 1.8% |
| Poverty reduction | -0.010 | -40.114 | 0.389 | 61.0% |
| Paternal education (higher & above) | 0.188 | 0.221 | 0.041 | 6.5% |
| Low birthweight (<2500gms) | -0.684 | 0.006 | -0.004 | -0.6% |
| Mother age (in years) | 0.025 | 0.771 | 0.019 | 3.0% |
| Maternal BMI (kg/m^2^) | 0.017 | 1.075 | 0.019 | 2.9% |
| Maternal height (cm) | 0.048 | 1.414 | 0.068 | 10.7% |
| Parity | -0.104 | -0.347 | 0.036 | 5.7% |
| Others | - | - | 0.057 | 9.0% |

## Supplementary Figure 7 Decomposing predicted changes in HAZ among children <36 months (i.e. relative ranking of product coefficients for determinant domains) from 1997-2012

## Supplementary Table 4 Decomposition analysis for children among 6-23 months from 1997-2012

| **Factors** | **Estimated coefficient** | **Mean difference (2012 - 1997)** | **Predicted change in HAZ** | **Share of predicted change in (%)** |
| --- | --- | --- | --- | --- |
| HAZ | - | 0.951 | 0.706 | 74.3% |
| Wealth accumulation | 0.034 | 0.897 | 0.030 | 4.3% |
| Poverty reduction | -0.010 | -40.966 | 0.390 | 55.2% |
| Paternal education (higher & above) | 0.202 | 0.198 | 0.040 | 5.7% |
| Daily intake of calories | 0.001 | 139.329 | 0.111 | 15.7% |
| Low birthweight (<2500gms) | -0.735 | 0.021 | -0.016 | -2.2% |
| Maternal height (cm) | 0.058 | 1.440 | 0.084 | 11.9% |
| Interpregnancy interval (months) | 0.006 | 4.134 | 0.025 | 3.6% |
| Others | - | - | 0.041 | 5.8% |

## Supplementary Figure 8 Decomposing predicted changes in HAZ among children 6-23 months (i.e. relative ranking of product coefficients for determinant domains) from 1997-2012

## Supplementary Table 5 Difference-in-differences multivariable regression for children under 36 months years from 1997 – 2012

| Domain/ Indicator | Outcome= HAZ | |
| --- | --- | --- |
|  | (z-score for children under 36 months) | |
|  | Period 1997-2012 | |
|  | Bivariate regression coefficient | Final multivariable regression coefficient* |
|  | b estimate (95% CL) | b estimate (95% CL) |
|  | p-value | p-value |
|  |  |  |
| **Distal level^a^** | | |
| **Basic causes & Income poverty** | | |
| Wealth Index (nine component using PCA) | 3.03 (-2.324; 8.384) | 0.018 (-0.001; 0.038) |
| (score 0 – 10, household) | 0.266 | 0.067 |
|  |  |  |
| Wealth Index # Year | -0.001 (-0.004; 0.001) |  |
|  | 0.271 | -- |
|  |  |  |
| Poverty line | -0.01 (-0.015; -0.004) | -0.0097 (-0.0189; -0.0005) |
| (% families below, region) | 0.001 | 0.039 |
|  |  |  |
| Poverty line # Year | 0.00074 (-0.00005; 0.00153) |  |
|  | 0.065 | -- |
|  |  |  |
| Log GRP, per capita | 22.429 (-16.796; 61.655) |  |
| (thousand soms/person, region) | 0.262 | -- |
|  |  |  |
| Log GRP # Year | -0.011 (-0.031; 0.008) |  |
| (thousand soms/person, region) | 0.265 | -- |
|  |  |  |
| Maternal education | 9.282 (-44.615; 63.179) |  |
| (% higher education and above) | 0.735 | -- |
|  |  |  |
| Maternal education # year | -0.005 (-0.031; 0.022) |  |
|  | 0.741 | -- |
|  |  |  |
| Paternal education | 27.049 (-27.025; 81.123) | 0.188 (0.037; 0.339) |
| (% higher education and above) | 0.326 | 0.015 |
|  |  |  |
| Paternal education # Year | -0.013 (-0.04; 0.014) |  |
|  | 0.33 | -- |
|  |  |  |
| **% of variance explained by covariates^2^** | | 16.30% |
| **Intermediate level^b^** | | |
| **Inadequate feed practices and food** | | |
| Duration of breast feeding | -1.57 (-4.268; 1.128) |  |
| (months, index child) | 0.253 | -- |
|  |  |  |
| Duration of breast feeding # Year | 0.001 (-0.001; 0.002) |  |
|  | 0.274 | -- |
|  |  |  |
| **Food insecurity** | | |
| Altitude | -0.03838 (-0.07418; -0.00259) | 0.00019 (0.00001; 0.00037) |
| (m, household) | 0.036 | 0.036 |
|  |  |  |
| Altitude # Year | 0.000019 (0.000001; 0.000037) |  |
|  | 0.036 | -- |
|  |  |  |
| **Inadequate health services** | | |
| DPT vaccine [3 doses] | -104.52 (-167.428; -41.612) | -85.725 (-146.726; -24.724) |
| (%, index child) | 0.001 | 0.006 |
|  |  |  |
| DPT # Year | 0.052 (0.02; 0.083) | 0.043 (0.012; 0.073) |
|  | 0.001 | 0.006 |
|  |  |  |
| Measles vaccine | -32.957 (-68.729; 2.815) |  |
| (%, index child) | 0.071 | -- |
|  |  |  |
| Measles vaccine # Year | 0.016 (-0.002; 0.034) |  |
|  | 0.077 | -- |
|  |  |  |
| **Unhealthy household environment** | | |
| Urban locality | 30.215 (-21.234; 81.664) |  |
| (%, households) | 0.249 | -- |
|  |  |  |
| Urban locality # Year | -0.015 (-0.041; 0.011) |  |
|  | 0.252 | -- |
|  |  |  |
| Access to improved sanitation facilities | -89.499 (-171.168; -7.831) |  |
| (%, households) | 0.032 | -- |
|  |  |  |
| Access to improved sanitation facilities # Year | 0.045 (0.004; 0.085) |  |
|  | 0.032 | -- |
|  |  |  |
| Access to improved drinking water source | 46.92 (-2.915; 96.754) |  |
| (%, households) | 0.065 | -- |
|  |  |  |
| Access to improved drinking water source # Year | -0.023 (-0.048; 0.002) |  |
|  | 0.066 |  |
|  |  |  |
| Number of household members | -2.447 (-11.882; 6.989) |  |
| (total, household) | 0.61 | -- |
|  |  |  |
| Number of household members # Year | 0.001 (-0.003; 0.006) |  |
|  | 0.611 | -- |
|  |  |  |
| **% of variance explained by covariates^2^** | | 17.00% |
| **Proximal level^c^** | | |
| **Infections** | | |
| ARI infection in last 2 weeks | -22.919 (-85.125; 39.286) |  |
| (%, index child) | 0.469 | -- |
|  |  |  |
| ARI infection in last 2 weeks # Year | 0.011 (-0.02; 0.042) |  |
|  | 0.472 | -- |
|  |  |  |
| Diarrhea infection in last 2 weeks | -24.123 (-88.707; 40.461) |  |
| (%, index child) | 0.463 | -- |
|  |  |  |
| Diarrhea infection in last 2 weeks # Year | 0.012 (-0.02; 0.044) |  |
|  | 0.462 | -- |
|  |  |  |
| **Inadequate Dietary Intake** | | |
| Intake of calories | -0.074 (-0.181; 0.032) |  |
| (kcal/person/day, region) | 0.17 | -- |
|  |  |  |
| Intake of calories # Year | 0.00004 (-0.00002; 0.00009) |  |
|  | 0.168 | -- |
|  |  |  |
| Intake of fats | 2.644 (0.062; 5.226) |  |
| (g/person/day, region) | 0.045 | -- |
|  |  |  |
| Intake of fats# Year | -0.00131 (-0.0026; -0.00003) |  |
|  | 0.046 | -- |
|  |  |  |
| Intake of proteins | -2.739 (-6.712; 1.235) |  |
| (g/person/day, region) | 0.176 |  |
|  |  |  |
| Intake of proteins # Year | 0.001 (-0.001; 0.003) |  |
|  | 0.175 | -- |
|  |  |  |
| **Children Characteristics** | | |
| Low birthweight | -0.792 (-1.013; -0.571) | -0.687 (-0.917; -0.457) |
| (%, index child) | <0.001 | <0.001 |
|  |  |  |
| Low birthweight # Year | -0.003 (-0.033; 0.028) |  |
|  | 0.861 | -- |
|  |  |  |
| **Maternal Characteristics** | | |
| Maternal age | 0.777 (-2.148; 3.702) | 0.026 (0.008; 0.043) |
| (years, mothers) | 0.602 | 0.004 |
|  |  |  |
| Maternal age # Year | -0.0004 (-0.0019; 0.0011) |  |
|  | 0.596 | -- |
|  |  |  |
| Adolescent birth (<18 years of age) | -85.857 (-277.082; 105.368) |  |
| (%, mothers for index birth) | 0.378 | -- |
|  |  |  |
| Adolescent birth # Year | 0.043 (-0.052; 0.139) |  |
|  | 0.376 | -- |
|  |  |  |
| Older mother birth (≥35 years)  (%, mothers for index child birth) | 4.612 (-60.96; 70.185) |  |
|  | 0.89 | -- |
|  |  |  |
| Older mother birth # Year | -0.002 (-0.035; 0.03) |  |
|  | 0.891 | -- |
|  |  |  |
| Anemia during pregnancy | 17.007 (-23.572; 57.586) |  |
| (%, mothers) | 0.41 | -- |
|  |  |  |
| Anemia during pregnancy # Year | -0.008 (-0.029; 0.012) |  |
|  | 0.411 | -- |
|  |  |  |
| Body mass index | 4.191 (-0.322; 8.704) | 5.277 (1.283; 9.27) |
| (kg/m^2^, mothers) | 0.069 | 0.01 |
|  |  |  |
| Body mass index # Year | -0.0021 (-0.0043; 0.0002) | -0.003 (-0.005; -0.001) |
|  | 0.07 | 0.01 |
|  |  |  |
| Height | 1.807 (-1.46; 5.074) | 0.05 (0.039; 0.061) |
| (cm, mothers) | 0.277 | <0.001 |
|  |  |  |
| Height # Year | -0.001 (-0.003; 0.001) |  |
|  | 0.292 | -- |
|  |  |  |
| Parity | -5.795 (-16.378; 4.789) | -0.109 (-0.175; -0.043) |
| (Total children, mother) | 0.282 | 0.001 |
|  |  |  |
| Parity # Year | 0.003 (-0.002; 0.008) |  |
|  | 0.289 | -- |
|  |  |  |
| Interpregnancy interval (months) | 0.475 (-0.258; 1.208) |  |
| (index mother) | 0.203 | -- |
|  |  |  |
| Inte pregnancy interval # Year | -0.0002 (-0.0006; 0.0001) |  |
|  | 0.209 | -- |
|  |  |  |
| **% of variance explained by covariates** | | 21.70% |
| **Time** |  |  |
| Year | 0.048 (0.037; 0.058) | 0.035 (-0.023; 0.094) |
|  | <0.001 | 0.234 |
|  |  |  |

* Adjusted for child age, sex, and region

## Supplementary Table 6 Difference-in-differences multivariable regression for children aged 6-23 months from 1997 – 2012

| Domain/ Indicator | Outcome= HAZ | |
| --- | --- | --- |
|  | (z-score for children 6 - 23 months) | |
|  | Period 1997-2012 | |
|  | Bivariate regression coefficient | Final multivariable regression coefficient* |
|  | b estimate (95% CL) | b estimate (95% CL) |
|  | p-value | p-value |
|  |  |  |
| **Distal level^a^** | | |
| **Basic causes & Income poverty** | | |
| Wealth Index (nine component) | 3.149 (-4.278; 10.575) | 0.036 (0.011; 0.061) |
| (score 0 – 10, household) | 0.405 | 0.005 |
|  |  |  |
| Wealth Index # Year | -0.002 (-0.005; 0.002) |  |
|  | 0.411 | -- |
|  |  |  |
| Poverty line | -1.184 (-3.198; 0.829) |  |
| (% families below, region) | 0.248 | -- |
|  |  |  |
| Poverty line # Year | 0.00058 (-0.00042; 0.00159) |  |
|  | 0.255 | -- |
|  |  |  |
| Log GRP, per capita | 8.211 (-43.017; 59.44) |  |
| (thousand soms/person, region) | 0.753 | -- |
|  |  |  |
| Log GRP # Year | -0.004 (-0.029; 0.022) |  |
|  | 0.762 | -- |
|  |  |  |
| Maternal education | 13.787 (-67.976; 95.55) |  |
| (% higher education and above) | 0.74 | -- |
|  |  |  |
| Maternal education # year | -0.007 (-0.047; 0.034) |  |
|  | 0.746 | -- |
|  |  |  |
| Paternal education | 35.033 (-41.192; 111.259) | 0.201 (0.004; 0.398) |
| (% higher education and above) | 0.367 | 0.046 |
|  |  |  |
| Paternal education # Year | -0.017 (-0.055; 0.021) |  |
|  | 0.371 | -- |
|  |  |  |
| **% of variance explained by covariates** | | 17.10% |
| **Intermediate level^b^** | | |
| **Inadequate feed practices and food** | | |
| Duration of breast feeding | 0.625 (-4.549; 5.798) |  |
| (months, index child) | 0.812 | -- |
|  |  |  |
| Duration of breastfeeding # Year | 0 (-0.003; 0.002) |  |
|  | 0.789 | -- |
|  |  |  |
| **Food insecurity** | | |
| Altitude | -0.02314 (-0.0748; 0.02851) |  |
| (m, houshold) | 0.379 | -- |
|  |  |  |
| Altitude # Year | 0.000012 (-0.000014; 0.000037) |  |
|  | 0.38 | -- |
|  |  |  |
| **Inadequate health services** | | |
| DPT vaccine [3 doses] | 2.746 (-302.343; 307.835) |  |
| (%, index child) | 0.986 | -- |
|  |  |  |
| DPT vaccine # Year | -0.001 (-0.153; 0.151) |  |
|  | 0.988 | -- |
|  |  |  |
| Measles vaccine | -1.875 (-50.286; 46.537) |  |
| (%, index child) | 0.939 | -- |
|  |  |  |
| Measles vaccine # Year | 0.001 (-0.023; 0.025) |  |
|  | 0.95 | -- |
|  |  |  |
| Skilled attendant at birth | -162.921 (-479.684; 153.842) |  |
| (%, mothers) | 0.312 | -- |
|  |  |  |
| Skilled attendant at birth # year | 0.082 (-0.076; 0.24) |  |
|  | 0.309 | -- |
|  |  |  |
| 4+ antenatal care visits | 4.469 (-64.116; 73.054) |  |
| (%, mothers) | 0.898 | -- |
|  |  |  |
| 4+ antenatal care visits # year | -0.002 (-0.036; 0.032) |  |
|  | 0.902 | -- |
|  |  |  |
| **Unhealthy household environment** | | |
| Urban locality | 39.2 (-41.493; 119.892) |  |
| (%, households) | 0.34 | -- |
|  |  |  |
| Urban locality # Year | -0.019 (-0.06; 0.021) |  |
|  | 0.343 | -- |
|  |  |  |
| Access to improved sanitation facilities |  |  |
| (%, households) | -- | -- |
|  |  |  |
| Access to improved sanitation facilities # Year |  |  |
|  | -- | -- |
|  |  |  |
| Access to improved drinking water source | 31.077 (-36.441; 98.595) |  |
| (%, households) | 0.366 | -- |
|  |  |  |
| Access to improved drinking water source # Year | -0.015 (-0.049; 0.018) |  |
|  | 0.37 | -- |
|  |  |  |
| Number of household members | -8.501 (-20.611; 3.61) |  |
| (total, household) | 0.168 | -- |
|  |  |  |
| Number of household members # Year | 0.004 (-0.002; 0.01) |  |
|  | 0.17 | -- |
|  |  |  |
| **% of variance explained by covariates** | | 17.10% |
| **Proximal level^c^** | | |
| **Infections** | | |
| ARI infection in last 2 weeks | -74.79 (-148.031; -1.55) | -54.2768 (-108.7368; 0.1832) |
| (%, index child) | 0.045 | 0.051 |
|  |  |  |
| ARI infection in last 2 weeks # Year | 0.037 (0.001; 0.074) | 0.027 (-0.0002; 0.0542) |
|  | 0.046 | 0.052 |
|  |  |  |
| Diarrhea infection in last 2 weeks | -22.77 (-97.295; 51.755) |  |
| (%, index child) | 0.548 | -- |
|  |  |  |
| Diarrhea infection in last 2 weeks # Year | 0.011 (-0.026; 0.049) |  |
|  | 0.548 | -- |
|  |  |  |
| **Inadequate Dietary Intake** | | |
| Intake of calories | -0.071 (-0.23; 0.088) | 0.001 (0.0001; 0.0019) |
| (kcal/person/day, region) | 0.381 | 0.034 |
|  |  |  |
| Intake of calories # Year | 0.00004 (-0.00004; 0.00011) |  |
|  | 0.377 | -- |
|  |  |  |
| Intake of fats | 3.832 (0.379; 7.286) | -4.139 (-7.573; -0.706) |
| (g/person/day, region) | 0.03 | 0.018 |
|  |  |  |
| Intake of fats # Year | -0.00191 (-0.00363; -0.00019) | 0.002 (0; 0.004) |
|  | 0.03 | 0.018 |
|  |  |  |
| Intake of proteins | -2.313 (-7.86; 3.235) |  |
| (g/person/day, region) | 0.413 | -- |
|  |  |  |
| Intake of proteins # Year | 0.001 (-0.002; 0.004) |  |
|  | 0.412 | -- |
|  |  |  |
| Animal source food in last 24 hours | 25.863 (-34.32; 86.046) |  |
| (%, index child) | 0.399 | -- |
|  |  |  |
| Animal source food in last 24 hours # year | -0.013 (-0.043; 0.017) |  |
|  | 0.396 | -- |
|  |  |  |
| Vegetable in the last 24 hours | -8.829 (-60.459; 42.802) |  |
| (%, index child) | 0.737 | -- |
|  |  |  |
| Vegetable in the last 24 hours # year | 0.004 (-0.021; 0.03) |  |
|  | 0.742 | -- |
|  |  |  |
| **Children Characteristics** | | |
| Low birthweight | 29.643 (-56.403; 115.689) | -0.73 (-1.088; -0.371) |
| (%, index child) | 0.498 | <0.001 |
|  |  |  |
| Low birthweight # Year | -0.015 (-0.058; 0.028) |  |
|  | 0.487 | -- |
|  |  |  |
| **Maternal Characteristics** | | |
| Maternal age | 2.638 (-1.76; 7.036) |  |
| (years, mothers) | 0.239 | -- |
|  |  |  |
| Age# Year | -0.0013 (-0.0035; 0.0009) |  |
|  | 0.238 | -- |
|  |  |  |
| Adolescent birth (<18 years of age) | -67.864 (-342.445; 206.717) |  |
| (% mothers for index child within last 5 years) | 0.627 | -- |
|  |  |  |
| Adolescent birth # Year | 0.034 (-0.103; 0.171) |  |
|  | 0.624 | -- |
|  |  |  |
| Older mother birth (≥35 years)  (% mothers for index child within last 5 years) | 9.968 (-68.889; 88.826) |  |
|  | 0.804 | -- |
|  |  |  |
| Older mother birth # Year | -0.005 (-0.044; 0.034) |  |
|  | 0.807 | -- |
|  |  |  |
| Anemia during pregnancy | 9.173 (-45.824; 64.171) |  |
| (%, mothers) | 0.743 | -- |
|  |  |  |
| Anemia during pregnancy # Year | -0.005 (-0.032; 0.023) |  |
|  | 0.74 | -- |
|  |  |  |
| Body mass index | 5.631 (-0.324; 11.586) | 6.815 (1.76; 11.87) |
| (kg/m^2^, mothers) | 0.064 | 0.008 |
|  |  |  |
| Body mass index # Year | -0.0028 (-0.0058; 0.0002) | -0.003 (-0.006; -0.001) |
|  | 0.064 | 0.009 |
|  |  |  |
| Height | 0.048 (-4.275; 4.371) | 0.06 (0.045; 0.074) |
| (cm, mothers) | 0.983 | <0.001 |
|  |  |  |
| Height # Year | 0.00001 (-0.00215; 0.00216) |  |
|  | 0.994 | -- |
|  |  |  |
| Parity | 2.09 (-12.752; 16.931) |  |
| (Total children, mother) | 0.782 | -- |
|  |  |  |
| Parity # Year | -0.001 (-0.008; 0.006) |  |
|  | 0.772 | -- |
|  |  |  |
| Inter pregnancy interval (months) | 0.27 (-0.708; 1.249) | 0.006 (0.003; 0.009) |
| (index mother) | 0.587 | <0.001 |
|  |  |  |
| Inter pregnancy interval # Year | -0.0001 (-0.0006; 0.0004) |  |
|  | 0.598 | -- |
|  |  |  |
| **% of variance explained by covariates** | | 25.01% |
| **Time** |  |  |
| Year | 0.063 (0.049; 0.078) | -0.03 (-0.143; 0.083) |
|  | <0.001 | 0.6 |
|  |  |  |

* Adjusted for child age, sex, and region

## Supplementary Table 7 Difference-in-differences multivariable regression for children aged 2-5 years old from MICS surveys 2005/6 – 2014

| Domain/ Indicator | Outcome= HAZ | |
| --- | --- | --- |
|  | (z-score for children 24 - 59 months) | |
|  | Period 2005/06 - 2014 | |
|  | Bivariate regression coefficient | Final multivariable regression coefficient* |
|  | b estimate (95% CL) | b estimate (95% CL) |
|  | p-value | p-value |
|  |  |  |
| **Distal level^a^** | | |
| **Basic causes & Income poverty** | | |
| Wealth Index (nine component) | 10.461 (2.061; 18.861) | 0.037 (0.018; 0.056) |
| (score 0 – 10, household) | 0.015 | <0.001 |
|  |  |  |
| Wealth Index # Year | -0.005 (-0.009; -0.001) |  |
|  | 0.015 | -- |
|  |  |  |
| Poverty line | -0.415 (-2.604; 1.774) |  |
| (% families below, region) | 0.71 | -- |
|  |  |  |
| Poverty line # Year | 0.0002 (-0.00089; 0.00129) |  |
|  | 0.72 | -- |
|  |  |  |
| Log GRP, per capita | 74.542 (35.882; 113.202) | -0.9956 (-1.6822; -0.309) |
| (thousand soms/person, region) | <0.001 | 0.005 |
|  |  |  |
| Log GRP #Year | -0.037 (-0.056; -0.018) |  |
|  | <0.001 | -- |
|  |  |  |
| Maternal education | 75.336 (5.396; 145.276) | 0.2007 (0.0358; 0.3656) |
| (% higher education and above) | 0.035 | 0.017 |
|  |  |  |
| Maternal education # year | -0.037 (-0.072; -0.002) |  |
|  | 0.036 | -- |
|  |  |  |
| Paternal education | 37.101 (-40.28; 114.482) |  |
| (% higher education and above) | 0.347 | -- |
|  |  |  |
| Paternal education # Year | -0.018 (-0.057; 0.02) |  |
|  | 0.35 | -- |
|  |  |  |
| **% of variance explained by covariates^2^** | | 9.20% |
| **Intermediate level^b^** | | |
| **Inadequate health services** | | |
| DPT vaccine [3 doses] | 20.858 (-340.648; 382.363) | 0.716 (0.199; 1.234) |
| (%, index child) | 0.91 | 0.007 |
|  |  |  |
| DPT vaccine # Year | -0.01 (-0.19; 0.17) |  |
|  | 0.913 | -- |
|  |  |  |
| Measles vaccine | 173.73 (-267.201; 614.661) |  |
| (%, index child) | 0.44 | -- |
|  |  |  |
| Measles vaccine # Year | -0.086 (-0.306; 0.134) |  |
|  | 0.442 | -- |
|  |  |  |
| **Unhealthy household environment** | | |
| Urban locality | 107.05 (47.383; 166.717) |  |
| (%, households) | <0.001 | -- |
|  |  |  |
| Urban locality # Year | -0.053 (-0.083; -0.023) |  |
|  | <0.001 | -- |
|  |  |  |
| Access to improved sanitation facilities | 42.337 (-63.699; 148.373) | 0.264 (0.015; 0.512) |
| (%, households) | 0.434 | 0.038 |
|  |  |  |
| Access to improved sanitation facilities # Year | -0.021 (-0.074; 0.032) |  |
|  | 0.436 | -- |
|  |  |  |
| Access to improved drinking water source | 93.21 (10.334; 176.085) | 0.259 (0.01; 0.507) |
| (%, households) | 0.028 | 0.041 |
|  |  |  |
| Access to improved drinking water source # Year | -0.046 (-0.087; -0.005) |  |
|  | 0.028 | -- |
|  |  |  |
| Number of household members | -6.003 (-24.585; 12.578) |  |
| (total, household) | 0.526 | -- |
|  |  |  |
| Number of household members # Year | 0.003 (-0.0063; 0.0122) |  |
|  | 0.529 | -- |
|  |  |  |
| **% of variance explained by covariates^2^** | | 11.30% |
| **Proximal level^c^** | | |
| **Infections** | | |
| ARI infection in last 2 weeks | -67.312 (-218.43; 83.806) |  |
| (%, index child) | 0.382 | -- |
|  |  |  |
| ARI infection in last 2 weeks # Year | 0.034 (-0.042; 0.109) |  |
|  | 0.382 | -- |
|  |  |  |
| Diarrhea infection in last 2 weeks | -84.951 (-381.058; 211.155) |  |
| (%, index child) | 0.574 | -- |
|  |  |  |
| Diarrhea infection in last 2 years # Year | 0.042 (-0.105; 0.189) |  |
|  | 0.575 | -- |
|  |  |  |
| **Inadequate Dietary Intake** | | |
| Intake of calories | 0.223 (0.12; 0.326) | -25.87 (-43.183; -8.558) |
| (kcal/person/day, region) | <0.001 | 0.003 |
|  |  |  |
| Intake of calories # Year | -0.00011 (-0.00016; -0.00006) | 0.013 (0.004; 0.021) |
|  | <0.001 | 0.003 |
|  |  |  |
| Intake of fats | 6.038 (2.748; 9.328) |  |
| (g/person/day, region) | <0.001 | -- |
|  |  |  |
| Intake of fats # Year | -0.003 (-0.00463; -0.00136) |  |
|  | <0.001 | -- |
|  |  |  |
| Intake of proteins | 6.571 (3.307; 9.835) | 18.28 (-0.044; 36.603) |
| (g/person/day, region) | <0.001 | 0.051 |
|  |  |  |
| Intake of proteins # Year | -0.003 (-0.005; -0.002) | -0.00907 (-0.01817; 0.00003) |
|  | <0.001 | 0.051 |
|  |  |  |
| **Children Characteristics** | | |
| Low birthweight | 185.75 (-65.831; 437.331) |  |
| (%, index child) | 0.146 | -- |
|  |  |  |
| Low birthweight # Year | -0.093 (-0.218; 0.032) |  |
|  | 0.145 | -- |
|  |  |  |
| **Maternal Characteristics** | | |
| Maternal age | 1.19 (-3.535; 5.915) |  |
| (years, mothers) | 0.622 | -- |
|  |  |  |
| Maternal age # Year | -0.0006 (-0.0029; 0.0018) |  |
|  | 0.624 |  |
|  |  |  |
| Adolescent birth (<18 years of age) | 60.911 (-126.117; 247.939) |  |
| (% mothers for index child within last 5 years) | 0.523 | -- |
|  |  |  |
| Adolescent birth # Year | -0.03 (-0.124; 0.063) |  |
|  | 0.522 | -- |
|  |  |  |
| Older mother birth (≥35 years) | -22.623 (-95.375; 50.13) |  |
| (% mothers for index child within last 5 years) | 0.542 | -- |
|  |  |  |
| Older mother birth # Year | 0.011 (-0.025; 0.047) |  |
|  | 0.54 | -- |
|  |  |  |
| Parity | -15.065 (-33.712; 3.582) |  |
| (Total children, mother) | 0.113 | -- |
|  |  |  |
| Parity # Year | 0.007 (-0.002; 0.017) |  |
|  | 0.115 | -- |
|  |  |  |
| **% of variance explained by covariates** | | 12.60% |
| **Time** |  |  |
| Year | -0.015 (-0.03; 0) | 0.005 (-0.376; 0.386) |
|  | 0.053 | 0.98 |
|  |  |  |

* Adjusted for child age, sex, and region

# Supplementary Appendix 6 Programs and Policies

The analysis of programs and policies provided a description of key initiatives using the below tool.

**Definitions for Programs and Policy Timeline**

- 1. **Program/policy name**: In English and local language, any acronyms used for the program/policy in English and local language, what these acronyms mean
  2. **Timeline:** Total duration of the project, starting with initiation discussions through to roll out and scale up and endpoint (if it has ended/is scheduled to end)
  3. **Reforms:** If the program has changed, evolved, expanded, changed names or faces. If the program/policy reform was significant, outline the reformed version in its own section of the timeline (based on your discretion).
  4. **List of sources used** (numbers that correspond to the bibliography). Also add relevant source number used following each of the main sections below. Keep records of information obtained by personal communication (e.g. from a key stakeholder interview) so that these may be properly cited.

**Main Overview:**

- 1. **Description:** In 5-6 sentences and without using acronyms, this section should include:
     - An overview of the major objectives of the program/policy
     - Area of the country where the program/policy was delivered
     - Population reached (number of people reached, setting)
     - Details of scale-up
  2. **Delivery Platform:** *is the channel by which a nutrition-specific intervention reaches the population in need***.^[[1]](#footnote-1)^**
     - This section of the program/policy overview should be 2-3 sentences in length, including: a) a short rationale for what type of delivery platform the program falls under based on its description and component parts (1-2 sentences); b) a short explanation of what the delivery platform is (1 sentence, see below for details on the five types of delivery platform).
     - **Types of Delivery Platforms:**
       - **Fortification-based platforms** focus on improving the nutritional value of staple foods, which can be done at four levels: mass/universal fortification, targeted fortification, household fortification and bio-fortification. Mass/universal fortification is done in conjunction with food production industries (e.g. flour fortification); targeted fortification is done for particularly nutritionally vulnerable populations or in emergency situations (e.g. micronutrient powders); household fortification involves the fortification of particular foods consumed especially by women and children (e.g. fortified lipid spreads); and bio-fortification involves increasing key nutrients within food crops. ^1,^^[[2]](#footnote-2)^
       - **Financial incentive-based platforms** include the delivery of cash transfers (either conditional or unconditional), vouchers or food stamp interventions, as well as the removal of user fees in order to eliminate financial barriers and enable individuals to better their health. ^1,^^[[3]](#footnote-3)^
       - **Community-based platforms** include the delivery of interventions by health care workers through outreach channels at the community-level, as well as the implementation of interventions that are driven by community groups themselves. ^1,3^
       - **School-based platforms** focus on the delivery of targeted school feeding programs, and the promotion of health and nutrition information to children while they are attending school.^1^
       - **Technology-based platforms** include the delivery of nutrition information and communication-based interventions using mass or social media as well as mobile health (also known as mHealth), via mobile phones (e.g. SMS messaging).^3^
  3. **Key Stakeholders:** Identify important groups for this program/policy. Break it down into conception of program or policy, advocacy/promotion, funding, rolling out/scale up, implementation and monitoring and evaluation. Make explicit who was responsible for what in the program/policy, if one category doesn’t apply we can say “N/A.”
  4. **Initiation Process**: In 5-6 sentences describe how the program/policy was born. This includes information on what happened before the program was initiated.
  5. **Key Components**: This should be the most detailed section in the timeline. Includes the following information:
- Main parts of the program/policy
- Goals of the program/policy—what is it trying to achieve?
- Targeted % change in each indicator outlined within the program/policy
- How many people does it aim to reach
- Any sub-components/ initiatives of the larger program/policy
- Other interventions implemented alongside this program/policy, if any
- How the components, subcomponents are achieved (i.e. how are they implemented)
- Who is in charge of the program
- What are the program benchmarks, who is monitoring that these benchmarks are met, what is the timeframe that they are supposed to be met in?
  1. **Monitoring and Evaluation of Implementation:** Include an overview of the M&E plan for implementation, who was in charge of it, any findings from the M&E.
  2. **Funding:** 3 – 4 sentences that includes:
- Information on the source of funding (where it came from)
- How much funding (how much money was donated and over what time period, was it the same annually or did it vary by phase or year)
- How long was the funding sustained (was funding consistent over the entire period or did the source change)
  1. **Success Factors/Barriers**: Include any details provided in program/policy reports, evaluations or from qualitative/stakeholder interviews.

## Supplementary Table 8 Detailed timeline of nutrition-specific and -sensitive laws, policies and programs in Kyrgyz Republic (1990-2017)

| **ACTS/LAWS/REGULATIONS** | | | | |  |
| --- | --- | --- | --- | --- | --- |
| 1. State Benefits Law   (1991-present) | | Description | The objective of the law was to reduce poverty among extremely poor families, families of children with disabilities, and the elderly (31). This law resulted in the establishment of two cash transfer programs: the Universal Monthly Benefit (UMB) and the Monthly Social Benefit (MSB) (27,31,62). The purpose of the UMB was to act as a safety net for impoverished families with children. While the MSB is a cash income replacement program that targets disadvantaged groups (27,31,62). | |  |
|  |  | Importance | Likely important | |  |
| 1. Order N 19: protection of breastfeeding   (1996-present) | | Description | The law was established from a UNICEF/WHO advocacy campaign. This law was implemented to the increase the proportion of mothers who breastfed their children and also sought to improve perinatal care. In this law, community health workers disseminate information about breast feeding to the community with a focus on pregnant women and women of reproductive age. This policy stressed the importance of breastfeeding, starting to breastfeed immediately after birth, and the timing and duration of breastfeeding (36). | |  |
|  |  | Importance | Likely important | |  |
| 1. Protection of breastfeeding of children and regulation of marketing of infant food - law   (2008-present) | | Description | This law aims to protect the health of infants and young children by promoting good breastfeeding practices. There was a need for this law as medical professionals were promoting the sale of breastmilk substitutes . The law ensures that good breastfeeding practices are taught, while regulating the marketing of breastmilk substitutes and related artificial nutrition products. These products have strict labelling requirements and are prohibited from including graphics, photographs, and drawings as a marketing strategy. These products must also state the benefits of breastfeeding, the expiry date, and name of the producer on the label (35,36). This law led to the integration of the WHO/UNICEF Baby-Friendly Hospital Initiative’s development of an Eleventh Step in the BFHI in 2009. This program discussed in the forthcoming section focused on adherence to indicators on the prevalence of exclusive breastfeeding into routine evaluations of standard practices at health facilities (36). | |  |
|  |  | Importance | Likely important | |  |
| 1. Food Security - law   (2008-present) | | Description | The government of Kyrgyz Republic adopted a law to ensure food security. This Food Safety law was introduced by the Government of Kyrgyz Republic after the inflation of food prices in 2008. The aim of the law was to ensure food accessibility and to improve food security and to ensure minimum consumption standards of food were met. The minimum standards of food consumption included domestic wheat procurement and distribution of wheat flour to the food-insecure population, building strategic food stocks, providing credit subsidies to farmers, introducing seasonal duties on wheat and wheat flour exports, supporting the use of agricultural machinery; and improving seed production (88). It led to the establishment of the food security and nutrition program (88). | |  |
|  |  | Importance | Insufficient evidence | |  |
| 1. Fortification of baking flour- law (2009-present) | | Description | This law is the legal framework for the fortification of flour with vitamin and minerals with the goal being to reduce the prevalence of anemia and other micronutrient deficiencies. The law and technical regulations apply to the production, storage, transportation, sale and recycling of wheat flour (89,90). All flour producers in the country were instructed to fortify the highest and first grade flours with vitamins and minerals that have been approved by local health agencies (91). The mandatory fortificants included in the law are Vitamins B_1_, B_2_, B_3_, folic acid, and the minerals iron and zinc. In 2012, there was a technical regulation for this law that included packing and labelling requirements, safety requirements, and storage and transportation guidelines. Included in this technical regulation are ramifications for failure to adhere to these requirements (89,90). | |  |
|  |  | Importance | Likely not important | |  |
| 1. Healthy children at the level of primary health care (regulation 589)   (2010-present) | | Description | The law was established by the Government of Kyrgyz Republic to build on existing legislation and improve the availability of health services for pregnant women and young children. The order includes the classification of childhood periods, monitoring child health from 0-17 years of age, instructions to assess the development of children 0-6 years and instructions on breastfeeding and complementary feeding until two years of age. The aim is to provide assessment of a child’s nutritional status as a preventative measure (41). Provides clear guidance on well child visits, which include nutrition, physical and psychological development, as well as care for the development of children aged 0 to 7 years (42). At present most monitoring of child development is conducted by health providers at primary care facilities (42). | |  |
|  |  | Importance | Insufficient evidence | |  |
| **POLICIES/STRATEGIES/PLANS** | | | | | |
| 1. Agrarian Land reform   (1991-present) | Description | | | In Kyrgyz Republic 85% of the agricultural land is composed of pastures and is used for grazing animals while 15% of the land is arable for crop growth. The land reform policies in Kyrgyz Republic have gone through three phases since establishment. Phase one occurred following the collapse of the Soviet Union between 1991-1993. During this phase the law of the land reform was passed. This legislation aimed to make 75% of the arable land private (16). The law of the land reform was the initiation of the distribution of arable land and agriculture to rural citizens. The second phase of the land reform was between 1994-2004 during this phase there was a mass distribution of transferable land, with a large quantity of distributions taking place before 2006. In 1998, there was a referendum which led to a Presidential decree on private land ownership. This decree transformed all land use certificates into land ownership certificates. The third and final phase began in 2004. During this phase there was a focus on registration, credit cooperatives, and insurance (16,24). | |
|  | Importance | | | Likely important | |
| 1. Prophylaxis of Iodine Diseases Plan   (1994-present) | Description | | | This policy was implemented to reduce the high prevalence of iodine deficiency, and iodine deficiency diseases (92). The prevalence of iodine deficiency was found to be high, and the goal was to protect the intellectual potential of children and to reduce the burden of iodine deficiency diseases. The policy aims to accomplish this through the fortification of salt with iodine. There was a technical regulation for this policy that came into practice in 2012. This regulation ensures the safety of salt iodization, and standardized the methodology for fortification. There were implementation barriers, including the sale of non-iodized salt imported from other countries and issues with fortification in country. This was a national program and was intended to reach rural and urban citizens of the Kyrgyz Republic (93). The number of households consuming adequately iodized salt improved from 27% in 1995 to 76% in 2005 (73,92–98). By 2014, 96.6% of households were consuming adequately iodized salt (73). Unfortunately, the literature from Kyrgyz Republic does not assess the impact of salt iodization on nutritional outcomes such as goiter or cretinism. | |
|  | Importance | | | Likely not important | |
| 1. “Healthy Nation” State plan   (1994-2000) | Description | | | This policy was the first health policy of Kyrgyz Republic. This led to the implementation of a comprehensive health reform in Kyrgyz Republic. An important component of these reforms was to downscale the health care system from its soviet levels. As the soviet design of health care was not affordable for Kyrgyz Republic as an independent country. Some of the key components of this policy was to provide free food for children from low-income families, providing pregnant women with vitamins, promotion of breastfeeding, and improving drinking water sources. The goal of Health Nation is to build a foundation for a healthy lifestyle by improving physical and spiritual development. The program aimed to reduce the rate of TB, disability, under-5 morality, and maternal mortality. Health Nation laid the foundation for the establishment of reforming the primary head care system and led to the establishment of Manas (38,39). | |
|  | Importance | | | Likely important | |
| 1. National Health Reform Plan “Manas”   (1996-2006) | Description | | | Manas was a reform of the health care delivery system. The aim of Manas was to strengthen the primary health care system, to develop family medicine and to restructure the hospital sector. The health sector reform improved the provision of pharmaceuticals, the quality of programs for maternal and child health, cardiovascular disease, TB, and HIV/AIDS. A major component of Manas was refinancing health financing and the introduction of outcome based payment methods. Manas became an internationally recognized model for health financing (20,43–45,99). The Manas program went on from 1996 – 2006. This health reform was successful in part due to the comprehensive approach rather than many different working pieces (15). This program was reformed in 2006 when the government approved the Manas Taalimi National Health Care Reform Programme (2006-2010) (43). | |
|  | Importance | | | Likely important | |
| 1. National Poverty Reduction Plan “Araket”   (1998-2005) | Description | | | This was first attempt of the government to undertake a comprehensive set of measures aimed at poverty reduction. Araket was a poverty reduction strategy, aimed at reducing poverty rates and stabilizing the national economy. There were two stages to the program. The first stage was from 1998-2005 which sought to stabilize the national economy, ensuring its growth and to prevent the expansion of poverty by reducing its most acute forms. Poverty reduction was expected to continue into the second stage of the program to build upon the economic growth and benefit all members of the population (40). The program laid the foundation for poverty alleviation and supported poor households. This policy incorporated the creation of an efficient system of microcrediting as well as additional incentives for the unemployed, and provided support to the poor and disabled (40). Measures were incorporated to ensure the targeting of benefits was made for low-income families (100 soms per person). The policy also created social and labor protection and employment opportunities in the country (40). Water and sewage costs were reduced, unemployment benefits were provided, and there was an increase in the average size of pensions through this policy (40,100). | |
|  | Importance | | | Likely important | |
| 1. National Policy of Food Security   (1999–2007) | Description | | | The aim of this policy was to improve food security for all citizens of Kyrgyz Republic by means of providing constant access to a sufficient number of food products in all regions. This policy focused on social protection and benefits for poor households and agriculture development. This policy was developed and approved by the Government of Kyrgyz Republic and by the European Economic Commission to decrease the prevalence of food insecurity (9,11). The policy came into practice alongside the inflation of food prices, without the corresponding increase in income. There were regulations in place to ensure food security. The policy emphasized the provision of social protection and health services. In addition, it improved access to credit, strengthened agricultural processing, and ensured the proper use of natural resources (9,11). Unfortunately, this policy was limited by insufficient resources, and therefore did not make a major difference in food security. | |
|  | Importance | | | Likely not important | |
| 1. Comprehensive Development Framework of the Kyrgyz Republic   (2002-2010) | Description | | | The comprehensive development framework (CDF) is a World Bank initiative and in Kyrgyz Republic was a long-term development strategy that focused on social and economic development. The goal of the CDF was to reduce poverty rates in Kyrgyz Republic by 50% by 2010 (101). This framework was created to align with the millennium development goals. The program ensures that a fair society is built with sustainable economic growth. Other focuses on the CDF were to promote a healthy lifestyle, improving access to safe drinking water, and strengthening the capacity of health and medical workers. In addition, a major component to this framework was the community-based early childhood development project. This focused on child health and nutrition, supporting early childcare and education through kindergartens and pre-schools (23,101,102). Even though the CDF ran between 2002 – 2010, the implementation was interrupted after the tulip revolution and change in government in 2005. | |
|  | Importance | | | Likely important | |
| 1. National Health Care Reform Plan “Manas Taalimi”   (2006-2010) | Description | | | This health care reform aimed to achieve the millennium development goals (MDGs) by addressing key health issues. The program was introduced to ensure active involvement of the population and emphasized reducing the financial burden for patients (43). The primary objective of the policy was to improve the delivery system of healthcare and increase access to high quality medical care for the population at all levels of the delivery system (43). Many components of Manas Taalimi are the same as those of the Manas policy. However, Manas Taalimi aimed to build on the results achieved through the Manas program to ensure more active involvement of the community. Some of the similar objectives were to improve the quality of health services and strengthen public health, and improve the quality of education (43,45). The different objectives of Manas Taalimi included solidifying the health financing reforms, increasing the effectiveness of primary care, and improving access to specialized care by reducing the financial burden on patients (5,12,20,43–45). For example interventions for mothers included promoting reproductive health and family planning, seeking skilled care for birth, screening of UTI and UTI management, and basic obstetric care. For neonates, interventions included under this policy were immediate breastfeeding, exclusive breastfeeding, routine postnatal care and care of low birth weight infants, neonatal resuscitation, vitamin A supplementation, expanded program for immunizations (including new vaccines – Haemophilus influenze type B, pneumococcal, and rotavirus), injectable antibiotics for sepsis, oral antibiotics for pneumonia (5,12,20,43–45). | |
|  | Importance | | | Likely important | |
| 1. Country Development Strategy   (2007-2010) | Description | | | The Country Development Strategy is a similar program to the National Poverty Reduction Strategy. The overall goal of the CDS is to improve the level and quality of life of citizens by ensuring sustainable economic growth, creating conditions for full employment, high and sustainable income, and access to a wide range of social services, and compliance high standards of living for health in environment (103). In order to further promote the reforms within the framework of the implementation of the NPRS, attention was focused on three main areas: (i) strengthening economic growth and creating jobs, (ii) improving the efficiency and targeting of social protection and human development, and (iii) improving governance, sector and institutional and human capacities. Measures to ensure affordable and quality education were: increase the share of public expenditure on health to 13% by 2010, the introduction of human resources management mechanisms that promote the involvement of medical workers to rural areas, improve the quality of medical education, introduction of world practices (103). | |
|  | Importance | | | Insufficient evidence | |
| 1. National Health Reform Plan ”Den Sooluk”   (2006-2010) | Description | | | This national healthcare reform program focuses on improving the health outcomes of common diseases in Kyrgyz Republic. Den Sooluk is based on three interrelated pillars including expected health gain, improving core services needed to achieve expected health gains, and removing barriers that prevent the delivery of core services and health improvements. The priority health improvement areas included maternal and child health, cardiovascular disease, TB, and HIV. The program focuses on improving social determinants of health and is based off both successes and shortcomings of the Manas and Manas Taalimi programs (13,44,46,47). Den Sooluk aims to ensure universal coverage of the population regardless of social status, gender, and insurance with proper high quality health care services, sanitation and other preventative services (44). There were four priority health improvement areas with population interventions. These four areas are (i) Maternal and newborn health (47) (ii) Cardiovascular disease (47) (iii) Child health (47) (iv) strengthening the inter-sectoral approach to public health (47). | |
|  | Importance | | | Likely important | |
| 1. Food Security and Nutrition Plan   (2015-2017) | Description | | | The goal of the program is to address food insecurity in Kyrgyz Republic. The program aligns with the global concept of food security and was in response to the inflation of food prices locally. There are four platforms such as food availability, accessibility, improving dietary quality and diversity, and ensuring food safety. This program allowed for a departure from traditional tools for managing food security and nutrition issues in the country and allowed for an expansion of food security with the target of improving nutritional quality and the overall health of the population (13,53,73)*.* This introduces a nutrition as a policy area, but not much has been done in terms of practical implementation. | |
|  | Importance | | | Insufficient evidence | |

| **PROGRAMS/PROJECTS** | | |
| --- | --- | --- |
| 1. Conditional Cash Transfer Program : Universal Monthly Benefit   (1995-present) | Description | The Universal Monthly Benefit (UMB) was introduced in 1995 as an integral part in reforming social safety nets. The purpose of the UMB was to provide aid targeted to children in impoverished families (27). This safety net targets children aged 1.5-16 years (or up to 21 years if they are still a student in a low-income family). The number of children in a family has been proven to be significantly correlated with poverty. Therefore, the UMB’s focus on children made the benefit well targeted. Despite this, the amount of the benefit was low. Data shows that 54% of the total benefits went to the poorest quintile. The UMB was only able to provide coverage for 20% of the poorest population quintile and 14% of the second poorest quintile (27). |
|  | Importance | Likely important |
| 1. Conditional Cash Transfer Program : Monthly Social Benefit   (1998-present) | Description | Established in 1998 as a cash income-replacement program that is categorically targeted to disadvantaged groups. This is a cash income replacement program. The Monthly Social Benefit (MSB) targets disadvantaged populations such as children with disabilities up to 18 years of age, other people with disabilities, orphaned children, mothers of large families, and elderly people who don’t qualify for pensions (27). In 2011 there was an increase in the MSB by 100-300 soms per month in 2008 and many extremely poor families received an increase in the benefits they received in 2011 (31). Data shows that the MSB was only able to target roughly 50% of low-income households to receive benefits (31). The total MSB rate is 1000 – 2000 soms per month (27). |
|  | Importance | Likely important |
| 1. Program on Prevention of Iron Deficiency Anemia among the population of the Kyrgyz Republic   (1995-2016) | Description | This program is designed to prevent anemia through multiple different internationally accepted interventions. These interventions include fortification of flour, preventative supplements for at risk groups, the promotion of dietary diversity, minimizing and controlling the prevalence of infection, the communication of monitoring and research, and linking anemia control to public health services. The majority of work on anemia prevention and control in Kyrgyz Republic was focused on increasing oral supplementation as a preventive measure for women of childbearing age and children 6 to 24 months of age. There were multiple initiatives including food fortification, providing iron and folic acid supplements to at risk groups, promoting dietary diversity, control of infection, improving public health response to anemia, and improving communication monitoring and research (104). |
|  | Importance | Likely not important |
| 1. Mandatory Health Insurance (HFI) Fund (MHIF)   (1997-present) | Description | The Kyrgyz Mandatory Health Insurance Fund (MHIF) program was introduced to increase health system resources through pooling of public funds, including general taxation and payroll taxes earmarked for mandatory health insurance. Health financing reforms introduced a purchaser-provider split, and a single-payer health system. The introduction of the MHIF was critical to funding and providing the essential package of health services (SGBP). Copayments improved funding at local health facilities for drugs, medical supplies and aimed to decrease informal, out-of-pocket payments among individuals. This program was a key component of the broader *Manas* health sector reform. The cost of Health Insurance could be purchased for 400 soms/month and equates to approximately $10 USD for those who are unemployed (14). Another goal of this program was to improve the use of primary care. This was achieved by the patient requiring a referral from a physician in an family group practice. Without a referral, the patient must pay for the full cost of their services, the aim of this is to improve utilization of primary care (14). |
|  | Importance | Likely important |
| 1. Primary Health Care Reforms & Introduction of Family Medicine Model   (1997-present) | Description | This reform changed the provision of health services from a centrally managed specialist-based primary care that was in place during Soviet ruling to an approach that employs a comprehensive family medicine model (105). This involved training a cadre of mid-level general practitioners to provide primary health care at the community level, a substantial gap in the post-Soviet health system. Family medical centres and family group practices were established (105). Kyrgyz Republic is recognized as a regional leader in family medicine reform (106). |
|  | Importance | Likely important |
| 1. State Guaranteed Benefits Package & Additional Drug Package   (2000-present) | Description | The objectives of the State Guaranteed Benefits Package (SGBP) & Additional Drug Package (ADP) were to introduce a co-payment for health services. The SGBP defines the package of essential health services provided to citizens free of charge regardless of insurance status and enrolment (107). In addition the SGBP introduced co-payments for certain services were introduced to replace informal charges and out-of-pocket expenditures. The objective of the ADP was to insure population at the outpatient level to improve affordability and accessibility of select drugs for the population (108). These programs were funded through the MHIF and helped to decrease informal and out-of-pocket payments, and increased accessibility and utilization of primary care services among the population. The SGBP is seen as the critical step/strategy of Manas health sector reforms and has contributed towards sustaining universal health coverage. The way the program was implemented was that MHIF contracts pharmacies to deliver drugs included in a predetermined list. Pharmacies are reimbursed for the cost of the generic version of the drugs. If the pharmacy charges more for a drug, the patient must pay the difference if the cost of the drug supplied by the pharmacy is higher that the agreed upon price from the MHIF (14). Only citizens enrolled in the MHI and at a family group practice can receive the ADP benefits. |
|  | Importance | Likely important |
| 1. Baby Friendly Hospital Initiative   (2000-present) | Description | The program regulates the implementation of the 10 steps to successful breastfeeding and accredits hospitals which implement these breastfeeding principles (36). The program is in maternal hospitals and at primary health care centers. It is a program of the Ministry of Health. In addition to implementing the 10 principles of successful breastfeeding, the program the program also regulates the organization of a dairy room to store pasteurized milk in maternity hospitals and describes acceptable indicators to supplementary feeding (109). This was reformed in 2009 to include an 11^th^ step. The 11^th^ step prohibited the use of breastmilk substitutes. |
|  | Importance | Likely important |
| 1. Establishment of Village Health Committees & Community Action for Health Program   (2001-present) | Description | The Village Health Committees (VHC) were established to improve health promotion, provision of preventive health services, and to increase local/community ownership over health. Encourage community action for health through large community-based health development initiative (20). By 2011 there were 1700 VHCs which covered 84% of all villages. The volunteers are trained by health providers, and are responsible for disease prevention and health promotion, particularly exclusive breastfeeding. This programme aimed to improve population and community involvement (a key component of the Manas Taalimi reform) and is recognized by the Ministry of Health as the main strategy for community mobilization and health promotion by the Ministry of Health (20). |
|  | Importance | Likely important |
| 1. “Jan-Ene” Program   (2002-2006) | Description | This program focuses on promoting perinatal care, gender equality, and improving the genetic pool. This program will establish this by improving the reproductive health of girls, women, and to reduce infant mortality, and maternal mortality. In addition, it will reduce the number of abortions by improving modern family planning methods. This program focused on improving the training and professional development of medical personnel and provide public awareness on safe motherhood and women’s health (110). |
|  | Importance | Insufficient evidence |
| 1. The National Poverty Reduction Strategy (NPRS)   (2003-2005) | Description | The National Poverty Reduction Strategy (NPRS) is the first stage in the implementation of the Comprehensive Development Framework of the Kyrgyz Republic until 2010 (CDF).  There were four main components of the program which included (i) enhancing economic growth and job creation, (ii) improving the efficiency and targeting of social protection and human development, and (iii) improving governance, public sector efficiency, and institutional and human capacity. During the implementation of the NPRS, reforms were carried out in a number of areas. This ensured macroeconomic stability and resulted in an average annual economic growth of 5%, incomes of the population increased, health and education services improved, which led to a poverty reduction from 52% to 44% (40,100,111). |
|  | Importance | Likely important |
| 1. Vitamin A supplementation for children   (2005-2011) | Description | This program was introduced to reduce the morbidity and mortality of children aged 6 months to 5 years. This was accomplished through a bi-annual delivery of Vitamin A supplements. Monitoring suggest that coverage of Vitamin A supplementation among children was 98%. Data showed that this program effectively reduced the prevalence of Vitamin A deficiency by the year 2010 to 5% (28,49). |
|  | Importance | Likely not important |
| 1. Integrated Management of Childhood Illness (IMCI) program   (2005-present) | Description | The aim of the program is to improve the medical supervision of sick children aged 2 months to 5 years to reduce the rate of child and infant mortality by increasing the level of maternal and child care. IMCI was implemented at the primary care level in Kyrgyz Republic. The implementation was then evaluated by the WHO. The program includes the care for children and improving child development. The program targets pre and post medical school graduates with the focus of improving the knowledge translation in child care therapies for medical professionals. IMCI had given medical professionals greater confidence in handling children under 5 years and more systematic knowledge, which gave them greater confidence in the therapies provided by their institutes; they had begun to devote more time directly to the child while filling in their records; parents had begun to bring their children in for treatment earlier in their illness and thus presenting less seriously ill children; parents were asking for children to be weighed; fewer unnecessary medicines were being used; mothers received more advice on how to nurse a sick child and care for a healthy one; mothers liked the new ways in which children were received; more mothers were breastfeeding long term, and more children were receiving only breast-milk; fewer children were being referred for hospitalization (48). There was also a focus on improving practices at home for mothers to bring sick children in for treatment earlier and to use fewer unnecessary medicines (48). |
|  | Importance | Likely important |
| 1. Gulazyk Supplementation   (2009-present) | Description | Gulazyk is a home fortification intervention to deliver micronutrients to children aged 6-24 months. In English, Gulazyk translates from Russian to a meat product rich in energy and nutrients. The program was initiated by the Ministry of Health in Kyrgyz Republic and by UNICEF in response to the high burden of anemia in young children. The powder contains iron, zinc, Vitamin A and C and was distributed by trained primary care providers at family health clinics (53,75,112). |
|  | Importance | Likely not important |
| 1. Quality Health Care Project   (2010-2015) | Description | This was a five year program that aimed to improve the health status of the population in Central Asia by improving the public health system, health system management, health services, and educating communities on the need to respond to health concerns. The program provides training, and equipment to improve management, financing, and implementation of health services related to the 5 public health areas of focus (113). The 5 components were considered to be the most pressing health issues at a national level in Kyrgyz Republic at this time. These issues included TB prevention and treatment; HIV prevention and access to care; improvements to maternal and child health (MCH); increased use of family planning (FP) and reproductive health (RH) services; and addressing anti-microbial resistance addressing other public health threats (OPHT) (114). The WHO effective perinatal care program reached all maternity and delivery facilities in Kyrgyz Republic (115). The three main components were TB, HIV, and Maternal and Child Health (113–118). |
|  | Importance | Insufficient evidence |

# Supplementary Appendix 7 Qualitative Results

**Qualitative Inquiry**

Results from the in-depth interviews and focus group discussions are organized according to type of stakeholder, by national and community-level perspectives. Results are summarized according to key themes including basic (contextual) factors, nutrition-specific and –sensitive policies and programs, intermediate and immediate causes. Supporting evidence and quotes were selected to demonstrate a range of participants’ diverse perspectives on the determinants, and policies and programs.

**National Expert Stakeholders**

In-depth interviews were conducted with 20 national key informants and stakeholders working in the health and nutrition sectors (Table 1). These State and non-State actors were identified and recruited based on their substantial expertise and experience working in nutrition-specific and –sensitive sectors, institutions and programs in Senegal and include multilateral, bilateral, government (national and subnational), academic/research, as well as local/international non-governmental organizations. Key informants’ responses were categorized into several major drivers based on our conceptual framework including basic or distal causes, nutrition-specific or sensitive programs, intermediate causes, and immediate or proximate causes.

*In-depth interviews*

In-depth interviews were conducted with national stakeholders in Bishkek and included nutrition experts, representatives from multi- and bilateral international donor organizations, representatives of related ministries (Ministry of Health, Ministry of Health, Ministry of Social Protection, Ministry of Agriculture), and state agencies (National Center for Maternity and Childhood Protection). A list of nutrition-specific and –sensitives events (policies/strategies/laws/programs) in Kyrgyzstan was shared and respondents were asked to describe the interventions, contexts, key stakeholders, as well as successful factors and barriers to implementation. In-depth interviews with national stakeholders also aimed to understand key trends in child undernutrition in Kyrgyzstan over time and to discuss contextual factors that represent enablers or drivers of change in child malnutrition indicators, including socioeconomic and lifestyle determinants.

In-depth interviews were also conducted at community level with young childcare workers in two oblasts (Talas and Batken) to outline community-level perspectives and experiences on transitions in nutrition and stunting in Kyrgyzstan. These interviews explored how contextual factors including changes in the social and economic situation, access to key resources (e.g., water/sanitation, health services), and changes in dietary intake were experienced at the community level. Further, key recommendations for pregnancy, breastfeeding and child nutrition were identified, and stakeholders evaluated changes in local water/sanitation/hygiene practices. In addition, key nutrition-specific and –sensitive events that community representatives felt are responsible for driving stunting reductions were identified and explored potential facilitators and challenges for implementation from a community, grassroots perspective.

## Supplementary Table 9: Summary of in-depth interview national respondents

| **Participant** | **Position & affiliation** |
| --- | --- |
| **In-depth interviews participants** | |
| Participant 1 | Head of Food Security and Agromarketing Department, Ministry of Agriculture |
| Participant 2 | Head Doctor, the Center for State Sanitary and Epidemiological Surveillance of Bishkek, former ADB's Project Manager for Nutrition |
| Participant 3 | Senior Health Specialist, World Bank |
| Participant 4 | Head of Public Health Department, Ministry of Health |
| Participant 5 | Department of Disease Prevention and State Sanitary and Epidemiological Surveillance |
| Participant 6 | Head of the Development of Social Services for Persons with Disabilities and Senior Citizens Department, Ministry of Labor and Social Development |
| Participant 7 | Health and Nutrition Officer, UNICEF |
| Participant 8 | Head of the Nutrition Department of the National Center for Maternity and Childhood Protection under the Ministry of Health |
| Participant 9 | Director, Republican Center for Health Promotion |
| Participant 10 | Chair of the Executive Committee Alliance of Civil Society for Nutrition and Food Security, SUN movement |
| Participant 11 | Director, Association of Suppliers |
| Participant 12 | “Spring” Project, USAID |
| Participant 13 | National Center for Maternity and Childhood Protection |
| Participant 14 | Chairman of the Mandatory Medical Insurance Fund under the Government of the Kyrgyz Republic, former Deputy Minister of Health |
| Participant 15 | Director of Programs, MercyCorp |
| Participant 16 | Programme Policy Officer (Nutrition), World Food Program |
| Participant 17 | Professor of the Kyrgyz-Slavic Russian University, former Main Endocrinologist of the Ministry of Health |
| Participant 18 | Kyrgyz-Swiss Health Reform Support Project, Swiss Cooperation |
| Participant 19 | Nutrition expert |
| Participant 20 | General Director, Association of Village Health Committees |

**Basic Causes**

***Socioeconomic Indicators***

After the USSR collapse in 1991, all former countries of the Soviet Union experienced a decline in the national economy, and increased poverty levels. The majority of national key informants reported that socioeconomic indicators have improved in Kyrgyzstan over the last 20 years, and that the rate of poverty has decreased. Further, improved quality and standard of life was highlighted, where respondents emphasized improvements in the access to basic necessities, including food security.

*“[The] USSR collapse led to the overall situation worsening - devaluation of the ruble and reforms. There was a very unstable, turbulent period, and this all had a negative impact on the economic situation [and] families.” (Participant #12, USAID)*

*“According to the World Bank in Kyrgyzstan, there are improvements in the poverty situation.” (Participant #4, Ministry of Health)*

*“The country has moved from a low-income category to a middle-income country, even this classification shows that we are no longer beggars, but "medium" already, the volume of consumed products has changed for the better.” (Participant #14, Mandatory Medical Insurance Fund)*

*“The economic situation in 1997 was worse, but I think that situation has changed due to activities of the government and donors.” (Participant #15, MercyCorp)*

*“[The] situation with chronic malnutrition seems to [have] improved, as the standard of living of the population has risen.” (Participant #1, Ministry of Agriculture)*

*“[The] situation with nutrition has improved, looking at the stunting indicators in DHS in 2012 it was 18%, in 1997 this figure exceeded the current almost threefold.” (Participant #15, MercyCorp)*

***Gross Domestic Product (GDP)***

According to the World Bank for the first ten years, the country's GDP was halved from $2.674 billion to $1.249 billion USD. It took almost a decade so that new market institutions could be established and private entrepreneurs became the main driver of the country's economic development. Since 2000, there has been a steady economic growth and despite two periods of political instability, by 2013 Kyrgyzstan’s GDP increased to $7.226 billion USD. However, according to the International Monetary Fund (IMF), Kyrgyzstan still ranks 153rd in the world in terms of GDP per capita, with an indicator of US $1113 (119). Significant regional disparities in economic growth and the reduction of poverty are experienced. The highest levels of poverty are observed in Naryn and Batken oblasts, at 37.8% and 37% respectively. The situation in Jalal-Abad oblast is a little better, with a poverty rate of 32.2%. Despite the close proximity to the capital, where the poverty rate is only 9.8%, in Chui oblast the poverty rate is very high at 30.3% (120). The largest number of registered individuals that are currently unemployed is observed in the South of the Republic. In Jalal-Abad oblast there are 17,280 people, in Osh and 11,878 registered as unemployed. Of the total number of registered unemployed, 51.1% were women. The level of registered unemployment was 2.3% of the economically active population.

*“The situation is improving in principle, according to the latest MICS data - 13% of children under 5 years old, who suffer from stunting. In principle, 13% is not bad compared to other countries, but an aggravating factor is that the distribution of this indicator within the country is uneven. If we compare the regions by the highest index, then it is in Jalal-Abad - 21%, which is comparable with African countries” (Participant # 16, WFP).*

***Migration***

Internal and external migration was identified as a barrier or limitation to access to health services, and social and economic indicators. Further, limited data is available to adequately measure and understand the extent of internal migration in the Kyrgyz republic.

*“[During] the last 20 years, internal migration has intensified, due to the citizens of rural areas who moved to new housing developments in Bishkek that do not have access to social services, a health program. One cannot say that all are not registered, but more than 50% of the population informally lives there, they are not registered, accordingly, they have problems with access to health services. Even if we want to get some specific data, we are faced with the fact that we cannot get these data. The health system, such as primary levels (Feldsher-Midwife stations, Family Doctors Groups), is trying to cover, but they are based on information impact, and state statistics cannot directly provide it. People who live here [in Bishkek] physically are documented as they living in regions. And this migration is problematic. These new housing developments are occupied by young families, who have small children, and because of this social welfare has worsened.” (Participant #7, UNICEF)*

**Basic Causes: Nutrition specific- and -sensitive programs/policies**

According to interviews with key country experts, the most influential policies and programs that contributed to the reduction of stunting include: breastfeeding promotion policies and programs, micronutrient supplementation (Micronutrient Power or “Gulazyk” and vitamin A), salt iodization, flour fortification, Integrated Management of Childhood Illness (IMCI), and programs that provided benefits for low-income families.

*Breastfeeding promotion*

Exclusive breastfeeding promotion represents a key intervention identified in the literature to reduce stunting. Substantial gains of exclusive breastfeeding of infants under six months of age have been achieved in Kyrgyzstan, from 32% in 2006 to 41% in 2014 (UNICEF, 2017). In addition, 69.5% of infants are predominantly breastfed. Despite this progress, Kyrgyzstan continues to experience some of the world’s lowest rates of breastfeeding. Initial commitments and efforts to promote exclusive breastfeeding involved Kyrgyzstan’s ratification of the United National Convention on Rights of the Child (CRC), Decree #1402 – XII, January 12, 1994 (121). Article 24 of the CRC states that Kyrgyz Government will take the necessary measures to provide awareness to all sectors of society, particularly parents and children, regarding the health and nutrition of children, the benefits of breastfeeding, hygiene, sanitation of the child's environment, prevention of accidents, access to education and to support the use and application of this knowledge (121). Secondly, on January 16, 1996 Order #19 was accepted by the Ministry of Health “On the Work Organization of Maternity Hospitals (Departments) and the Introduction of Exclusive Breastfeeding in Maternity Hospitals in the Kyrgyz Republic”. The Order called for the introduction of exclusive breastfeeding (without giving water and other liquids) from the moment of the birth up to 6 months of life as the main task in the activity of obstetric institutions. The main purpose of the Order was further improvement of work of maternity hospitals (departments) and their reorientation to Baby-Friendly hospitals (hospitals, friendly to the child). The first hospital accredited with principles of BFHI was registered in 2000 (36).

*“This program [breastfeeding promotion] was also launched jointly with UNICEF, WHO in the early 1990s. At the first stage, it was the introduction of a baby-friendly hospital initiative (BFHI). It started with the introduction of this policy, i.e. in maternity hospitals, where the joint stay of the child with his mother in one room was introduced, so that they were applied to the breast and fed at the child's request, and not by the hour. Initiative groups were created that provided support to mothers after discharge from the hospital.” (Participant # 19, Nutrition Expert)*

Key informants emphasized that Kyrgyzstan’s commitments to international and national treaties and policies have positive influenced exclusive breastfeeding among infants. Certification of hospital institutions to be ‘baby-friendly’, ensuring supportive work environments for employed women to continue breastfeeding and annual public awareness campaigns were identified as key factors that increased opportunities and support for exclusive breastfeeding among the population. Further, efforts to improve practices in hospitals, training of health personnel and keeping mother and infant together in the hospital, have led to early initiation, and increased uptake and support for breastfeeding including training of health personnel.

*“In 2007, serious work was carried out to write a law on breastfeeding promotion, and this was the first excellent law in the Central Asian region. Due to our capabilities, we talked about the administrative responsibility of ministries, creating conditions for working mothers, especially in public institutions. The law was successfully passed. Once a year, when recertification passes, we issue a certificate, which is posted near the entrance to the building that this building corresponds to the child-friendly hospital.” (Participant #7, UNICEF)*

*“Now the responsibility for this program was taken by the Ministry of Health. Once a year, in October, the campaign on promoting public awareness about exclusive breastfeeding is conducted by community volunteers from various organizations. The main activities are informing the population and training medical personnel, introducing rooming-in [joint stay of mother and newborn] of mother and child and early initiation of breastfeeding. The main results [find that] 40% of children are exclusively breastfed. It shows that quite good work has been done, but not yet to the end.” (Participant #15, Mercy Corp)*

*“The program on breastfeeding promotion has been going on since the year 2000 and is connected with the introduction of the Initiative of the Hospital’s Child-Friendly Attitude (IHCHA), later also to mothers. Thanks to this program, right now after childbirth the child is next to his/her mother. 10 steps of this program are all carried out, [the] 11th step is “Not to accept gifts”, was adopted along with the law on breastfeeding (2008). To date, 42% of maternity hospitals belong to IHCHA, where all these 11 steps of successful breastfeeding are performed. Since 2015, IHCHA is being introduced into the FMC [Family Medicine Centers]. Now we recommend exclusive breastfeeding for up to 6 months and breastfeeding up to 2 years.” (Participant #8, National Center for Maternity and Childhood Protection, National Center for Maternity and Childhood Protection)*

*“UNICEF had a program on breastfeeding promotion at the maternity ward level. In another project, we were faced with the fact that programs are result-oriented, when we take random samples of mothers to find out what is actually going on between mothers and doctors, and we asked them, "what are three reasons why breastfeeding is important?" Thus, we checked whether the information was delivered to mothers by doctors, pediatricians. And this is done quarterly, and we see the result. Of course, doctors are motivated for this, they get points if their patients answer. We saw the inadequacy, the extent to which the training takes place. The information about the breastfeeding went through four channels: 1 - through local communities, 2 - through primary sources, when mothers come for neonatal care, and there they say why it is important to breast-feed up to 3 years. The third channel is through the hospital-maternity home. Channel 4 is television and videos.” (Participant #3, World Bank)*

*“Therefore, from the first day of the birth of the child, he/she should be put on the mother's breast, that it is necessary to stimulate the consumption of the first mother’s milk (beestings/colostrum/foremilk), because this itself contributes to the active allocation of milk and the reduction of the uterus, and the prevention of uterine bleeding when the uterine tone is restored. The first feeding is also the prevention of this child not being abandoned, because it is unlikely that a woman who has fed her child several times, then will leave/give up him/her in the maternity house or injure him/her. The very fact that children are left in the maternity hospitals, and those who have not been breastfed and are afraid of the first feeding leave them. And a woman who is determined to throw the child for various reasons (social, migration), and they try not to touch him. But if we overcome this barrier, then, no matter how hard it is, the woman will not be able to abandon the child.” (Participant #14, Mandatory Medical Insurance Fund)*

*Challenges to promotion of breastfeeding*

Key experts described several barriers that continue to limit progress in breastfeeding and implementation of breastfeeding interventions and laws, which have contributed to poor indicators relating to exclusive breastfeeding. These challenges include milk substitutes and the power of companies that produce them, particularly attempts to promote their products through medical workers in maternity hospitals. Limited or poor implementation and lack of monitoring and accountability of breastfeeding laws and interventions were identified as barriers, as breastfeeding represents a multi- and cross-sectoral public health challenge. Inaccurate breastfeeding advice provided by grandmothers, mothers-in-law and some health care workers to new mothers, in line with traditional beliefs or customs under Soviet ruling, were identified as continuing challenges. In addition, some national stakeholders felt that promotion of breastfeeding was not necessary and that breastfeeding was universal particularly due to poverty. These misconceptions relating to breastfeeding progress, may threaten its prioritization and future progress in this area.

*“Barriers to breastfeeding are mixtures for children to sell them, producers promote their products through health workers, in maternity hospitals, as gifts to health workers, although there is a law. Now the law on breastfeeding is being revised and the responsibility for violating this law will be toughened.” (Participant #19, Nutrition Expert)*

*“One can meet some recommendations to women in childbirth from medical staff if there is no breast milk during their stay in maternity hospital like “give her/him water” or “give him/her a spoon of [black] tea” and etc.” (Participant #18, Swiss Project)*

*“The main barriers to the implementation of this program are migration, employment of mothers, misunderstanding of breastfeeding importance, advertising of artificial mixtures, old habits, because grandmothers try to feed small children with tail fat or give water.” (Participant #15, Mercy Corp)*

*“It is difficult to monitor this law. The producers of artificial mixtures are very rich companies, which have many resources, and alone one cannot fight with them. It is necessary to create mass pressure, protect the law, and monitor the implementation of the law all the time.” (Participant #10, SUN movement)*

*“This law [Breastfeeding Promotion] practically does not work. Because everyone should participate in the implementation of this law, not only the Ministry of Health. There is a simple example: in all institutions where women work, mothers’ and baby's rooms have to be opened. It is done for those who have small children but are forced to work; so they can bring their child there and calmly feed him/her. In many countries in public places there are such rooms, we still have such rooms only in airports. In the ministries and departments themselves, there are no such rooms.” (Participant #5, Sanitary and Epidemiological Surveillance)*

*“Promoting business of artificial mixtures, especially when advertising food, people get an imposed opinion that a child does not need to be breastfed, that it can replace it. They are divorced from reality, put too high knowledge, because a large number of useful substances that the child receives from the mother cannot be reserved and saved. Of course, a number of substances of proteins, carbohydrates, which are needed as a building material for the body, can still be preserved, but when there is no such complex, breastfeeding is more effective.” (Participant #14, Mandatory Medical Insurance Fund)*

*“Amendments to the law on breastfeeding promotion are needed, e.g., that in emergency situations humanitarian aid in the form of artificial nutrition should be distributed under the supervision of the Ministry of Health. Because in 2010 [during the conflict on the south], there was a massive flow of such humanitarian aid, and the delivery was uncontrolled.” (Participant #4, Ministry of Health)*

*“Promotion of breastfeeding is not particularly relevant for our region, because, I think breastfeeding is the main product for children, especially for the poor, because families do not have money to buy artificial powders.” (Participant #11, Association of Suppliers)*

*Micronutrient Powder “Gulazyk”*

The second important program according to experts, which had a positive influence on stunting reduction, was vitamin and mineral supplementation through the micronutrient sprinkles, “Gulazyk” initiative. In 2009, the Ministry of Health of the Kyrgyz Republic launched an Infant and Young Child Nutrition (IYCN) program. This involved the promotion of home fortification of complementary foods with a micronutrient powder (MNP), containing iron (12.5 mg elemental iron), vitamin A (300 µg), and other micronutrients. Every 2 months, children aged 6 to 23 months were provided 30 sachets of MNPs to be taken on a flexible schedule (71). The powder, Gulazyk in Kyrgyz and Sprinkles in English, has been distributed in Talas oblast since June 2009 and is part of a larger pilot program to fight malnutrition and promote early childhood development, implemented since 2008 by Kyrgyzstan's Ministry of Health, the UN Children's Fund (UNICEF) and the Swiss Red Cross, with support from the US Centre for Disease Control (CDC) (122).

Key informants felt that successful piloting of Gulazyk in Talas oblast and adequate sensitization of the population on the benefits of the MNP program helped to address public concerns and resistance and contributed to its uptake and use. Further, widespread coverage was achieved by support of donors, primarily UNICEF, and the provision of Gulazyk free of cost to the public. Targeting of low-income families and distribution through the health system were also factors that led to increased uptake and success of the program.

*“We were the first who initiated this project. It was named the Sprinkles, and was ordered from Nestle, Switzerland. Later, UNICEF started to work with this project and named it Gulazyk and now buys it from India.” (Participant #18, Swiss Project)*

*“In the early 2000s the Kyrgyz-Swiss Health Reform Support Project invited the developers of Gulazyk and they presented the results to us that this powder is added to food, during any meal. Then the Swiss project together with the Ministry of Health conducted a couple of pilots on Gulazyk, in these pilots the project worked well. Since 2009, UNICEF, the Swiss project, the Ministry of Health (coordinator) actively started to implement this program. This powder was purchased through UNICEF and distributed free of charge to children. The pilot project was in Talas region (since the MICS 2005 study showed a high level of stunting), they worked out the acceptability, ways of monitoring this program, etc. In Talas, there was almost 100% coverage, the actual coverage of about 90% (who consume Gulazyk regularly). Then when all the mechanisms were worked out, they went to Naryn oblast (the Swiss project). In Naryn, the program was not successfully implemented, because the main focus was on the Village Health Committees (informing the public). After Naryn (2010) UNICEF purchased the powder for the whole country (2011), throughout the country there was a 100% coverage with Gulazyk, but the actual coverage (who consumed Gulazyk) was about 70%”. (Participant #19, Nutrition Expert)*

*“I remember that there was resistance first on Gulazyk, but when people realized its benefit then it went very well on the ground, the usefulness of this powder was explained to young parents. The ADB project "Early Childhood Development at the Level of Local Communities" worked well. At one time Gulazyk was distributed with the help of related government agencies, because we had information about all low-income families. Still, a very large component was taken over by the health care system, because any mother who gave birth came to a doctor who, examining the child, could determine whether it was worth giving him/her Gulazyk” (Participant #6, Ministry of Labour and Social Development)*

*Challenges to Implementing Gulazyk*

Misconceptions and concerns regarding side effects associated with the MNP were identified by key informants, as they reported issues from moderate (e.g., diarrhea and nausea) to severe (e.g., challenges walking) and felt that these limited its uptake and continued use. The use of Village Health Committees (VHCs) to distribute Gulazyk was seen as ineffective during the pilot in Naryn oblast. Failure to deliver the MNP at convenient and preferred locations (e.g., pharmacies) as well as improper use of the Gulazyk were identified as barriers to behavior change in communities.

*“We have been working with Gulazyk, and spread it. It is added to the main/second course/meal, and was given to reduce anaemia among children. This work began in 2005 in Naryn. The program on creation of VHCs was launched by the Swiss project. First there was training of the population, how to give Gulazyk to children under 2 years (by haemoglobin indices). This work was carried out jointly with the Ministry of Health throughout the country. All children under 2 years were covered. After the adoption of Gulazyk, the children had diarrhea, nausea, but this was not connected with Gulazyk, the more it was distributed free of charge (there were complaints).” (Participant #20, Associate of Village Health Committees).*

*“We were told that before the consumption of the Gulazyk the child had problems walking, became covered with sweat a lot, his/her hair did not grow, and after the beginning of the Gulazyk consumption, his/her hair immediately grew, he/she began to sing songs, etc. Purchase of Gulazyk was sufficient, it was completely distributed. But then in one year the deputies did not go pass, did not approve the purchase of Gulazyk, they kept it for a very long time. It was necessary to understand, to inform on the usefulness of this powder. And then the deputies said that children can easily drink goat's milk and then they will be healthier, and all these were said by famous, respectable deputies. Then there was the supply of Gulazyk, but much effort was put. Then there was a desire to set up the production of Gulazyk here. The delivery of Gulazyk was partial, but not in all medical centres. Many mothers said that if it was sold in a pharmacy, they would buy it.” (Participant #9, Republican Center for Health Promotion)*

*“The trainings could not convince the population, I saw careless use of these vitamins, because before delivering this package, it is necessary to convey the importance of all this to the mother of the child. It was necessary to determine the specific stages of counseling; however, this point was missed. All health points were provided by Gulazyk, but counseling skills of doctors were not enough to convince parents.” (Participant #15, Mercy Corp, Mercy Corp)*

Overall inadequate or limited information to decision-makers and community members was associated with misconceptions, and a lack of trust and support of Gulazyk. Local deputies may have significantly challenged the implementation of Gulazyk as they delayed its approval and dissemination, shared inaccurate information and harmful messages about nutrition, side effects and the MNP. Local deputies used their political power and positions to influence the public’s perceptions and behaviours. Stability and sustainability of financing, and supply of the MNP were also seen to vary and may represent a factor that has challenged its implementation to date, and into the future.

*“Lack of information was the main barrier. One of the terrible factors happened when the deputies of the previous convocation made a statement that Gulazyk is scary, that they [international organizations] conduct experiments on our children, which will lead to infertility and so on. And these statements were all over the country, and of course people believed them. This greatly reduced the consumption of Gulazyk. The Association of Pediatricians, scientists wrote letters, explanatory work was carried out with these deputies. But when everyone understood the benefits, the matter was already done. This statement very strongly prevented this. All previous work could be deleted.” (Participant #18, Swiss Project, Swiss Project)*

*“When there were pilots in Talas, we tried not to attract attention to it, not to disclose it specifically through the media, so that there were no questions like “why in Talas?”, and from other regions, “why in Talas, and not with us?” Deputies were not aware of this event. When they [international organizations] decided to extend it to the whole country, the World Bank allocated $ 1 million, while ratifying this money in the Zhogorku Kenesh (Government), deputies first heard about this powder. They had many questions, vitamin supplements they took as biologically active additives, so they started to talk that much money is spent on buying biologically active additive, it's someone's business and so on. Moreover, the deputies until the end of not knowing the whole situation throughout the country began to say that it is poison or a useless thing. Kazakh media reported such information: "There is such hunger in Kyrgyzstan that children under the age of 5 are starving and to save the situation, the state began to give out powders to children in polyclinics. Will these powders save the lives of Kyrgyz children? ". After this article, there was a call from FMC in Tokmok and it became known that mothers, who had taken these powders before, returned Gulazyk back. They also said that if it is free, then this is a Chinese tablet, aimed at ensuring that children later have infertility. In China, such powders are given to children in order to further reduce the birth rate. When medical representatives explained that this powder is produced in India, there were also negative comments that it was dirty and something could be caught. It was also said that this powder does not fit the religion of Islam, because it is not produced in the Islamic Republic, for this it was necessary for the manufacturer to have a halal certificate and distribute it.” (Participant #19, Nutrition Expert)*

*“When DFID transferred $1 million to purchase a food package, and it was necessary to ratify this project in parliament, some deputies began to talk about the intentional genocide of Kyrgyz people, that the reception of Gulazyk will affect the gene pool of the nation. It turned out that some people were interested financially in providing these statements, possibly were waiting for monetary compensation for being silent. One man shook the papers from some laboratory, in which there was supposedly confirmation of the presence of harmful substances and so on. Plus, instability of financing: money came from different sources. Each of these sources has its own project cycles.” (Participant # 3, World Bank)*

*“This [Gulazyk coverage] is approximately enough to reduce the anemia, but then because of funding absence this powder was not purchased. Now the Gulazyk is ordered, but the order has not arrived yet, and it had already gone for three years since the last shipment was done.” (Participant #19, Nutrition Expert)*

*Vitamin A Supplementation*

Another project identified by key informants as contributing to the reduction of stunting among children was efforts relating to Vitamin A supplementation. This project lasted for six years, and its results are evaluated successfully by all experts who were involved in this program. The Kyrgyz Ministry of Health responded to the international guidelines endorsed by UNICEF and the WHO, and established a biannual universal distribution of high-dose vitamin A capsules for children 6–59 months. According to the WHO, 95% of children 6–59 months in Kyrgyz Republic receive vitamin A supplements twice each year (27).

Funding and implementation by UNICEF, use of mass media to sensitive and mobilize communities regarding the importance of vitamin A supplementation, and distribution at health facilities, helped to achieve widespread coverage and improvements in vitamin A deficiency. This program is currently not being implemented, as the Kyrgyz Government and Ministry of Health have failed to take ownership and continue implementation after substantial reductions and improvements were achieved. Lack of sustainability of this donor-funded initiative, and limited national prioritization may represent significant challenges for nutrition of children in the future.

*“UNICEF together with the Kazakh Academy of Nutrition determined in 2004 the prevalence of vitamin A deficiency was 32%. This is a very high figure, because when the value of vitamin A deficiency is higher than 25%, children need to receive vitamin A. Having obtained this result, the Ministry of Health created a working group that decided how this would be done. During five years, vitamin A was bought by UNICEF until 2011. Actual coverage was 90%, we went to monitoring and saw that almost all children received it. It was a very good program, which was conducted 2 times a year: children were invited and an oral vitamin A supplement was given. The population was informed by radio and television. In 2011, this program was suspended, because in 2010, 5% of the country's children had vitamin A deficiency, this is not an indicator for universal adherence, within the biologically accessible figures. It was possible to continue the program further, because as soon as you stop doing it, it can immediately return, but there was no money from the Ministry of Health, while from UNICEF, before the start of the program, was a mandatory condition that later the Ministry of Health would take ownership of the financing of the program at least 5-10%.” (Participant #19, Nutrition Expert)*

*“The women giving birth were given vitamin A in the maternity homes, after delivery. The children were given capsules on special days in polyclinics. It's a pity that this program was canceled, it was good. This program went very smoothly. There were delivery points in maternity hospitals, logistic ways of distribution were set up, all thanks to the fact that the Immunization Center [Republican Center for Immune Prophylaxis] was involved. Also, this vitamin was given to those who came for vaccination.” (Participant #10, SUN movement)*

*Salt iodization*

The addition of iodine to salt is a preventive nutrition intervention to address chronic undernutrition and frequently proposed introduced to address stunting in many low- and middle-income countries. The law on salt iodization was introduced in Kyrgyzstan in 2001, and mandates universal salt iodization. High-quality iodized salt is reportedly used in 76.1% of households in Kyrgyzstan(54). Innovative approaches to testing the quality of salt iodization (e.g., use of test sheets), creating a demand for iodized salt among the public and establishment of collaborations with local salt suppliers (e.g., village stores) represent key facilitators identified by key informants to policy initiative. VHCs advocated for stores to sell iodized salt, and sensitized store owners on the importance of salt iodization. Further, stores distributed test sheets to the public to allow for them to test the levels of iodine in the salt themselves.

*“We knew about the problem of salt iodine deficiency from UNICEF. [Name], head of European Commission in Kyrgyzstan at that time, came up with a brilliant idea how to use the test-lists that UNICEF had, and thanks to that you can find out if salt is iodized. These test-lists could expire in the near future, and for that they were not wasted. [Name] got a brilliant idea how to use them. The information was received from the VHC, by door-to-door method, when they visit each house and say how important it is to use iodized salt. Thus, he created a demand among the population for iodized salt. And the second thing he did was work with small suppliers of salt in the villages. In the villages, there were 1-2 stores at that time, and the VHCs visited them, they carried out work on the importance of selling iodized salt there. As well, he gave them these test-sheets, so that they could check the salt for the presence of iodine, when they will bring it. In such way the demand for iodized salt was created, and we said that if you import un-iodized salt, then the population will not buy it from you. So people could see themselves whether salt is iodized or not. And in the shops where they sell the products, they also handed out these test-sheets.” (Participant #3, World Bank)*

*Challenges to salt iodization efforts*

Although efforts relating to salt iodization were identified as longstanding initiatives, the process and standards for salt iodization in Kyrgyzstan was a significant concern among national key informants. Inadequate compliance to and enforcement of standards was identified as a significant challenge to ensuring standards and quality for salt iodization. Additional barriers to the implementation of salt iodization efforts included a shortage of required equipment, lack of capacity of salt producers, and the short lifespan of potassium iodate.

*“This law [on salt iodization] was adopted in 2001. The quality of salt iodization requires separate and close attention, it should be controlled. The Association of Village Health Committees practically monitors the quality of iodization almost every year and their studies have shown that the quality of salt iodization produced in Kyrgyzstan is low.” (Participant #10, SUN movement)*

*“Salt was iodized always, since Soviet times. Every person got used to consume only iodized salt. There is an association of salt producers, they buy potassium iodate jointly, and they have a certain revolving fund. Virtually all salt producers iodize it, because it is already built into the process. There are normative acts on salt iodization, but nobody follows it, there is no responsibility for violation of these acts, in case of less addition of potassium iodate to salt.” (Participant #11, Association of Suppliers)*

*“UNICEF purchased iodate for salt iodization for our manufacturing companies, but again, the problem is that small salt plants cannot do it right, and the quality of the salt is poor. We check salt iodization every year, and the quality suffers.” (Participant #9, Republican Center for Health Promotion)*

*“The main barrier is salt producers. They cannot stand the norm[s] - they do not buy potassium iodate. If they buy [it], then they do not have equipment that would evenly iodize salt. They put potassium iodate in plastic bottles, and here they sprinkle. So, in some place salt is iodated, in the other part is not. Here are such artisanal (handicraft/primitive) methods of iodination.” (Participant #18, Swiss Project)*

*“Iodization of salt is of great importance, i.e. greatly affects mental development. But it is known that the iodine in salt is broken down after a few months. Even if the package says that the salt is iodized, and it was stored in the warehouse for one year, then in a year this iodine will evaporate. [If] the iodine evaporates from this salt, especially in the open package, the content is reduced after a couple of months.” (Participant #14, Mandatory Medical Insurance Fund)*

Limited capacity and effort to monitor the impact of the consumption of iodized salt on children’s health and nutrition was observed.

*“We conducted a monitoring of salt iodization in 2009, and then it should be carried out every five years. And for monitoring, we need to go and the whole territory, at least by the cluster method, to see the children, their growth, the thyroid gland, to determine iodine in the urine, because there is one hormone that gives endocrine growth and gives growth hormone. Growth hormone is a difficult analysis, and to determine iodine deficiency, you can see the presence of iodine in the urine. And for this you need money, reagents; the Ministry of Health does not allocate money for this, because there is no money. For the last 8 years we have not monitored the level of iodine. Now we can determine whether the iodized salt was used only by salt itself, what is labeled on the bag, but the person can buy the iodized salt and store it for 9 months.” (Participant #17)*

Further, although coverage was supposedly national in scope, regional disparities in implementation were outlined. Key informants indicated that different prioritization and championing of salt iodization by local decision-makers and VHCs, as well as the politics of donors’ support led to inequitable implementation and coverage.

*“Coverage of the program was national, but there were variations in the context of regions, districts and even in the context of villages. And this depended on the commitment of those who were involved. In some villages VHC is active. Development partners are as active as they are. The GFD Association (Group of Family Doctors) agreed that the Asian Development Bank (ADB) would allocate money, but ADB could not give money to the Swiss project directly, it could only give to the GFD Association. Then there was an agreement concerning dates, the GFD Association had to assemble an audience that needed to be trained by health workers. Closer to this date, it became clear that the association had already spent this money [for] training on communications. Partners are different, and this also depends on the success of the program.” (Participant #3, World Bank)*

*Flour fortification*

Fortification of wheat and maize flour with micronutrients during the milling process, such as iron and folic acid, is considered a cost-effective approach globally to address and improve the nutritional status of children and to prevent birth defects (123). The Asian Development Bank (ADB), with support from Japan, initiated flour fortification in the Kyrgyz Republic. The Global Alliance for Improved Nutrition (GAIN) funded pilot projects and feasibility studies that demonstrated that the country had the capacity to fortify flour. Flour-fortification advocates have pursued the cause in Kyrgyzstan for over 10 years, despite numerous political setbacks. During that time, UNICEF provided technical support to local governments by implementing pilot projects in small flour mills, supporting the creation of the Association of Mills of Kyrgyzstan and supporting research on iron deficiency. To promote the new legislation, public hearings were held in Bishkek, with support from the local media. The *Law on Fortification of Baking Flour* was adopted in 2009 (89,124).

Key stakeholders emphasized that the adoption and implementation of a law on flour fortification and public awareness initiatives substantially contributed to the success of the initiative. In addition, the development of an alliance for the enrichment of flour brought together key stakeholders, including decision-makers and flour mills, to support the implementation of the law. An improved ‘premix’ formula for flour and donor contributions to fund domestic production of premix may improve both the quality and cost of fortified flour, and respondents indicated that these efforts represent potential facilitators of the policy going forward. Despite respondents outlining the importance of flour fortification and efforts to introduce the initiative, many respondents also shared challenges that have significantly limited effective implementation of the 2009 law.

*“There is a law on the enrichment of flour, where it is said that all imported and produced flour of the highest and first grade must be enriched. Recently, there have been changes in the administrative code that flour, which is not enrichable, should also be enriched. There are mills in the villages, and citizens of rural areas produce flour from the same wheat that they grow. Thereby they get flour of the second grade, so they do not enrich the flour. This program [on flour fortification] was initiated by UNICEF (purchase of the first batches of premix and measuring devices), due to the findings on the prevalence of anaemia among children and women in Kyrgyzstan. In other countries there is a successful experience in flour fortification, and this program was proposed to the government, then a law was drafted. The implementation of [the] flour fortification program was entrusted to the Ministry of Agriculture. Issues of control, flour monitoring, testing of flour fortification are [within] the powers of the Ministry of Health. On the instructions of the Government, the Ministry of Health and we [the Ministry of Agriculture] inspected the flouring mills several times. A list of enterprises producing flour of the first and highest grades was compiled, depending on the production capacity, a premix was distributed. All goals of the program were achieved, there was evidence that all flour was enriched with vitamins and is being enriched nowadays. Today, about 28% of flour is enriched, but you know, this figure is calculated for the whole produced flour (including flour of the second and third grades) in the country. And I think this is not correct, as this includes all flour that is not subject to enrichment.” (Participant #1, Ministry of Agriculture)*

*“An ‘Alliance for the Enrichment of Flour’ was created, which included decision-makers, flour mills and ministries to promote flour enrichment. We [UNICEF] bought feeders, trained the enterprises themselves to ensure quality control directly by the Department of Disease Prevention and State Sanitary and Epidemiological Surveillance, endless guidelines. In 2010, there were 51 mills. The law on the mandatory enrichment of flour was adopted in 2009, but it was not easy, because the interests of large flour mills were involved because they did not want to enrich. When we prescribed that this is mandatory for flour of the highest and first grades, they managed to enter one line "only for the flour of the state reserve." The main purpose of the law was destroyed. At the last moment it was introduced. The law did not go well, the control was very weak on the part of the Ministry of Health, because other laws supported small and medium enterprises (SMEs), the settlement of inspections, and the new law replaced the first law. There was work with the population to increase demand on fortified flour, but how to increase it, if such flour is absent on the market.” (Participant #7, UNICEF)*

*“This law was initiated by UNDP, our millers picked up this idea. They collected data, showed us what are the health problems. It seems to me that in our country, with all the problems that business has, we have a socially responsible business. Our producers went for it, initially they gave us premix for free, there were attempts to brand this flour with special signs, but unfortunately we were faced with the fact that we do not have the means to market and promote this branded flour. I would like more social videos or posters that would hang in medical institutions on necessity to consume fortified flour. Also, there is a problem - the majority of the population we have is poor. Producers produce fortified flour, but unprocessed flour is imported from Kazakhstan, which is cheaper. Accordingly, the population buys unenriched flour. We have a law on enrichment. More responsible millers, such as Akun Company and etc., enrich flour with vitamins, minerals and iron. This is very helpful, especially in regions where bread is the main part of the diet. Now the premix is ​​purchased independently by the producers, one producer was a pharmaceutical company will produce premix in the country, so it will be domestic. Before that we used the imported premix and did not have money to purchase it sometimes, there were difficulties with the purchase. For one manufacturer, it was necessary to have a small amount of premix, and it is necessary to purchase in general for all manufacturers in large batches, it turned out to be rather difficult, everyone had their own money, there were questions as to who will buy, how we will transfer, and the association, as a rule, does not have the right to trade, we are a non-profit organization. We have expressed all these problems with UNDP, FAO, therefore, funds have been raised so that the local producer produces a premix in the country, and each mill could directly contact them to purchase the premix.” (Participant # 11, Association of Suppliers)*

*“The earliest attempts to enrich the flour were in 1998, [in] the UNICEF pilot project for flour fortification. There were problems with premix, it influenced the quality of flour, and it did not become very tasty, the colour changed. Therefore, on the part of the population, there was dislike for this flour. In 2000, Kyrgyzstan participated in the Forum in Manila, where a resolution was adopted to combat nutrition problems, especially with micronutrient deficiency states [fortification of flour and salt]. After that, there was a major regional flour fortification project (Kyrgyzstan, Kazakhstan, Uzbekistan, Mongolia, and Azerbaijan) in 2001, supported by the Asian Development Bank and funded by [a] Japanese grant. In Kazakhstan, there is the National Academy of Nutrition, which developed a new premix formula, and was told that this premix should be used in all countries. Within the framework of this project, work began with mills, with large and medium flour mills, for which a premix and equipment was purchased. Further, the World Bank began financing the purchase and premix and technical improvement of these enterprises. The condition of the WB was that all logistics purchases should be through UNICEF. In 2009, all these partners began to lobby the law. In 2014 USAID attracted the American company GAIN, which assessed the implementation of this law. The main recommendation of the assessment was the creation of a revolving fund, it is necessary to collect some initial sum and purchase a premix, so that private traders buy the premix on their own. Now there is active support for the creation of this fund. Initially, the law was on enriching flour from state reserves, it did not say anything about enriching imported flour. In Russia, flour is not enriched, there is no such law; Kazakhstan has such a law, but they do not enrich the flour that is exported from the country. Then the law was altered: all flour of highest and first grades should be enriched. But some points were not solved, for example, what to do with imported flour, which is not enriched, should it be sent back or should a fine be imposed. Now these mechanisms are more defined. Worldwide, it was proved that fortifying flour is the easiest way to prevent iron deficiency among population. There were problems with premix, many manufacturers were not ready to buy it.” (Participant #16, WFP)*

*Challenges limiting progress towards flour fortification*

Challenges that limited implementation and efforts relating to flour fortification were also outlined by key informants. Limited resources and opportunities to effectively market and ensure recognizable branding for local fortified flour represented a potential barrier. Local production and consumption of unenriched flour was reportedly higher in rural areas. In general, key informants felt that there was a higher demand on imported flour than on domestic flour, even if it was not fortified. The high costs of enriched flour, as opposed to imported unenriched flour, is likely to have contributed to this demand and represented a significant barrier to public consumption of fortified flour. Similarly to challenges faced to implement salt iodization programs, limited monitoring and accountability of flour fortification efforts by the State and non-State actors continue to represent a significant challenge to the effective implementation of the policy. The development of lower grade non-enriched flour by large flour mill corporations and in general, poor control of the policy due to the politics and political interests of corporations were reported to substantially undermine successful policy implementation.

*“In rural areas where the majority lives, they consume more flour of their own production. Only the last years there began to appear places where they can enrich their flour. Before that it could not be enriched. Poor people do not buy fortified flour, they consume mealed flour that they produce on their own, which contains mineral elements. Flour of the highest and first grades should be enriched only” (Participant #3, World Bank)*

*“There was a whole program involving the Ministry of Health, the Ministry of Social Development, a department was established to coordinate the activities of all state structures, including National Statistic Committee, where we provided questionnaires of the interviewed people by random sampling, every five years. Due to this, we monitored how the situation in the mountainous areas, where we worked was changing. Large enterprises almost immediately began to enrich the flour. There was a problem with fortification among small rural enterprises, taking into account that the population preferred to take it from them. There was no guarantee that it was processed.” (Participant #6, Ministry of Labor and Social Development)*

*“At the moment, not all millers enrich flour, and this should be controlled by the state, since there is a law on the enrichment of flour. However there is no controlling body, there are no sanctions for non-compliance with the law.” (Participant #11, Association of Suppliers)*

*“There are many barriers. Because of the EAEU and lack of control at the border, unenriched flour is imported mostly from Russia and Kazakhstan. We work with kindergartens, schools, so that only enriched flour is used. The Ministry of Education helps us in this. Legislatively, it is now accepted that non-enriched flour should not be imported by Kyrgyzstan. As you know, the adoption of the policy to its implementation takes time. The participation of customs authorities, local authorities, the Ministry of Health, the Ministry of Education would be necessary.” (Participant #2,* *Center for State Sanitary and Epidemiological Surveillance of Bishkek)*

*“Flour fortification was one of the problems: flour of higher grades, flour in institutions for children must be fortified, i.e. enriched with these elements. Micronutrient deficiency is associated with a depleted composition of products that a woman consumes during her pregnancy. Depleted in the sense that consumption of meat is limited, and consumption of pastries, on the contrary, increased. At the same time baked products do not contain iron, consumption of vegetables and fruits is also reduced. Despite the warm climate, the consumption of these products among the adult population is very low. During pregnancy and after a woman rarely consumes vegetables and fruits. The same happens with the child: he/she sits on the same "diet" as his/her mother, what she eats, and then her child also eats.” (Participant #14, Mandatory Medical Insurance Fund)*

*Integrated Management Childhood Illness program (IMCI)*

In many low- and middle-income country settings child deaths occur due to inadequate access to health services, poor quality health services, and a lack of capacity among health professionals. To address this public health challenge, UNICEF and the World Health Organization have developed the Integrated Management of Childhood Illness (IMCI) strategy. IMCI adopts an integrated and holistic approach to reducing mortality, morbidity and to improve health and well-being among children under-five years of age. Key IMCI strategies include the overall training of health workers for improved case management and skills of health professionals, health system strengthening, and improved family and community health practices (125,126). The program’s focus on improved capacity and training of health professionals, and prioritization of funding for child health represented a positive contribution of the initiative.

*“One time the IMCI Institute was created (now this centre does not exist). The meaning of this program is that if a child falls ill, then a complex algorithm of actions is prescribed. As part of this program there is care for development from 0 to 3 years old, nutrition issues are also included. There were a lot of activities to integrate this program into the Kyrgyz state medical institutes for training, retraining, and training doctors. In the financing of health care, IMCI goes as a separate chapter.” (Participant #16, WFP)*

Many key informants recognized the positive impact and contributions of IMCI, while simultaneously identifying limitations. Poor acceptance and adoption of IMCI by experienced health professionals (e.g., “boycotting”) of the IMCI may have limited its effectiveness. The simplicity of the program, conflicts between the IMCI and training/curricula of health professionals, as well as additional/duplicate documentation and paperwork required led to substantial resistance among health care workers. Further, the high-cost of a donor-led vertical program, and the narrow focus on provision of health services (rather than health promotion and prevention) were issues identified by key informants.

*“IMCI is a good program, and it has worked well in other countries. In the face of a shortage of medical personnel - it was a necessity. We had a problem, high infant mortality because of late hospitalization, outflow of medical workers from the regions. In the city, it was not promoted, because medical workers directly boycotted it. Even in the medical academy it was included, but they felt that it was too simple for them and was pulling at the level of paramedics. For the regions, this was another vertical program, which was planted from above. Our doctors, who are old enough, could not accept this program. Those, who are younger, gladly took this program, and followed all the instructions for it. As main results, some components were implemented, such as child development, i.e. medical workers were engaged not only in treatment, but also in matters of nutrition and child development. They advised mothers on how to develop a child, how to stimulate his/her mental development, how to combine it with nutrition. The infant mortality rate falls, slowly, but the contribution of this program, probably, is also there. Now this program is called not as IMCI, but as the “Stationary directory: first aid to children", i.e. it was renamed. Now it is more adapted for doctors.” (Participant #10, SUN movement)*

*“IMCI was launched in 2000 or something. And due to the fact that a lot of money was issued for this project, it was incorrectly represented. IMCI was almost a rejected word afterwards. It was introduced only at the hospital level one time, then it was integrated into the primary healthcare too. We now call it a pocket guide. This is the primary and secondary health care.” (Participant #7, UNICEF)*

*“This program is very effective, but it is health services, which cannot affect the entire population. And they are applied when a person is already sick. And as a preventive measure, it has no direct influence. The doctor runs into the child when the child is already sick. Preventive measures are that the child eats, in what conditions lives, is exposed to any factors and risks, and this depends on the economic state of the family.” (Participant #14, Mandatory Medical Insurance Fund)*

*“This program went against the curriculum, it was presented in a different way, even in the documents there were such places. Often medical workers had to fill out the documentation separately for the Medical Health Insurance Fund, and separately for the IMCI; there was no synchronization between the audit organizations.” (Participant #10, SUN movement)*

*“At the moment, about 10 years [of] trainings on this program were not conducted, and those who have learned, perhaps already forget it. The main barrier of this program is that the higher schools did not accept this program, since it is too simplistic, professors did not accept such simplifications. Although if this program was successfully implemented, it could lead to some improvements.” (Participant #19, Nutrition Expert)*

*“Now we have resumed it, both in the primary and secondary, the community component is also there. A lot of money was spent on endless handouts, posters that were implemented at the national level, and were not lowered below. And it was so monopolized, stood apart. The Ministry of Health identified two people who were responsible for implementation of the project. And they kept it like that, and did not give the right to implement the project except themselves. Only 5-6 people were trained and travelled all over Kyrgyzstan. Now the process is different.” (Participant #7, UNICEF)*

*Benefits for low-income families*

The Kyrgyz Republic has spent approximately 1-1.5% of GDP on social assistance benefits and services since the 2000s. The country inherited a safety net from the Soviets consisting of a large number of poorly targeted, categorical, and costly benefits known as “privileges”. Prior to 2010, 39 categories of the population (e.g., World War II veterans, Chernobyl catastrophe liquidators, and people with disabilities) were entitled to more than 40 types of in-kind subsidies, representing a legacy of the past. These benefits included free transportation, discounted utility bills, free medicines, provision of a horse and a cart for rural transportation, free dentures, and others. Entitlements to such benefits did not necessarily mean that the benefits were fully financed and used. It was a partially-funded mandate shared with the service providers, and many people were excluded from the system because they did not have access to the actual services (e.g., people with limited ability to travel could not use free transportation; or, no supplies of free medicines existed in the stores) (27). At the end of 2009, the Government reformed the social protection system to improve its effectiveness and efficiency. One key area involved monetizing the categorical in-kind benefits and reducing the number of entitled categories. Only 25 groups of privileged beneficiaries continue to be entitled to categorical benefits, which are now provided in cash as flat monthly payments ranging from 1,000 to 7,000 som per month. Despite these reforms, the majority of measures introduced have not prioritized social protection resources to the lowest-income households. The remaining categories of beneficiaries do not correlate strongly with the poverty profile. The population most at-risk of poverty is small children, while the beneficiaries are mostly elderly. The entire set of social assistance programs includes only two that have poverty alleviation objectives and are targeted to lower-income households. Two targeted cash transfer programs introduced include the Unified Monthly Benefit (UMB) and the Monthly Social Benefit (MSB), which were introduced after Kyrgyz independence in 1991. They have the potential to support nutrition intervention components, and to contribute to the reduction in stunting.

The average monthly benefit for low-income families was only 768.4^[[4]](#footnote-4)^ Kyrgyz soms in the second half of 2015. Before July 1, 2015, the amount of it was even less. For the child, the state could pay 500, and 200, and 5 Kyrgyz soms, depending on the minimum guaranteed income for each family member. Since July 1, 2015 (after the adoption of the law), the amount of benefits for all minors have been fixed. Low-income families are those families, whose average monthly income per person is less than the minimum guaranteed income. In 2015, such amount was 810 KGS (127). Previously, the eligibility criteria for benefits was the household possession of fewer than three durable goods (e.g., TV, a washing machine and a refrigerator). Currently, consideration of household wealth is determined by possession of a car or tractor, land that generates income or livestock.

No key informants indicated that benefits for low-income families was a contribution towards the reduction in stunting. All key informants were doubtful on the effectiveness of this program, due to the nominal amount provided and extensive documentation/paperwork required to demonstrate eligibility. This highlights that cash transfer and poverty reduction programs in the Kyrgyz may not be effectively targeting and reaching individuals most in need of financial support.

*“The amount of these benefits is so small, but the necessity to pick a lot of different documents is so tough, so it is doubtful that it could help poor families to provide worthy nutrition” (Participant # 10, SUN movement).*

*“I did not meet young families who were receiving those benefits. There are a lot of requirements, and first of all existence of residence permit and other certificates. The size of those benefits is so small, that many low-income families do not even apply for them” (Participant # 8, National Center for Maternity and Childhood Protection)*

*Role of Donors*

Analysis of key policies and programs highlights the critical role of donors and development partners in the implementation of diverse nutrition-specific and –sensitive efforts. Key international organizations (e.g., UNICEF, UNDP, ADB, etc.) were identified as playing substantial roles in both pilot projects and widespread initiatives. Key informants recognized that donor contributions represented frequently both a facilitator and a barrier to implementation of policies and programs.

*“[The] situation with chronic malnutrition is improved, because there were data from a study conducted by UNICEF. The World Bank and UNICEF collaborated a lot on programs to reduce child malnutrition and stunting.” (Participant #3, World Bank)*

*“The economic situation in 1997 was worse, but I think that situation has changed due to activities of the government and donors.” (Participant #15, Mercy Corp)*

*“When financing was abruptly ended, it was only at the hospital level. IMCI was not introduced everywhere and immediately; there was no national coverage. Then there was no strong donor coordination, UNICEF was not the country office at that time. (Participant #7, UNICEF)*

*“Now the local “Biovit” Company produces Gulazyk, but it does not have international certifications. It is planned to purchase Gulazyk to the amount of $450,000; when the amount of purchase is above $100,000, then it is required the approval from donors and the availability of international certificates.” (Participant #8, National Center for Maternity and Childhood Protection)*

**Underlying causes**

Key underlying causes identified that improvements to the household environment, and improved feeding practices and food security represented contributions to reductions in stunting among children. Discussion regarding the role of access to health services was limited among key informants at the national level.

***Inadequate care and health services***

The problem of inadequate care and health services was discussed only briefly, and indirectly by key informants. This highlights that access to health services may not have been a substantial facilitator or contribution to improved nutrition and reduction of stunting.

*“[The] Batken [region] may be interesting because there is a high rate of violations of sanitary and hygienic standards. Very important is the prevention of intestinal infections, it is important not only what is consumed, but it is important that, whether it is lost. For example, parasitic diseases, helminths also contribute to the deficiency of these elements. The child can eat normally, but if he has helminthiases, she/he will lose it. Periodically, studies were conducted in Batken oblast on the diagnosis of helminthiosis, where the prescription of antihelminthic drugs was conducted. Once this program could not effect on stunting reduction, but such activities are also necessary.” (Participant #14, Mandatory Medical Insurance Fund)*

***Inadequate feeding practices and food insecurity***

Several respondents reported observations of diet diversification, improvements in infant feeding practices and increased food security among the population since the country’s independence.

*“People became more well-read and try to use breastfeeding, and not as in the olden days (parents/grandparents gave to suckle their small children tail fat). The latest data showed that people use this practice. Previously, many people previously believed that breast milk is not enough for a child to be full.” (Participant #20, Association of Village Health Committees)*

*“During the transition to the market economy, there was a shortage of food, there were queues for food. The collective farms were disintegrated, the planned economy ceased to exist, people began to grow production on their own plots, but at the same time they planted the same things, for example, potatoes, what is easy to grow, and there was no diversity.” (Participant #12, USAID)*

*“The 90s is a period which was characterized by strong decline, there were practically cases when there was nothing to eat. Food was scarce, expensive. Now life has become much better, many products are available, people are more informed.” (Participant #19, Nutrition Expert)*

Changes in family and household culture and tradition on improved feeding practices were outlined by key informants, as women were afforded the opportunity to consume more nutritious food.

*“In our country, especially in the Southern regions, and everywhere, in Kyrgyz families, how does it happen? Kelinka [young wife] is the last person to eat and she eats the leftovers of the meal. The best pieces of meat are given to her husband and her father-in-law. After our program, mothers-in-law themselves began to understand the importance of nutrition. They were also involved in discussions, explained to them that if they want to get healthy grandchildren, they should take care of their kelins [daughter-in-law]. And they began to change dietary habits, behaviour in their families. Women, who have young children, understood the necessity of a full sleep, appropriate food, relaxation. And they reacted with understanding to this. It was everywhere, not only in the Talas oblast.” (Participant #9, Republican Center for Health Promotion)*

Improvements were highlighted in knowledge, attitude and behaviours due to evidence-based research and awareness campaigns. These efforts have led to improved feeding practices, and increased rates of exclusive breastfeeding among women.

*“There was evidence that a woman should breastfeed for up to 6 months without giving anything [else]. However, the conducted survey has shown that women can feed their infants with everything, e.g. tea, water. And they gave it because of lack of knowledge. They first turn to their mothers-in-law, who recommend that this should be done. This is to say that the child is crying because you are hungry. In general, the Soviet school taught that it is necessary to feed every 3 hours. Therefore, people of my age still think that this is right. And they said it to their daughters and daughters-in-law. Because of wrong habits, and we learned from research, we decided to promote exclusive breastfeeding. Now, knowledge and behavior are quite high among mothers, they brought it to them. Still in the south, we campaigned every year starting in 2013, and only now we have achieved that the level of that mothers give tea to children has almost dropped by 0. It is very difficult to eliminate these habits.” (Participant #9, Republican Center for Health Promotion)*

*“In the past, beestings/foremilk/first milk/colostrum, i.e. the very first milk, was considered to be dirty and so it was filtered and not given to children. All these myths had to be broken. And it takes so long to attach during the first hours of birth, feeding - at the request of the child, to give nothing, except breast milk. We still practice such that after birth give the baby to try sary-mai (melted butter) or tail fat and they do not understand that such foods do ill/cause harm baby’s stomach, because the child cannot digest it. When we looked at the results of the CFH, at the level of the maternity homes everything is going well, up to 90% before applying to the breast, but as soon as the woman leaves home, adherence to breastfeeding falls at times. Because woman’s grandmother is near, who says "give water", "I gave water/tea and nothing, you were grown up." Exclusive breastfeeding was transferred to the primary medical assistance. Since 2012, we have started to work actively on informing the population. The army of health committees have materials; a lot of money was invested in them, in their trainings. Many clinical protocols have been prescribed that there should be a variety of nutrition plus protein, but we still have this fight. Therefore, we still have 18% stunting, because tradition to feed the child with bread, potatoes remains. Although there is work and we see some progress, but it could be better.” (Participant #7, UNICEF)*

*“Barriers to breastfeeding are mixtures for children. To sell them, producers promote their products through health workers, in maternity hospitals, as gifts to health workers, although there is a law. Now the law on breastfeeding is being revised and the responsibility for violating this law will be toughened.” (Participant #19, Nutrition Expert)*

*“When the Republican Center for Health Promotion, conducting a study through VHCs started talking to the population, it turned out that most of the mothers are breastfeeding, but in addition, they give their infants water, fat, which can lead to intestinal diseases, etc.” (Participant #16, WFP).*

*“As the years passed, people began to farm and grow necessary products for their own consumption. They try to sow potatoes, carrots, beets, if they do not have enough money, then they consume products from their kitchen garden.” (Participant #20, Association of Village Health Committees)*

**Immediate causes**

Improvements in dietary intake were observed including diversification of diets, knowledge of nutrition and preparation of fruits/vegetables. Despite progress, continuing challenges identified include increased consumption of carbohydrates (e.g., “junk food”), and regional inequities in dietary intake, and limited prioritization of spending to ensure adequate nutrition.

***Inadequate dietary intake***

Experts’ opinions on whether the population’s nutritional status improved varied, as 35% of experts indicated that improvements were achieved in the dietary habits of the Kyrgyz population and that this may have contributed to reductions in stunting. A lack of dietary diversity, and efforts to address iron-deficiency through micronutrient power (e.g., Gulazyk) were seen as potential contributions towards improved dietary intake. However, the majority of experts indicated that further improvements are required.

*“I would not say that we had malnutrition, but most of us eat monotonous food. There is no malnutrition, people consume food before they are saturated, but there is a shortage of vitamins. They eat a lot of flour products. In general, people who are below the poverty line are malnourished.” (Participant #20, Association of Village Health)*

*“There was also a study before and after the intervention, and it turned out that there was an approximate 25% decrease in anemia during a year. Gulazyk itself does not influence growth, it affects anemia, but it was necessary to start from something (any project/activity) to [have an] effect on health. Perhaps anemia is the cause of the stunted children in Talas.” (Participant #19, Nutrition Expert)*

*“I would not say that [the] situation with malnutrition has improved or deteriorated, but there are certain shifts. Somewhere those shifts are for the better, somewhere they are for the worse. If this issue is considered on the part of a balanced diet, then it certainly worsened.” (Participant # 5,* *Sanitary and Epidemiological Surveillance)*

Increased knowledge and understanding of nutrition and a balanced diet, and how to prepare and cook fresh fruits and vegetables, through access to materials (e.g., internet or cook book) were outlined as contributions towards improved dietary intake.

*“Previously, there was a large intake of carbohydrate food, now there is an understanding of the importance of a balanced diet, a variety of nutrition. Also, a large number of people know that it is recommended not to drink tea after meal, that drinking black tea immediately after eating interferes with the digestibility of iron and leads to iron deficiency anemia.” (Participant #2, Center for State Sanitary and Epidemiological Surveillance of Bishkek)*

*“Village citizens can cut cabbage, grate carrots, but this is not done, because residents of the Northern oblasts consider that special skills are necessary for salad cooking (for example, they could simply give the children tapped cabbage, beets, carrots). At the same time, for residents of southern parts of the country, it is normal. Recently, a book with salad recipes was distributed during a seminar in Naryn. One woman was so surprised that one can make an ordinary beetroot salad with the addition of salad oil, since she had a lot of it, and she did not know what to do with it. These books were distributed not only in Naryn, but also in Jalal-Abad and in the districts of Ala Buka, Chatkal and Toktogul.” (Participant #20, Association of Village Health Committees)*

*“Now, thanks to access to the Internet, a certain percentage of people [are] more responsible for their health and nutrition issues. At the same time, the other part has no idea at all. More educated people started more responsible with dietary habits, fertility issues, while the [rest of the] population [have] not.” (Participant #7, UNICEF, UNICEF)*

Despite an observed increase in income and overall wellbeing, one national stakeholder highlighted that individual/household investments in adequate nutrition and food still do not represent a priority for spending among the public. Inadequate measurement and research of dietary behaviours and behavior change, as well as inequity in dietary consumption are observed. Further, key informants indicated that poorly balanced diets, the increased consumption of junk food and deeply-rooted health behaviours represent key continuing challenges relating to malnutrition.

*“The population began to consume junk food more. In general, people's welfare has improved, but I think this has little effect on dietary habits, it is more reflected in the purchase of durable goods for the house (washing machines, etc.). Dietary habits have changed little; studies show that preferences are given to flour products, while consumption of vegetables, meat, eggs, milk is less. I think this is not due to the fact that they cannot afford it, they just used to eat like that.” (Participant #15, Mercy Corp)*

*“We began to study even deeper why people experience these problems. In fact, it turned out to be this. Take, say, Naryn. Many people think that people consume a lot of meat in Naryn. It turned out that this is not so. To the question, ‘How many times a week do you cook food from meat?’ And the maximum there was an answer, ‘once a week’. Some answered even ‘once a month’.” (Participant #18, Swiss Project)*

*“Information campaigns may affect the lifestyle, carried out by the structures of the Ministry of Health, namely, the Republican Center for Health Promotion, which is engaged in informing the public. Changes in lifestyle and dietary habits are very difficult to track, research requires a lot of money, at the moment there are no such studies.” (Participant #4*, *Ministry of Health)*

“*If you look at other statistics on the consumption of kcal, it improves every year, but if you look at the very poorest group, they consume 87% of the norm. And we have in this same statistics in Kyrgyzstan, there is insufficient intake of protein and fat, and a significant intake of carbohydrates. The World Bank studies say that children with chronic malnutrition in adulthood will earn 20% less than their peers who did not have chronic malnutrition.” (Participant #16, WFP)*

***Maternal characteristics***

Maternal characteristics were identified as potential challenges including iron deficiency among women and particularly those who were pregnant. The main reason of it is small consumption of fresh fruits and vegetables, and high consumption of bread products.

“*Yes, [the] situation with malnutrition has improved. But the level of iron deficiency among women is high as it was before. Even well-known chefs of Kyrgyzstan cook in that way [the processes of cooking, products are not healthy; use a lot of oil, fry everything, use a lot of fat and etc.] it is difficult to name ‘proper nutrition’.” (Participant #10, SUN movement)*

*“I know the issue with malnutrition in Kyrgyzstan, because our previous project, Community Action on Health Issues (SAN) (CAHI), began in [the] 2000s. And it began with the fact that we determined the health priorities of the population in the villages. And one of the priorities of the population was inadequate nutrition, including children, and a frequent disease is anemia, especially among pregnant women and children. This indicated that the problems of the health system, the problems of the state coincided with the problems of the common people. And among them were lack of nutrition and iron deficiency anemia among children and women” (Participant #18, Swiss Project)*

“*During pregnancy and after a woman rarely consumes vegetables and fruits. The same happens with the child: he/she sits on the same "diet" as his/her mother, what she eats, and then her child also eats.” (Participant #14, Mandatory Medical Insurance Fund)*

**Regional Stakeholders**

## Supplementary Table 10 Summary of in-depth interviews with community stakeholders

| Participant | Position & affiliation |
| --- | --- |
| **Interviews with community stakeholders** | |
| Batken oblast | Social worker |
| Batken oblast | Head of kindergarten |
| Batken oblast | Local Assembly Deputy, VHC member |
| Batken oblast | Cook, kindergarten |
| Batken oblast | Clinical manager, FDG, pediatrician |
| Batken oblast | Teacher at school, Rural Fund Manager, village activist |
| Batken oblast | Obstetrician |
| Batken oblast | VHC Chairperson |
| Talas oblast | Nurse, kindergarten |
| Talas oblast | Women Council member, activist, mathematics teacher |
| Talas oblast | Nurse, kindergarten |
| Talas oblast | Nurse, FDG |
| Talas oblast | Nurse of general clinics, FDG |
| Talas oblast | Cook, kindergarten |
| Talas oblast | Executive Secretary, Local Administration Office |
| Talas oblast | Mathematics teacher |

***Socioeconomic Indicators***

Overall, community stakeholders observed significant improvements in socioeconomic indicators over time, and felt that these represented key influences on the reduction of stunting nationally. Improvements were associated with increased migration and remittances, as well as international support and development.

*“Life has much improved. At those times, it was not a shame to ask neighbors for food, flour, butter, sugar. Now they do not do this, it's embarrassing. You can already conclude from this that people have become rich.” (Medical nurse, Talas oblast)*

*“In the 2000s it was so hard, we all ate flour from coarse barley. They [employers] paid salaries with galoshes. Galoshes were worn by all of us and children. It was so hard for us. Now the state has grown rich, management has improved, salaries have started to be issued, but life has basically improved because of migration.” (Activist, Batken oblast)*

*“Life did not improve much. A little. Lives of those who are associated with migration, have improved.” (Village Health Committee Chairman, Batken oblast)*

*“[It has] changed for the better. Rural health posts were opened, water was delivered, kindergartens were opened. All this is done with the help of international programs.” (Head of kindergarten, Batken oblast)*

*“Life has improved. In the 90s it was hard. Now it's good.” (Groups of family doctors, Talas oblast)*

Some experts indicated that improvements in quality of life were due to remittances.

*"Our families live not bad. There was one family in the village - there were absolutely nothing to eat, they were very poor. Half a year ago, the husband left for In migration. One of these days we [went] to check up how are their children. We found that now they eat jam, butter, honey etc. We so rejoiced for them. Before that, at best, only sugar and bread was on the table." (Social worker, Batken oblast)*

Although there were expressed mostly positive changes in socioeconomic indicators and nutrition, one stakeholder expressed her doubts regarding the quality of products nowadays:

*“In childhood, we ate home-made food: sour cream, melted butter, noodles made at home, ate homemade bread. There were grandmothers, there were their pensions. I think nutrition was good. Now everything is there, but everything is purchased. In our childhood, all the food was of high quality, but now children suffer from allergy. All food is stuffed with chemicals. We use chemicals ourselves so that there are no insects in the plants.” (Medical nurse, Talas oblast)*

**Basic causes: Nutrition specific and sensitive programs/policies**

There was no mention by community stakeholders of any state program generally, or any specific policies or programs to support child development and child nutrition. A mineral-vitamin supplement "Gulazyk" was discussed by three respondents, and IMCI was identified once.

*“Pupils in elementary schools receive free meal during classes. We give Gulazyk. Earlier for pregnant women a medicine for anemia for free was provided. Now we work on the IMCI program. Here the food is also considered. For example, if a child is sick, IMCI explains how to feed him/her. This program is more than 10 years old.” (Pediatrician, Batken Oblast)*

All community stakeholders underlined that they or other medical workers inform mothers on breastfeeding, explain how to breastfeed, promote exclusive breastfeeding during the first six months after child’s birth.

*“We learn how properly to hold the child, we say that let them suck the whole nipple, that mothers must feed their children being full of tenderness, with the cracking of the nipples, so that they do not stop breastfeeding. Some mothers, when they bake bread at a hot plate, think that the milk is puffed up by the heat and press this milk out of their body. We say that they should not do this because the woman's body regulates the temperature of the milk itself. We tell them that during the feeding first water comes out, then milk and only after this cream, so you need to feed the child to the end, not hurrying. We advise that until 6 months they are only breastfed so that they should not even give water, because children get everything they need with the mother's milk.” (Group of family doctors representative, Batken oblast)*

Regarding frequency of appealing to medical workers on breastfeeding and nutrition, experts noted facts of asking how to give Gulazyk, complaints on crackled nipples, asking for doctor’s recommendations on when to start giving food complementary, which products etc.

*“Women do what doctors say, but do not perform it 100%. Basically, all mothers-in-law decide. There is no special consultation for young women, because doctors do not have time to consult them. They make a quick inspection; they have no time to advise. When I was in Bishkek, they [medical workers] consulted very well there, they taught until the child was born, they were already taught how to nurse and educate them. And here is a lot of queue always; doctors barely have time to simply inspect. Young women are also not interested, do not ask.” (Social worker, Batken oblast)*

*“Now young mothers listen to doctors. And we did not obey; we did everything as our mothers-in-law would say. If they said: “Do not listen to the doctor, you never know what s/he can say, just cover the child in sheep fat", and we did so.” (Medical nurse, Talas oblast)*

*Role of donors*

Specialists mentioned assistance of international organizations (World Food Program, Agency for Technical Cooperation and Development (ACTED), Community Development and Investment Agency (ARIS) in programs realization.

*“WFP UN purchased equipment for schools and conducted trainings. One international organization conducted a project on plant growing: they taught how to choose seeds, how to grow plants, how to get a crop without using saltpeter, how to protect plants from insects. Within this project, 30 people were trained. 40 people were trained in cooking, 80 people were trained in processing products. For sewing 40 people were trained. For these students, there were more than 200 of them, as stipend were given 100 kg of flour, 10 liters of oil, studied 2-3 months, I also studied. ACTED has been making bi-annual projects to improve the standard of living of the population. It made 14 small business projects: they opened greenhouses, bought equipment for processing wool, sewing shops, and service stations. ARIS conducted clean drinking water. ARIS also made artificial insemination in agriculture, veterinarians bought motorcycles. The Public Fund of the DIA conducted training on the prevention of violence.” (Social worker, Batken oblast)*

*“Three years have passed since our kindergarten opened. We work with MercyCorp. They bought us food.” (Medical nurse, Talas oblast)*

**Underlying causes**

Community stakeholders in the Batken oblast identified regional inequities in access to safe drinking water. Further, they indicated that hygiene and sanitation does not represent a priority in many families, and that migration has influenced sanitation practices through challenges previous behaviours and norms.

***Unhealthy household environment***

In Batken, the establishment of a kindergarten and improved access to quality of water were reported by stakeholders as potential facilitators. Some respondents felt that migration may lead to exposure of other sanitation practices (e.g., in Russia) and that adoption of these norms represented potential contribution to improved sanitation and hygiene at the community level.

*“Children, who go to kindergartens, have good hygiene, and children who are not, who are at home all time, do not have good hygiene.” (Pediatrician, Batken oblast)*

*“In 2016, the presence of parasites exceeded the norm. All due to the fact that people drank water from the irrigation ditches and did not observe hygiene. And now [the] situation is better, the quality of water is better. Earlier it was normal to drink water from any sources. And it is very good that they [government] opened a kindergarten. Children in kindergarten are intelligent, comprehensively develop psychologically and their knowledge are at a good level. And they have access to good food.” (Pediatrician, Batken oblast)*

*“Thanks to migration, baths are built, aristons*^[[5]](#footnote-5)^ *are installed. Sanitary facilities are being installed, so people create a comfort conditions for themselves. Sanitary facilities are very important for hygiene.” (Activist, Batken oblast)*

Regional inequities in access to clean or improved water sources may represent a potential challenge limiting progress, as several community stakeholders in Batken oblast highlighted issues with unsafe water. Talas oblast

*“Hygiene is not observed here. Children play in the dust all the time, then come and sit down to eat without washing hands. Mothers have no time, and children do not create conditions. No washbasins. Mothers shout, "Go and wash your hands," and how s/he washes her/his hands is unknown, because their mothers are busy all the time. Only they [parents] began to create conditions after they looked how it is in Russia.” (Social worker, Batken oblast)*

*“There are all conditions for hygiene practice in the village.” (Nurse, Talas oblast)*

*“Hygienic practices are not paid enough attention by parents, just not everyone has access to clean drinking water and water for domestic needs is often inadequate.” (Medical worker, Talas oblast)*

Key challenges to sanitation and hygiene identified by community stakeholders included lack of money, shortage of safe drinking water, “laziness” of the public, failure to wash fruits and vegetables, and inadequate knowledge among public regarding safe drinking water sources. Despite recognizing potential positive influences of migration, community stakeholders also reported that the household environment of families in migration may also suffer due to the stress, impacting the mental health and development of children.

*“Basically, it is shortage of water. Lack of money is a problem: a powder, soap, chlorine - all this needs money.” (VHC, Batken oblast)*

*“Non-compliance with hygiene comes from laziness and lack of money.” (Social worker, Batken oblast)*

*“There are problems with the cleanliness of the village. They [citizens] do the cleaning in their yards, but they throw out the garbage anywhere, if only people do not see, and then children play in the garbage, get sick. Children drink water from the irrigation canals, there is no technology for the garbage cleaning. There is a garbage problem.” (Activist, Batken oblast)*

*“Those children, who do not go to kindergarten, play in the streets. They do not wash vegetables and fruits before they eat, drink water from the irrigation canals, then they have worms, parasites. There are a lot of us epidemic disease here.” (FGD, Batken oblast)*

*“Husbands are in migration. It is difficult for women to raise children. There is a big difference in mental and psychological development of children with parents in migration and those children who have a parent nearby.” (Head of kindergarten, Batken oblast)*

***Inadequate feeding practices and food insecurity***

Key challenges faced by pregnant women and young mothers included lack of money (to purchase food and to access health services in neighbouring villages, and a lack of high quality food. Key nutrition-related health challenges identified included anaemia, high levels of sugar, and blood pressure.

*“I think nutrition quality is good. There are also families who are poor, who cannot provide good nutrition for children. Now everything is good. Everyone has milk, but not everyone can afford to buy meat, low-income families are mostly young families.” (Village Health Committee Chairman, Batken oblast)*

**Immediate Causes**

***Inadequate dietary intake***

Key informants described current dietary intake and food among children in kindergartens (Supplementary Table 11).

## Supplementary Table 11 Description of food children consume in kindergartens in oblasts

| **Breakfast** | **Lunch** | **Dinner** |
| --- | --- | --- |
| Milk soup, bread, tea | Rice soup with meat, bread, tea | Buckwheat, bread, tea, sweets |
| Porridge, bread, tea, cheese | Lamian with minced meat, bread, tea | Mushy peas, bread, tea, apple |
| Semolina porridge, bread, butter, tea | Barley soup with meat, bread, tea | Stewed cabbage, bread, tea, waffles |
| Porridge, bread, tea | Soup with noodles and meat, bread, tea | Haricot, bread, tea |
| Cacao, bread roll | Pea soup, bread, tea | Macaroni, bread, tea, cookies |

Community stakeholders provided guidance and advice to parents and families on feeding practices for children, and indicated that they shared the importance of eating fresh fruits and vegetables, and consuming nuts, raisins and meat and dairy products. Similar nutritional recommendations were also provided to pregnant women.

*“We say to mothers of small children: puree everything you can. We cannot advise a specific meal, because many families do not have money, so that they can give the products that we recommend. We advise giving more meat” (Group of family doctors representative, Batken oblast)*

*“I advise mothers to give a lot of food fortified with vitamins to their children. Now there is a plant growing project. We explain that it is very important for children to eat vegetables and fruits. I am doing an explanatory work, do parental meetings and talk about it there. Even if there is milk, do not bother to boil and give it to children. We read lectures, we say that milk has calcium” (Head of kindergarten, Batken oblast)*

*“We recommend consuming food with vitamins, cereals, oatmeal, meat, dairy and fishing products” (Nurse, Talas oblast)*

*“We say that they [parent] should give meat, food, where there are a lot of vitamins. I recommend adding nuts and raisins to the honey to give the beets to children. If a child has cough, then parents should pour honey in radish and give this juice. I recommend treating children not with drug medicines, but with folk medicine” (Nurse, FDG, Talas oblast)*

*“They should not drink tea after meal, because the tea removes all useful elements from the body. We recommend pregnant women to sleep 2 times a day. Mothers-in-law or husbands of those pregnant women must buy food that pregnant women want to eat. We say to her relatives that they eat food for raising immunity. As well, we inform on that they should not eat warm food, but should prepare each time a fresh meal, so they do not eat fried food, but ate boiled. We go around every house and if there is a pregnant woman in a family, we require from her husband or her parents to prepare 10 liters of gasoline, on 3000 KGS. This money and gasoline they are obliged to prepare. We also say that they should know 2-3 numbers of taxi drivers who will take the mother in the maternity hospital” (Group of family doctors representative, Batken oblast)*

*“We say that they [pregnant] should not drink tea after eating for 2 hours. After lunch they should sleep for 2 hours, eat more meat, vegetables” (VHC, Batken oblast)*

Several stakeholders reported concerns regarding their evaluation of the quality of current nutrition among children, and emphasized inadequate consumption of vitamins and micronutrients. One respondent compared the inadequacy of the local dietary diversity and prioritization of nutrition among the Kyrgyz population to that of individuals from Uzbekistan.

*“…I cannot say that nutrition quality is very good. Children have anaemia, do not see a plump child, all children are thin. Due to malnutrition, the whites of children eyes are bluish” (Medical nurse, Talas oblast)*

*“Uzbeks focus on good nutrition unlike we - Kyrgyz. For example, we just eat navat. And they [Uzbeks] heat it in a cauldron reddish. They say that this will prevent a cold/fever. They consume a variety of food, soybeans, beans, nokot (sort of pea), also they consume a lot of pumpkin” (Social worker, Batken oblast)*

*“There is no such thing as giving vitamins. We give Gulazyk, but there are many [that] refuse it, because 1-2 years ago on TV there was false information that Gulazyk consumption causes cancer. They [mothers] say that children do not want to eat. They buy branded calcium. I saw several times that they bought from such network consultants as Tiens*^[[6]](#footnote-6)^*” (Group of family doctors, Batken oblast)*

***Maternal characteristics***

Decreased fertility rates was identified as a factor that has increased the nutrition and care for children.

*“Now young people take care of their children very well, because they give birth less [low number of children]. Now people are more prosperous than before.” (Activist, Batken oblast)*

**Mothers in Communities**

Focus group discussions were conducted in five villages across Batken (Ak-Suu, Kok-Tash) and Talas (Ak-Dobo, Manas, Sasyk-Bulak and Kara-Oi) oblasts. Women that had given birth in 1992-1997 and in 2012-2017 were recruited to participate. The research team in Kyrgyzstan conducted five FGDs, with a total of 69 women. Two FGDs were conducted with mothers of children born in the 1990s, and 2 FGDs were with women who gave birth in the last five years. One FGD was composed of women from both categories.

*Description of Respondents*

Demographic characteristics of FGD participants are outlined (Supplementary Table 12). All respondents were female, with ages ranging from 21 to 52 years old, and the average was 36 years. In rural areas, families are frequently large. Out of respondents 54% had three or more children, 37% had two children, and 9% had only one child (mostly young mothers from 20 to 25 years). The population sampled were well-educated, as 84% of women had secondary education, and 16% had completed higher education. No respondents had completed only primary education or attended no formal education. In general, women’s roles are associated with caring for and feeding children, and medical care for children and employees in kindergartens are frequently women.

Participants’ employment included civil servants in various state structures (e.g., rural government, school, and preschool institutions) (19%), entrepreneurs (5%), and the majority were “housewives” or stay-at-home parents (76%) (Figure below). However, during the interviews perceptions of what classifies as “formal work” was discussed, and many women only considered themselves formally employed if they worked in the civil service. Many seasonal workers, also called themselves “housewives”. Further, a woman that provides small services or who sells retail may also call herself a "housewife", and not an "entrepreneur." Also, many of them are engaged in agriculture (livestock and plant growing), which is not considered by rural citizens as an economic activity. Therefore, the picture of employment, with a deeper assessment, may be more optimistic. Approximately 20% (1 in 5) of respondents reported that their spouse is employed in another country, and provides support through remittances.

## Supplementary Table 12 Demographic characteristics of FGD participants

| **Demographic Characteristic** | **Number of Women (n=69)** | **Percentage (%)** |
| --- | --- | --- |
| Location |  |  |
| Batken | 16 (1992-1997)  19 (2012-2017) | 50% |
| Talas | 16 (1992-1997)  18 (2012-2017) | 50% |
| Number of children |  |  |
| 3 or more children | 37 | 54 |
| 2 children | 26 | 37 |
| 1 child | 6 | 9 |
| Education level |  |  |
| Higher education | 58 | 84 |
| Secondary education | 11 | 16 |
| Primary education | 0 | 0 |
| No education | 0 | 0 |
| Type of employment |  |  |
| Civil servant (rural government, school, preschool institutions) | 13 | 19 |
| Entrepreneur | 4 | 5 |
| Stay-at-home parent (“housewife”) | 52 | 76 |
| Gender |  |  |
| Female | 69 | 100 |
| Male | 0 | 0 |

**Basic causes**

Women that gave birth to children during the 1990s, highlighted that poverty was widespread. Since this time, respondents indicated that the quality of life in Kyrgyzstan has improved, and that this has led to the improved availability and security of food.

*“I remember how in the 1990s once ice cream was brought to our village. I could not find the 5 cents to buy a baby this ice cream, despite [that] he was asking for it.” (Respondent, 45 years old, Talas oblast)*

*“Doctors especially did not talk about nutrition, there was no money for food. There were hard times after USSR collapse, the food that was necessary for pregnant [women] was absent.” (Respondent, 50 years old)*

*“During 1990s, we had [to] barter. One sheep was equal to one bag of flour. Flour was not even of the highest grade.” (Respondent, Talas oblast)*

Exploring trends relating to the improvement of nutrition among this group, the majority (n=20) women felt that the quality of nutrition has improved over the last two decades. However, three respondents felt that there was an increase in poor quality food, and three did not feel that there has been a significant improvement or change in quality of nutrition over time. Participants reported that key facilitators included remittances, increased engagement in agriculture, and citizens’ efforts.

*“If you work, then you are not hungry” (Respondent, 45 years old)*

Women that gave birth to children during 2012 to 2017 indicated that there have been improvements in socioeconomic conditions, quality of life and nutrition. These changes were also attributed to remittances, engagement in the agriculture sector, and citizens’ efforts. In addition, they emphasized that increased pensions, and the engagement of men in construction, fields and mines represented factors that have improved the socioeconomic situation.

*“Due to fruits and vegetables which we grow by themselves” (Respondent, 34 years old)*

*“There are no working places in village. Thus, many [people] go to Russia to work and then send remittances” (Respondent, 34 years old)*

*“We have money because of migration, which [money] give us huge assistance”. (Respondent, 54 years old)*

Ability to buy washing machines, a multicooker, electric ovens, and access to water (water in houses) has facilitated domestic life of women and they have more free time, which they can dedicate to their children, or cook more diverse and high-quality food. Many respondents felt that nutrition became better because of migration, agriculture development (penetration of new cultures) and more conscious parenthood. New cultures are penetrated within the framework of the activities of international organizations (women were named, as well as by entrepreneurs who are trying to produce new products for sale).

*“Since Perestroika [after independence] life is much easier. There are heating bowlers, automatic washing machines, multicookers exist. And there is no necessity to make a lot of house duties as it was before.” (Respondent, 55 years old)*

*“Price on beans has increased till 54 KGS per kg, so many people [in Talas] became richer.” (Respondent, 35 years old)*

**Basic causes: nutrition-specific and -sensitive programs/policies**

Women’s shared perspectives on key nutrition-specific and -sensitive programs they felt contributed to improvements in nutrition including the promotion of exclusive breastfeeding and micronutrient supplementation.

*Breastfeeding promotion*

All FGD respondents (in both groups) indicated that medical workers informed them about the necessity of exclusive breastfeeding during the first six months after their child’s birth, and all respondents from 2010s groups added that they were informed on the importance of breastfeeding until children are two years of age. Respondents from 1990s group indicated that medical workers recommended preparing breasts during the last days of pregnancy, notably they taught to wash breasts by salt solution (salt and water). Respondents from 2010s group underlined that they were given booklets on how to breastfeed infants in maternity hospitals.

*“Doctors say, if you give food, then the child will wean from the breast.” (Respondent, 25 years old)*

*“Doctors [told] me how to breastfeed the first time. And told to breastfeed until [my] daughter was six months, exclusively.” (Respondent, 50 years old)*

*“Due to [the] American system, my baby was put on my belly after birth and was given [my] breast to try the first milk.” (Respondent, 22 years old)*

*Vitamins, Micronutrient Powders and Food Additives for Children*

Respondents of both groups indicated that they gave/give some vitamins or food additives to their children. Mothers who gave birth to their children during the 2012-2017 period indicated that they give calcium, iodomarine (iodine-containing drug), drugs to strengthen immunity, and ascorbic acid. Two respondents indicated that they give Gulazyk to their children. All mothers, whose children were born in 1990s, indicated that Gulazyk helped to change the dietary behavior of children in their villages. Concerning flour fortification, respondents indicated that they primarily consumed flour they personally produced. Respondents in the 1990s group in both oblasts indicated that those years were tough, as they had product deficits, particularly flour deficit. Women with children born in 1990s, thought they were giving vitamins with food.

*“During our time, as it was in Soviet times, cookies [biscuits] were with calcium. These cookies I gave my children.” (Respondent, 52 years old, Talas oblast)*

All respondents were concerned about the use of chemical fertilizers while growing wheat, potato, beans and other products for their own consumption. If they did not use chemicals, harvested crops could be poor. Workers of kindergartens indicated that for kindergartens, such products as seed oil, flour, macaroni and cereals are enriched with vitamins and delivered by MercyCorp every year.

*“When my children were small, there were not chemicals in products, yogurts, spicy baked breads, potato chips, Coca-Cola. There were only sunflower seeds which children ate.” (Respondent, 52 years old)*

*“Now there are a lot of chemicals everywhere: in potatoes, apples.” (Respondent, 47 years old)*

*“Now there are all products, but because of chemicals quality is bad.” (Respondent, 49 years old)*

**Underlying causes**

***Unhealthy household environment***

The "kayin-ene"^[[7]](#footnote-7)^ institute as a whole is weakening, and it gives an opportunity for independent decision-making and greater autonomy in the upbringing of children to parents, especially when a young woman is employed. Traditionally, the main actors in the process of physical care and education of children were the grandmothers of children, for example, the mother in-law. They were often leading the decision-making process. Employees of medical institutions noted that if the mother-in-law is good and friendly, her daughter/s-in-law is/are well dressed and fed, even if they are poor. But if the mother-in-law is not friendly and not good enough, then a woman in such family usually has problems during pregnancy, including a high physical load and malnutrition. As a rule, exploitative treatment and neglectful relationship is not projected on the child, as the child is already considered to belong to that family and mother-in-law tries to provide him/her adequate nutrition and proper care.

No significant differences in hygiene were identified between the 1990s and the present. Employees of medical institutions mentioned that they hold talks about the importance of washing their hands. Nevertheless, the prevalence of “dirty hand disease” indicates that conversations are not enough to change practice. Many families do not pay proper attention to such practices as brushing their teeth or washing their hands:

*"Children start to wash their hands only after 5 years old. If my son is allowed to wash his hands by himself, then he will always wash his hands. A bucket of water will be wasted. I do not allow him to wash his hands often. Often I just rub it with a damp rag. Many people do that." (Respondent, 28 years old)*

Similarly, to community stakeholders, women felt that that migration and importing of sanitation practices and standards from other countries (e.g., Russia) were key facilitators of improvement in this area.

***Inadequate care and health services***

Respondents, on the one hand, emphasized good work of local government medical institutions in training, informing parents about children’s health (including nutrition), as well as developing skills in caring for children. Younger women in the FGDs indicated that they may not entirely trust the doctors, and quality of care provided.

*“VHC members show how to breastfeed properly, when to give food, how to make an injection.” (Respondent, 25 years old)*

*“After childbearing, nurse visited me at home. After 1 month, I visited doctors to receive necessary vaccinations by plan.” (Respondent, 47 years old)*

*“Doctor visited me at home after childbearing. And then I came to doctor every month or once in 45 days to receive vaccination or to weight a child.” (Respondent, 25 years old)*

*“Arab people [investors] constructed [the] feldsher-midwife station. Medical workers treat children very good. We are glad.” (Respondent 47 years, old)*

*“We are afraid to go to medical workers - when the child gets sick, doctors immediately start yelling, saying “your child is almost dead”. And queues to those doctors are so long. It takes a whole day to stand with the child in hands." (Respondent, 26 years old)*

***Inadequate feeding practices and food insecurity***

*Complementary Food Initiation*

Most respondents from both groups breastfed their children until 1 year on average with some deviations (minimal age of stopping breastfeeding was 4 months, and the maximum reported was 4 years old). As well, not all respondents follow(ed) recommendations of medical workers regarding the complementary food initiation. Thus, one mother from the 2010s group recognized that despite doctors informing them on the necessity to start giving complementary food to her child after six months, she started to give [cow] milk after only one month. Other mothers started complementary feeding from potato puree or cookies soaked in tea/water. 90% of respondents from both groups indicated that when complementary food was initiated, they give the same soups that adults eat, simply pureed.

Children do not have a separate table or dishes. They often eat almost the same meal that adults consume, with the exception of porridge. There are several types of porridge that are prepared exclusively for children, for example, *bulamyk, byortmyochyuk, atala*, which are varieties of instant porridge on milk/water with the addition of butter and flour. The cookie in a softened form is also popular. This is the basis for the "children's table" in the focus villages. In some families, young parents ask the child what to cook for dinner, but this applies to more school-age children. As a rule, the mother prepares what her husband or senior family members want (particularly her husband's relatives). There is a tradition to give the best food to guests and to serve this food on an "important day". Many respondents noted that this tradition quietly outlives itself and mothers try to feed their children with such "stocked" products. Parents from Batken oblast shared perspectives on nutrition, including, "*when a child is well-fed, then s/he is healthy*" or "*Tasty food is useful*". In Talas oblast they say that "*Healthy food is "easy food*" or means rejection of animal fats, the use of enough vegetables and fruits, avoiding overeating, eating only "*when the sun is standing*." If the grandmother organizes meals in the family, she usually relies on her own ideas about how the child should be fed and on her experience. Employees of kindergartens believe: "*If the state approves, then it means it is health*y".

**Immediate Causes**

***Dietary Intake***

According to the majority of respondents, diet became more diverse, new products appear, both in raw form and in cooked food. Earlier they used to roast food in the past, now they use electric oven for baking.

*"Most of the children were fed with flour products. If it became known that some family is celebrating something with a large number of guests, we visited their houses with dishes and asked them to pour the broth for our children. I remember once I was coming back from such holiday with a can full of soup, but then fell down and shed all that I was carrying. I was crying because of insult." (Respondent, 42 years old, Talas oblast)*

*"Earlier in the 90s, the mandarins on the holiday were something amazing, we even said “It was a rich toi [celebration]! There were mandarins on the table! "Now we can buy mandarins. Not often, of course, but 1 or 2 times a month [we] can buy. Children really love [them]. We love mandarins ourselves." (Respondent, 49 years old)*

Respondents of 1990s group felt that access to information had increased, in particular through the internet, and that mothers are able to use it as a resource for recipes, as well as information and recommendations regarding nutrition. Respondents described trends in nutrition and dietary intake over time (Supplementary Table 13).

## Supplementary Table 13 Comparison of menu for children born in 1990s and 2010s

| **1990s** | **2010s** |
| --- | --- |
| Breakfast | Breakfast |
| - Bread with jam (families with high income) or just bread - Tea with sugar or milk - Heated food left over from the previous dinner | - Milk with bread - Airan or tea - Sandwiches (bread, butter, jam, sour cream) |
| Lunch | Lunch |
| - Fried potato/fried macaroni/buckwheat - Bread - Tea - Boiled Eggs (Occasional) | - Fried Potato/Fried macaroni/Buckwheat, - Bread - Tea - Boiled eggs (Occasional) - Patty, samsy^[[8]](#footnote-8)^, pies - Sausage goods, semi-prepared foods (when there is money) |
| Afternoon snack | Afternoon snack |
| - Did not have afternoon snack, children ate by themselves - Usually eat dry bread with tea with bread, if they ask - In summer: jarma^[[9]](#footnote-9)^ - Children took bread and ate outdoors during games | - Compote of dried apricots or apples, juices - Winter canned salads with bread - Sweets |
| Dinner | Dinner |
| - Hot meal: usually soups: rice (mastava^[[10]](#footnote-10)^), noodle soup - Sometimes main dishes: manty^[[11]](#footnote-11)^, oromo^[[12]](#footnote-12)^, plov^[[13]](#footnote-13)^. - Noted that some meal is prepared without meat | - Soups (noodle, rice, pea), dough dishes or manty, plov, oromo, kyulchotai^[[14]](#footnote-14)^ - Often cooked vinaigrette^[[15]](#footnote-15)^ |

There was not a significant change in consumed products and meals over time. However, it is interesting to note that respondents with children in 1990s reported that some meat dishes would be prepared without meat/protein. Individuals with children born in 2010s, did not mention this limitation. All women (100%) in both categories indicated that there was/is no special time for feeding babies, except for breastfeeding. Children from aged 2 years eat with adults, usually three times a day. During the last 10 years, parents started to buy chairs for children under 5 years of age.

*“In the 90s, and even earlier, the children sat at our knees or on knees of their grandparents. Now there are many different chairs for children with a table. And families who have the opportunity to buy it, purchase such chairs for their children” (Respondent, 52 years old)*

***Maternal Characteristics***

Health problems identified by women in communities included anaemia, high blood sugar and high blood pressure. Further, decreased fertility was reported among older FGD respondents and this was linked to improved health behaviours, awareness of nutrition and improved diet among children.

*“Now the youth do not give birth to 5-6 children like us. They have 2-3 children each. They read everything; they try to cook something for their children" (Respondent, 52 years old)*

# **Appendix References**

1. World Bank. World Bank Indicators. 2019.

2. United Nationas Development Programme. Kyrgyzstan [Internet]. Human Development Reports. Available from: http://hdr.undp.org/en/countries/profiles/KGZ

3. Tretiakova T. The National Monitoring and Evaluation Network of the Kyrgyz Republic [Internet]. 2012. Available from: https://www.ioce.net/download/national/Kyrgyz_MENetwork_CaseStudy.pdf

4. Howell J. Poverty, Children and Transition in Kyrgyzstan: Some Reflections from the Field. J Int Aff. 1998;52:131–44.

5. Ministry of Agriculture and Land Improvement/Ministry of Health. Kyrgyz Republic Proposal for Funding for Agriculture Productivity and Nutrition Improvements under the Global Agriculture and Food Security Program (GAFSP). 2012;

6. Kyrgyz Republic/UN. United Nations Development Assistance Framework (UNDAF) for the Kyrgyz Republic 2012-2016. Bishkek, Kyrgyzstan; 2011.

7. Falkingham J, Evandrou M, Lyons-Amos L. Inequalities in child and maternal health outcomes in CEE and the CIS. ESRC Centre for Population Change. 2012.

8. Global Nutrition Report. Kyrgyzstan: Nutrition Country Profile. Washington, D.C.; 2014.

9. Kyrgyz Republic. Trade Policy Review. 2006.

10. World Bank. Update on poverty in the Kyrgyz Republic. 2011; Available from: http://go.worldbank.org/6FR5P3UJW0

11. International Monetary Fund. The Kyrgyz Republic: Poverty Reduction Strategy Paper. Washington, D.C.; 2014.

12. UHC 2030. Joint Assessment of the Kyrgyz Republic National Health Reform Program - Den Sooluk 2012 - 2016 [Internet]. Available from: https://www.uhc2030.org/fileadmin/uploads/ihp/Documents/Key_Issues/NHP___JANS/KGZ JANS.pdf

13. WHO/Kyrgyz Republic Health Policy Analysis Centre. Integration of the Sustainable Development Goals 2030 in the strategic programs of Kyrgyzstan ’ s healthcare sector and the country ’ s Development Strategy 2030. Bishkek, Kyrgyzstan; 2017.

14. Giuffrida A, Dale EM, Jakab M. Toward universal coverage in health : the case of the state guaranteed benefit package of the Kyrgyz Republic (Inglés). 2013;1–34. Available from: http://www-wds.worldbank.org/external/default/WDSContentServer/WDSP/IB/2013/02/01/000333037_20130201153449/Rendered/PDF/750060NWP0Box30l0Coverage0in0Health.pdf

15. WHO/DFID. Evaluating Manas Health Sector Reforms (1996-2005) [Internet]. Policy Brief #10. Bishkek; Available from: http://hpac.kg/wp-content/uploads/2016/02/Policybrief10_eng.pdf

16. Lerman Z, Sedik D. Agrarian Reform in Kyrgyzstan : Achievements and the Unfinished Agenda. 2009.

17. National Statistical Committee of the Kyrgyz Republic. Agriculture. 2018.

18. Kroeger A, Anderson KH. Remittances and the human capital of children: New evidence from Kyrgyzstan during revolution and financial crisis, 2005–2009. J Comp Econ. 2014;42:770–85.

19. Balabanova D, Roberts B, Richardson E, Haerpfer C, McKee M. Health care reform in the former Soviet Union: Beyond the transition. Health Serv Res. 2012;47:840–64.

20. Ibraimova A, Akkazieva B, Ibraimov A, Manzhieva E, Rechel B. Kyrgyzstan: Health system review. Health Syst Transit [Internet]. 2011;13:xiii, xv–xx, 1–152. Available from: http://libaccess.mcmaster.ca/login?url=http://ovidsp.ovid.com/ovidweb.cgi?T=JS&CSC=Y&NEWS=N&PAGE=fulltext&D=emed13&AN=362736413%0Ahttp://sfx.scholarsportal.info/mcmaster?sid=OVID:embase&id=pmid:21697030&id=doi:&issn=1817-6127&isbn=&volume=13&issue=3&spage

21. National Statistical Committee of the Kyrgyz Republic. INFORMATION BULLETIN OF THE KYRGYZ REPUBLIC ON FOOD SECURITY AND POVERTY [Russian]. 2016;

22. The World Bank. GDP (current US$) [Internet]. 2018. Available from: https://data.worldbank.org/indicator/NY.GNP.MKTP.CD?locations=KG

23. Jacobs C. Evaluating the Comprehensive Development Framework in Kyrgyz Republic, Central Asia Magic Bullet or White Elephant? Evaluation [Internet]. 2005 [cited 2018 Jul 5];11:480–95. Available from: www.kyrgyzinvest.kg

24. Kosec K. Land Reform and Child Nutrition: Evidence from Kyrgyzstan. In: IFRPI, editor. Annual Euraisan Food Security Conference. Dushanbe, Tajikistan; 2017.

25. World Bank. Current health expenditure (% of GDP). Open Data. 2018.

26. UNDP. The Kyrgyz Republic: Millennium Development Goals - progress report. Bishkek, Kyrgyzstan; 2003.

27. World Bank, UNICEF. Situational Analysis: Improving economic outcomes by expanding nutrition programming in the Kyrgyz Republic. Washington, D.C.; 2011.

28. USAID/SPRING. Baseline Nutrition Survey in the Kyrgyz Republic. 2015;

29. FAO. Kyrgyzstan [Internet]. FAO STAT. 2017. Available from: http://www.fao.org/faostat/en/#country/113

30. Piaro B. Assessment of the Impact of the Mercy Corps Kyrgyzstan Food for Education 2010 Program. Georgia State University; 2013.

31. World Food Programme. Emergency Food Security Assessment (EFSA). 2012.

32. Gausman J, Guevara IM, Subramanian S V, Razak F. Distributional change of women’s adult height in low- and middle-income countries over the past half century: An observational study using cross-sectional survey data. Plos Med. 2018;15:e1002568.

33. National Statistical Committee of the Kyrgyz Republic. Women and Men of the Kygyz Republic. Bishkek, Kyrgyzstan; 2013.

34. FAO. Regional Overview of Food Insecurity Europe and Central Asia [Internet]. 2015. Available from: http://www.fao.org/3/a-i4649e.pdf

35. Ministry of Justice. Law on protection of breastfeeding and regulation of marketing of infant food. Kyrgyzstan: Ministry of Justice; 2008.

36. United Nations Children’s Fund (UNICEF), World Health organization (WHO). Country experiences with the Baby-friendly hospital initiative, compendium of case studies from around the world [Internet]. 2017 [cited 2018 Jul 9]. Available from: https://www.healthynewbornnetwork.org/hnn-content/uploads/BFHI_Case_Studies_FINAL_.pdf

37. UNICEF. UNICEF annual report. 1996.

38. Huseynov S, Steinglass R. Immunization and health sector reform in the Kyrgyz Republic [Internet]. World Health Organization. Geneva, Switzerland; 1999. Available from: http://unpan1.un.org/intradoc/groups/public/documents/apcity/unpan046798.pdf

39. Ministry of Justice of the Kygyz Republic. Government decree state program “Healthy Nation 1994-2000.” Bishkek, Kyrgyz Republic;

40. World Bank. Participatory Poverty Assessment in the Kyrgyz Republic For the World Development Report 2000/01 [Internet]. Bishkek, Kyrgyz Republic; 1999. Available from: http://siteresources.worldbank.org/INTPOVERTY/Resources/335642-1124115102975/1555199-1124138866347/kyrgyz.pdf

41. Ministry of Health. Order of the Ministry of Health of the Kyrgyz Republic on the observation of healthy children at the levelof primary health care. Bishkek, Kyrgyzstan; 2010.

42. The World Bank. Kyrgyz Republic: Early Childhood Development. 2013.

43. UNICEF. Maternal and Newborn Health in Chui Province & Kyrgyzstan: Assessment and Implications for Interventions. Bishkek, Kyrgyz Republic; 2009.

44. WHO. Den Sooluk National Health Reform Program in the Kyrgyz Republic for 2012-2016. 2011;1–59.

45. UNICEF. Situation Assessment of Children in the Kyrgyz Republic. Bishkek, Kyrgyzstan; 2011.

46. Novovic T, Ibraeva G, Gabdulhakov R. Mid Term Review of the United Nations Development Assistance Framework (UNDAF) for Kyrgyz Republic (2012 - 2017). Bishkek, Kyrgyzstan; 2015.

47. World Health Organization. Den Sooluk National Health Reform Program in the Kyrgyz Republic for 2012 - 2016 [Internet]. Global database on the Implementation of Nutrition Action (GINA). 2012. Available from: https://extranet.who.int/nutrition/gina/en/node/23556

48. World Health organization (WHO). Evaluation in Kyrgyzstan [Internet]. World Health Organization; 2018 [cited 2018 Jul 9]. Available from: http://www.euro.who.int/en/health-topics/Life-stages/child-and-adolescent-health/child-and-adolescent-health2/children-at-home-and-in-primary-health-care/evaluation-in-kyrguzstan

49. Fact Fish. Kyrgyzstan: Vitamin A supplementation coverage rate, child, aged 6-50 months (%) [Internet]. 2018. Available from: http://www.factfish.com/statistic-country/kyrgyzstan/vitamin a supplementation

50. Wirth JP, Petry N, Tanumihardjo SA, Rogers LM, McLean E, Greig A, Garrett GS, Klemm RDW, Rohner F. Vitamin a supplementation programs and country-level evidence of vitamin A deficiency. Nutrients. 2017;9:1–18.

51. Black RE, Victora CG, Walker SP, Bhutta ZA, Christian P, De Onis M, Ezzati M, Grantham-Mcgregor S, Katz J, Martorell R, et al. Maternal and child undernutrition and overweight in low-income and middle-income countries. Lancet. 2013;382:427–51.

52. Black RE, Allen LH, Bhutta ZA, Caulfield LE, De Onis M, Ezzati M, Mathers C, Rivera J. Maternal and Child Undernutrition Maternal and child undernutrition: global and regional exposures and health consequences. Lancet. 2008;371:243–60.

53. Global Forum on Food Security and Nutrition in Europe and Central Asia. Food security and nutrition programme for Kyrgyzstan in action. How to implement policy in the most efficient way? 2016;1–6. Available from: www.fao.org/fsnforum/eca/activities/discussions/kyrgyzstan%0Awww.fao.org/fsnforum/eca%0Ahttp://stat.kg/media/publicationarchive/a088fb17-45ea-4cf2-9e70-e253d44ea998.pdf

54. UNICEF. Maternal and Newborn Health in Chui Province & Kyrgyzstan: Assessment and Implications for Interventions. Bishkek, Kyrgyz Republic; 2009.

55. Sida L, Leach A, Burzhubaev T. The Kyrgyz Republic: An evaluation of WFP’s Portfolio (2008 - 2012). Rome; 2013.

56. Kroeger A, Anderson K. Remittances and Children’s Capabilities: New Evidence from Kyrgyzstan, 2005-2008. IZA Discussion papers. Berlin, Germany; 2011.

57. Novovic T, Ibraeva G, Gabdulhakov R. Mid Term Review of the United Nations Development Assistance Framework (UNDAF) for Kyrgyz Republic (2012 - 2017). Bishkek, Kyrgyzstan; 2015.

58. UNICEF. Situation Assessment of Children in the Kyrgyz Republic. Bishkek, Kyrgyzstan; 2011.

59. Ministry of Health. Programme of the health sector of the Kyrgyz Republic on climate change adaptation 2011-2015. Bishkek, Kyrgyzstan; 2011.

60. International Food Policy Research Institute (IFPRI). Global Nutrition Report 2016: From Promise to Impact: Ending Malnutrition by 2030. Global Nutrition Report. Washington D.C.; 2016.

61. World Food Programme. National Food Security Atlas - Kyrgyz Republic 2015. Bishkek, Kyrgyz Republic; 2016.

62. Dhur A. Food Security Assessment in the Kyrgyzstan Republic. 2008.

63. UNICEF/Centre for Social and Economic Research. Effectiveness of Benefits to Families and Children in the Kyrgyz Republic. Bishkek, Kyrgyzstan; 2008.

64. National Statistical Committee of the Kyrgyz Republic, UNICEF. Multiple Indicator Cluster Survey 2006, Kyrgyz Republic [Internet]. Bishkek, KyrgyzstanMultiple INdicator Cluster Survey,; 2007. Available from: https://mics-surveys-prod.s3.amazonaws.com/MICS3/Europe and Central Asia/Kyrgyzstan/2005-2006/Final/Kyrgyzstan 2005-06 MICS_English.pdf

65. National Statistical Committee of the Kyrgyz Republic (NSC), MInistry of Health [Kyrgyz Republic], International I. Kyrgyz Republic Demographic and Health Survey 2012 [Internet]. Bishkek, Kyrgyz Republic, and Calverton, Maryland; 2013. Available from: https://dhsprogram.com/pubs/pdf/FR283/FR283.pdf

66. UNDP. The Kyrgyz Republic: Millenium Development Goals - progress report. Bishkek, Kyrgyzstan; 2003.

67. UNICEF. Kyrgyzstan: Country programme document 2012 - 2016. 2011.

68. Otunchieva A, Withanachchi S, Ploeger A. Role of mothers as the primary caretakers for preventing child malnutrition in Batken province in Kyrgyz Republic. Ann Nutr Metab. 2015;67:432.

69. Babu S, Reidhead W. Poverty, food security, and nutrition in Central Asia: a case study of the Kyrgyz Republic. Food Policy. 2000;25:647–60.

70. UNICEF/Centre for Social and Economic Research. Effectiveness of Benefits to Families and Children in the Kyrgyz Republic. Bishkek, Kyrgyzstan; 2008.

71. UNICEF. Follow-Up Survey of Nutritional Status in Children 6-29 Months. Bishkek, Kyrgyzstan; 2015.

72. UNICEF/CDC. Assessment of the nutritional status of children 6-24 months of age and their mothers, rural Talas oblast, Kyrgyzstan 2008. Bishkek, Kyrgyzstan; 2010.

73. SUN Movement. Kyrgyzstan (Country Profile). 2014.

74. Research Institute of Obstetrics and Pediatrics [Kyrgyz Republic], Macro International Inc. Kyrgyz Republic Demographic and Health Survey. Calverton, Maryland USA;

75. Lundeen E, Imanalieva C, Mamyrbaeva T, Timmer A. Integrating Micronutrient Powder into a Broader Child Health and Nutrition Program in Kyrgyzstan. In: De Pee S, Flores-Ayala R, Van Hees J, Jefferds ME, Irizarry L, Kraemer K, Monterosa E, Timmer A, editors. Home Fortification with Micronutrient Powders (MNP). Basel, Switzerland: Sight and Life, UNICEF, WFP, HF-TAG; 2013. p. 23–30.

76. Victora C G, Huttly, S R, Fuchs, S C, Olinto, M T. The role of conceptual frameworks in epidemiological analysis: a hierarchical approach. Int J Epidemiol. 1997;26:224–7.

77. Jann B. The Blinder–Oaxaca decomposition for linear regression models. Stata J. 2008;8:453–79.

78. Headey D, Hoddinott J, Park S. Accounting for nutritional changes in six success stories: A regression- decomposition approach. Glob Food Sec. 2017;13:12–20.

79. Headey DD, Hoddinott J. Understanding the Rapid Reduction of Undernutrition in Nepal. PLoS One. 2015;10:e0145738.

80. Headey D, Hoddinott J, Park S. Drivers of nutritional change in four South Asian countries: A dynamic observational analysis. Matern Child Nutr. 2016;12:210–8.

81. Woodruff BA, Wirth JP, Bailes A, Matji J, Timmer A, Rohner F. Determinants of stunting reduction in Ethiopia 2000 – 2011. Matern Child Nutr. 2017;13.

82. Alderman H, Headey D. The timing of growth faltering has important implications for observational analyses of the underlying determinants of nutrition outcomes. PLoS One. 2018;13:e0195904.

83. Restrepo-Méndez MC, Barros AJ, Black RE, Victora CG. Time trends in socio-economic inequalities in stunting prevalence: analyses of repeated national surveys. Public Health Nutr. 2014;18:2097–104.

84. Sandelowski M. Focus on Qualitative Methods Sample Size in Qualitative. Res Nurs Heal. 1995;18:179–83.

85. Green J, Browne J. Principles of Social Research. Green, J. & Browne J, editor. Maidenhead: Open University Press; 2009.

86. Ryan L, Golden A. “Tick the box please”: A reflexive approach to doing quantitative social research. Sociology. 2006;40:1191–200.

87. Tong A, Sainsbury P, Craig J. Consolidated criteria for reporting qualitative research (COREQ): A 32-item checklist for interviews and focus groups. Int J Qual Heal Care. 2007;19:349–57.

88. Global Forum on Food Security and Nutrition in Europe and Central Asia. Food security and nutrition programme for Kyrgyzstan in action. How to implement policy in the most efficient way? 2016.

89. Law on the Fortification of Baking Flour. Kyrgyzstan; 2009.

90. Shevchuk S, Ghauri K. Afghanistan/Central Asia Regional Food Fortification Program [Internet]. Geneva, Switzerland; 2015. Available from: https://www.gainhealth.org/wp-content/uploads/2014/07/Analysis-of-Food-Fortification-in-CAR-Afghanistan-and-Pakistan-ENG.pdf

91. UNICEF. Kyrgyzstan enacts law on flour fortification to fight “hidden hunger” [Internet]. Kyrgyzstan. 2009. Available from: https://www.unicef.org/nutrition/kyrgyzstan_49274.html

92. Sultanalieva RB, Mamutova S, Van Der Haar F. The current salt iodization strategy in Kyrgyzstan ensures sufficient iodine nutrition among school-age children but not pregnant women. Public Health Nutr [Internet]. 2009 [cited 2018 Jul 3];13:623–30. Available from: https://www.cambridge.org/core/services/aop-cambridge-core/content/view/AD0931FB4780B2431CCEDA02DC609858/S136898000999200Xa.pdf/current_salt_iodization_strategy_in_kyrgyzstan_ensures_sufficient_iodine_nutrition_among_schoolage_children_but_not_pregnant_wo

93. Law of the Kyrgyz Republic. On Prophylaxis of Diseases, Caused by Iodine Deficit. Ministry of Health; 2000.

94. Tazhibayev S, Dolmatova O, Ganiyeva G, Khairov K, Ospanova F, Oyunchimeg D, Suleimanova D, Scrimshaw N. Evaluation of the potential effectiveness of wheat flour and salt fortification programs in five Central Asian countries and Mongolia, 2002-2007. Food Nutr Bull. 2008;29:255–65.

95. The Government of the Kyrgyz Republic. on Approval of the Food Security and Nutrition Program in the Kyrgyz Republic for 2015-2017. 2015.

96. Boldjurova I. Tokyo High-level Forum on Health Millennium Development Goals (MDGs) in Asia and the Pacific [Internet]. Tokyo, Japan; 2005. Available from: http://www2.wpro.who.int/NR/rdonlyres/D468E4EE-3386-44E5-A93E-00CBA1E13F6F/0/10KyrgyzIodinedeficiencyandfoodfortificationIBoldnurova.pdf

97. UNICEF. Sustainable Elimination of Iodine Deficiency: Progress subce tge 1990 World Summit for Children [Internet]. New York, NY; 2008. Available from: https://www.unicef.org/iran/Sustainable_Elimination_of_Iodine_Deficiency_053008%281%29.pdf

98. Asia Development Bank. Legislation and Regulations on Food Products Fortifcation in Central Asian Countries [Internet]. JFPR 9052 Regional Project. Available from: http://kan-kaz.org/english/konference/Legislation_base.pdf

99. National Institute of Strategic Research, Saikal M. Pros and cons of national health reform programs “Manas”, “Manas Taalimi” and “Den sooluk.” Bishkek, Kyrgyz Republic;

100. Yamaguchi A, Danilenko A. Water and Sewerage Utilities in the Kyrgyz Republic: Performance INdicators.

101. World Health Organization. Comprehensive Development Framework of the Kyrgyz Republic [Internet]. 2003. Available from: https://extranet.who.int/nutrition/gina/sites/default/files/KGZ 2002 Comprehensive Development the Framework of Kyrgyz Republic to 2010.pdf

102. Kyrgyzstan Development Gateway. Monitoring and Evaluation [Internet]. Comprehensive Development Framework for the Kyrgyz Republic. 2001. Available from: http://eng.gateway.kg/content/strategies/cdf/92

103. Ministry of Justice. Country Development Strategy [Internet]. 2007. Available from: http://cbd.minjust.gov.kg/act/view/ru-ru/59205

104. Gleason GR, Sharmanov T. Anemia prevention and control in four Central Asian Republics and Kazakhstan. J Nutr [Internet]. 2002;132:867S-870S. Available from: http://libaccess.mcmaster.ca/login?url=http://ovidsp.ovid.com/ovidweb.cgi?T=JS&CSC=Y&NEWS=N&PAGE=fulltext&D=emed8&AN=34275100%0Ahttp://sfx.scholarsportal.info/mcmaster?sid=OVID:embase&id=pmid:11925500&id=doi:&issn=0022-3166&isbn=&volume=132&issue=4+SUPPL.

105. Good Health at Low Cost’: 25 years on. What makes a successful health system? [Internet]. Reproductive Health Matters. 2012. 212–214 p. Available from: https://www.tandfonline.com/doi/full/10.1016/S0968-8080%2812%2939614-6

106. Hardison C, Fonken P, Chew T, Smith B. The emergence of family medicine in Kyrgyzstan. Fam Med. 2007;39:627–33.

107. Volumes F, Summary E. Review of experience of family medicine in Europe and Central Asia. Volume IV: Kyrgyz Republic case study. Washington, D.C.; 2005. Report No.: 32354-ECA.

108. Kyrgyz Republic. Kyrgyz Republic National Health Care Reform Program “Manas Taalimi” for 2006-2010. Bishkek; 2006.

109. UNICEF, WHO. Country experiences with the baby-friendly hospital initative [Internet]. New York, New York; 2017. Available from: https://www.unicef.org/nutrition/files/BFHI_Case_Studies_FINAL.pdf

110. UNDP. The Kyrgyz Republic: The Second Progress Report on The Millennium Development Goals [Internet]. Bishkek, Kyrgyzstan; 2010. Available from: http://www.kg.undp.org/content/kyrgyzstan/en/home/library/mdg/the-first-millennium-development-goals-progress-report-in-the-ky.html

111. International Labor Organization, International Programme on the Elimination of Child Labour. Child Labour in Kyrgyzstan: An Initial Study. Bishkek; 2001.

112. UNICEF. UNICEF Annual Report 2015 - Kyrgyzstan. 2015;1–29.

113. USAID. USAID Quality Health Care Project [Internet]. 2015. Available from: https://www.usaid.gov/kyrgyz-republic/fact-sheets/usaid-quality-health-care-project

114. USAID, SPRING. Strengthening Nutrition within the Kyrgyz Republic Health System: SPRING in the Kyrgyz Republic (September 2017). Washington, D.C.; 2017.

115. U.S. Embassy in the Kyrgyz Republic. USAID presents results of Quality Health Care Project [Internet]. 2015. Available from: https://kg.usembassy.gov/usaid-presents-results-quality-health-care-project/

116. USAID. Kyrgyz Ministry of Health supports expansion of successful USAID pilot for ambulatory treatment of TB [Internet]. Available from: https://www.usaid.gov/sites/default/files/documents/1861/Kyrgyz Ministry of Health supports expansion of successful USAID pilot for ambulatory treatment of TB.pdf

117. USAID. USAID improves quality of TB laboratory services [Internet]. Kyrgyz Republic. Available from: https://www.usaid.gov/sites/default/files/documents/1861/USAID improves quality of TB laboratory services.pdf

118. USAID. Patients help each other complete TB treatment [Internet]. Kyrgyz Republic. Available from: https://www.usaid.gov/sites/default/files/documents/1861/Patients help each other complete TB treatment.pdf

119. BizExpert. AKIpress: Results of 25 years of independence of Kyrgyzstan [Internet]. [cited 2018 Jul 23]. Available from: http://www.bizexpert.kg/2016/09/15/akipress-itogi-25-let-nezavisimosti/

120. Agency 24.kg News. Regions of Kyrgyzstan: Who and where to live well [Internet]. [cited 2018 Jul 23]. Available from: https://24.kg/ekonomika/79340_regionyi_kyirgyizstana_komu_igde_jit_horosho/

121. Ministry of Justice of the Kyrgyz Republic. Convention on the Rights of the Child: Kyrgyzstan resolution No.1402-XII. 1994.

122. IRIN. Kyrgyzstan: The hidden threat of micronutrient deficiency. 2010.

123. UNICEF. UNICEF urges fortification of staple foods to improve child and maternal health and economic gains [Internet]. 2009 [cited 2018 Jul 23]. Available from: https://www.unicef.org/media/media_50067.html

124. UNICEF. Kyrgyzstan enacts law on flour fortification to fight ‘hidden hunger’ [Internet]. 2009 [cited 2018 Jul 23]. Available from: https://www.unicef.org/nutrition/kyrgyzstan_49274.html

125. UNICEF. Integrated management of childhood illness [Internet]. 2012 [cited 2018 Jul 23]. Available from: https://www.unicef.org/health/index_imcd.html

126. World Health Organization. What is Integrated Management of Childhood Illness (IMCI)? [Internet]. [cited 2018 Jul 23]. Available from: http://www.who.int/maternal_child_adolescent/child/imci/background/en/

127. 24.kg News. Allowance for the needy [Internet]. [cited 2018 Jul 23]. Available from: https://24.kg/obschestvo/24278_posobie_maloimuschim_ne_razgulyaeshsya_infografika/

1. (Bhutta, et al. 2013) [↑](#footnote-ref-1)
2. (Gillespie, et al. 2013) [↑](#footnote-ref-2)
3. (Janmohamed, Lassi and Bhutta 2018) [↑](#footnote-ref-3)
4. $1 USD = 62 KGS as of 1 July, 2015. [↑](#footnote-ref-4)
5. Heating boilers [↑](#footnote-ref-5)
6. Tiens – Chinese company which sells vitamins and biologically active supplements [↑](#footnote-ref-6)
7. Mother-in-law (from Kyrgyz language) [↑](#footnote-ref-7)
8. Small patties with a simple puff pastry and stuffed with mutton, onion and green herbs [↑](#footnote-ref-8)
9. Kyrgyz national soft drink, which is prepared only in hot months [↑](#footnote-ref-9)
10. Soup with rice and vegetables [↑](#footnote-ref-10)
11. A traditional meat dish consisting of finely chopped meat in a thinly rolled dough, steamed [↑](#footnote-ref-11)
12. Steamed roll of dough stuffed with finely chopped meat (sometimes minced meat) and / or vegetables (potatoes, pumpkin, etc.) [↑](#footnote-ref-12)
13. A dish of boiled rice with fat and pieces of meat (or fish) and spices [↑](#footnote-ref-13)
14. Traditional Kyrgyz dish with meat and dough [↑](#footnote-ref-14)
15. Salad from boiled beets, potatoes, carrots, as well as sauerkraut or pickled cucumbers [↑](#footnote-ref-15)
